# Novel MiRNA and PhasiRNA Biogenesis Networks in Soybean Roots from Two Sister Lines That Are Resistant and Susceptible to SCN Race 4

**DOI:** 10.1371/journal.pone.0110051

**Published:** 2014-10-30

**Authors:** Miaoyun Xu, Yinghui Li, Qiuxue Zhang, Tao Xu, Lijuan Qiu, Yunliu Fan, Lei Wang

**Affiliations:** 1 Biotechnology Research Institute, National Key Facility of Crop Gene Resources and Genetic Improvement, Chinese Academy of Agricultural Sciences, Beijing, China; 2 Institute of Crop Science, National Key Facility of Crop Gene Resources and Genetic Improvement, Chinese Academy of Agricultural Sciences, Beijing, China; Nanjing Agricultural University, China

## Abstract

The soybean cyst nematode (SCN), *Heterodera glycines*, is the most devastating pathogen of soybean worldwide. SiRNAs (small interfere RNAs) have been proven to induce the silencing of cyst nematode genes. However, whether small RNAs from soybean root have evolved a similar mechanism against SCN is unknown. Two genetically related soybean sister lines (ZP03-5373 and ZP03-5413), which are resistant and susceptible, respectively, to SCN race 4 infection were selected for small RNA deep sequencing to identify small RNAs targeted to SCN. We identified 71 less-conserved miRNAs-miRNAs* counterparts belonging to 32 families derived from 91 loci, and 88 novel soybean-specific miRNAs with distinct expression patterns. The identified miRNAs targeted 42 genes representing a wide range of enzymatic and regulatory activities. Roots of soybean conserved one TAS (Trans-acting siRNA) gene family with a similar but unique trans-acting small interfering RNA (tasiRNA) biogenesis profile. In addition, we found that six miRNAs (gma-miR393, 1507, 1510, 1515, 171, 2118) guide targets to produce secondary phasiRNAs (phased, secondary, small interfering RNAs) in soybean root. Multiple targets of these phasiRNAs were predicted and detected. Importantly, we also found that the expression of 34 miRNAs differed significantly between the two lines. Seven ZP03-5373-specific miRNAs were differentially expressed after SCN infection. Forty-four transcripts from SCN were predicted to be potential targets of ZP03-5373-specific differential miRNAs. These findings suggest that miRNAs play an important role in the soybean response to SCN.

## Introduction

Soybean (*Glycine max*) is an agronomically important crop that is rich in human dietary protein. The soybean cyst nematode (SCN), *Heterodera glycines* Ichinohe, is an obligate sedentary endoparasite that causes extensive damage to soybean worldwide and accounts for over one billion dollars of crop loss annually in the US [1]. These obligate parasites start their life cycles as non-feeding, mobile infective second-stage juveniles (J2) in soil that are able to locate and then penetrate into host roots [2]. The J2 then initiate the formation of specialized feeding sites called syncytia, which function as metabolic sinks to nourish the nematodes. In susceptible cultivars, nematodes depend entirely on functional syncytia to acquire nutrients to develop into reproductive adult males or females. The J2 also penetrate roots of resistant cultivars and initiate syncytia. However, resistance soon manifests as degeneration of the young syncytia and failure of the nematode to develop further [3]. Syncytium formation and maintenance are mediated through nematode signaling and are accompanied by changes in plant gene expression [4]. Identification of host plant genes and nematode genes that change expression, and may therefore be involved in plant–nematode interactions, would increase our understanding of the molecular mechanisms involved in this complex interaction, which will lead to the development of durable crop protection strategies. With the recent discovery of gene expression control of parasitism proteins via siRNA molecules [5], and recent advances in genomics, small RNAs (sRNAs), which are involved in the molecular mechanism of the soybean-SCN system, are now the focus of much research.

Endogenous sRNAs are known to be important regulators of gene expression at the transcriptional and post-transcriptional levels. In plants they are divided into several classes: trans-acting siRNAs (tasiRNAs), heterochromatin-associated siRNAs, natural antisense siRNAs (nat-siRNAs) and miRNAs [6]. These classes of non-coding RNAs are distinguished by their biogenesis pathways and the types of genomic loci from which they arise [7]. TasiRNA biogenesis from TAS loci depends on the miRNA-directed cleavage of their transcripts [8], [9]; indeed, three tasiRNA pathways have been characterized in *Arabidopsis* [8], [10]. Although miRNAs constitute only a small fraction of the sRNA population [11], [12], miRNA-guided post-transcriptional gene regulation is one of the most conserved and well-characterized gene regulatory mechanisms [11], [13], [14]. There is increasing evidence that miRNAs negatively regulate their target genes, which function in a wide range of biological processes, including organogenesis, signal transduction and stress responses [15], [16].

Plant miRNAs are generated from hairpin-structured non-coding transcripts and processed by Dicer such as DCL1 (DICER-LIKE 1), which cleaves a short (21-bp) duplex from the stem region [17]. The duplex is incorporated into an AGO complex and the miRNA* strand is subsequently degraded. The mature miRNA strand guides the AGO complex (RNA-induced silencing complex, RISC) to either protein-coding RNAs, which are cleaved by AGO at a specific position [18], or translational arrest [19]. Due to their evolutionary conservation, miRNAs have been found to exist in both plants [20], [21] and animals [22]–[24]. Conserved miRNA molecules can also be found in ferns, mosses and fungi [11]. To date, many miRNAs have been identified and deposited in miRBase V20.0 (http://www.mirbase.org/). Of these, 25,141 are mature miRNA products, from a total of 193 species. Comparative analysis indicates that some of the miRNA families are highly conserved among all plant species while others have diverged and evolved, generating abundant family- and species-specific miRNAs [11], [25], [26]. These dynamic and evolving miRNAs could serve as a driving force for the selection of improved and novel traits in plants.

As non-conserved or species-specific miRNAs are often expressed at a lower level than conserved miRNAs, many species-specific miRNAs have not been identified in small-scale sequencing projects. However, high-throughput sequencing technologies allow identification of many species-specific miRNAs in several species [10], [27]–[31]. Elucidating the function of these molecules requires effective approaches to identifying their targets. Recently, a new method called degradome sequencing, which combines high-throughput RNA sequencing with bioinformatic tools, has been used to screen for miRNA targets in *Arabidopsis* [32]–[34]. Using degradome sequencing, many of the previously validated and predicted targets of miRNAs and tasiRNAs have been verified [32], [33], [35], [36], indicating that this is an efficient strategy for identifying sRNA targets in plants on a large scale.

To determine if soybean has evolved sRNAs that repress the development and growth of SCN, and their potential targets, we selected the sister lines ZP03-5373 and ZP03-5413, which are resistant and susceptible, respectively, to SCN race 4 infection, and performed a comprehensive analysis of root miRNAs by deep sequencing, computational prediction and molecular approaches. Novel and conserved soybean miRNAs, tasiRNAs and phasiRNAs, and their partial targets were identified. Small RNAs upregulated by SCN infection were identified and the molecular regulation mechanism was discussed.

## Results

### sRNA population in soybean root

**Figure 1:**
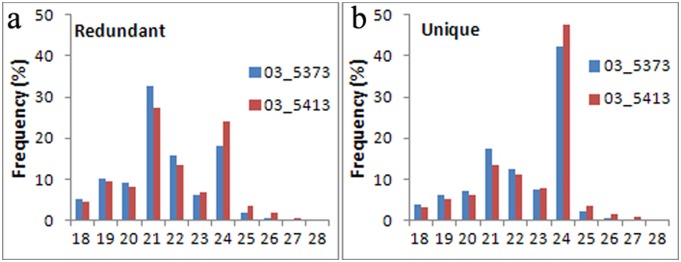
Length distribution of redundant and unique sRNA sequences. The length distribution of redundant and unique sRNAs in ZP03-5373 (a) and ZP03-5413 (b). The 21-nt of redundant is the predominant sRNA species and the 24-nt of unique is the most abundant.

To investigate the role of soybean miRNAs in response to SCN infection, two genetically related soybean sister lines (ZP03-5373 and ZP03-5413) were subjected to deep sequencing. The sister lines shared the same parents and displayed a different resistance to SCN race 4. Line ZP03-5373 exhibited high resistance to SCN race 4, whereas ZP03-5413 was susceptible to race 4. Two sRNA libraries from the roots of the sister lines were constructed and sequenced using Illumina GAIIx. A total of 15,101,204 sRNA raw reads were generated. After removing adaptor sequences, filtering out low quality reads and cleaning up sequences derived from adaptor–adaptor ligation, 7,903,242 and 5,931,837 reads, respectively, were obtained. These sRNAs consisted of 4,979,640 unique sequences (Table S1), which were matched to the public soybean genomic database (Soybean Genome V9.0, http://www.phytozome.net/index.php) using the SOAP program, leading to 3,409,866 genome-matched unique reads. These reads were subjected to further analysis (Table S1). The 20–24-nt sRNAs constituted over 80% of the identified soybean sRNAs, and the 21-nt class of sRNAs was the most abundant in both lines (Figure 1A). Notably, the expression of the unique 24-nt sRNAs was markedly higher than the 21-nt class in both lines (Figure 1B).

### Conserved and less-conserved miRNA families and their expression in soybean root

The reads (3 million) that mapped perfectly to the soybean genome were subjected to miRNA identification. miRBase 20.0, which contains 555 soybean mature miRNAs, was searched for known soybean miRNAs. As a result, a total of 420 known soybean mature miRNAs were identified from the two libraries, of which 364 were sequenced in both libraries; 26 miRNAs were detected in only ZP03-5373 and 30 in only ZP03-5413 (Table S2). Expression levels of the known miRNAs, as reflected by normalized reads (reads per million genome-matched reads, RPM), varied substantially among families in both lines. The highest read abundance (31,416 RPM and 20,776 RPM) was detected for gma-miR159, and was 2–25-fold greater than other relatively abundant miRNA families, including gma-miR396, gma-156, miR168, and miR166, whose total abundance ranged from 1,000 to 15,000 RPM (Table S2). Gma-miR862a was expressed in ZP03-5413 but not in ZP03-5373 (Table S2). Substantial variation was observed for gma-miR393, gma-miR398 and gma-miR399, whose abundance in ZP03-5373 was 10-fold greater than in ZP03-5413 (Table S2).

After excluding sRNA reads that perfectly matched known soybean miRNAs, the remaining 21 to 22 nt were subjected to rigorous secondary structural analysis of their precursors using RNAfold software (http://mfold.rna.albany.edu/). The minimum free energy (MFE) of the hairpin structure of the miRNA precursor was set to less than −25 kcal/mol. Those precursors with a canonical stem-loop structure were further analyzed by means of a series of stringent filter strategies to ensure that they met common criteria [37]. Precursors carrying both the miRNA-5p and miRNA miRNA-3p were then selected for further analysis. A total of 258 new soybean miRNA candidates that were not previously reported, including miRNA-5p and miRNA-3p, were identified from the two libraries, of which 132 were sequenced in both libraries. Eighty-seven miRNAs were detected in only ZP03-5373 and 39 in only ZP03-5413. Novel miRNA candidates were further assigned to miRNA families using sequences similar to other known miRNAs in the miRBase database (two or fewer mismatches). These miRNAs or families had been reported previously in some plant species or families, but are not conserved among angiosperm and coniferophyta lineages [26]. They were referred to as less-conserved miRNAs in this study.

**Figure 2:**
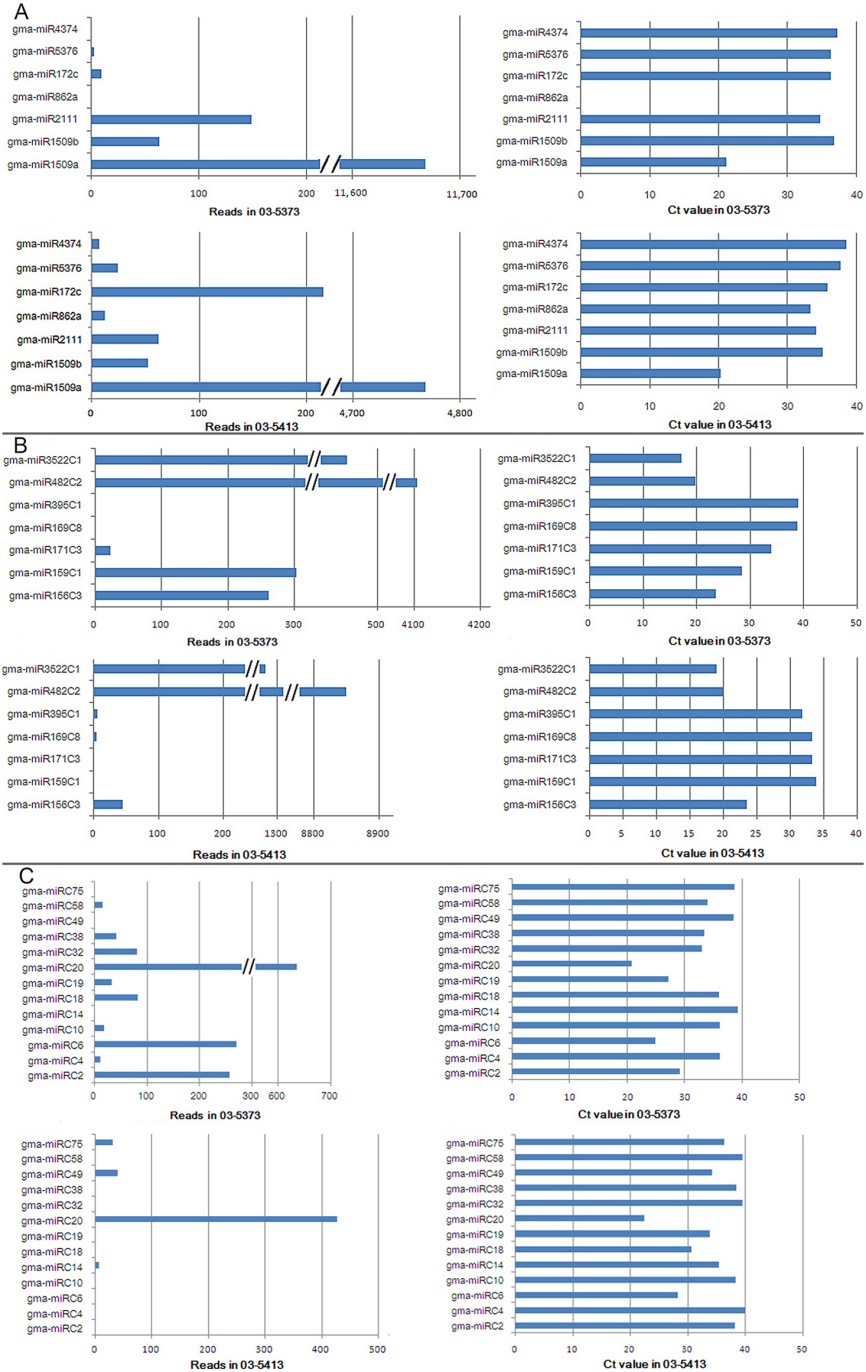
Expression levels of gma-miRNAs by two methods. Profile of sequencing frequencies for gma-miRNAs (left column of A, B and C); Profile of qRT-PCR Ct values for gma-miRNAs (right column of A, B and C).

A total of 71 miRNAs-miRNAs* counterparts belonging to 32 families derived from 91 loci (Table 1 and Figure S1), that had previously been identified and reported in at least one plant species or family [11] were identified from the 258 miRNA candidates. A canonical predicted stem-loop structure could be identified in all 32 less-conserved miRNA families (Figure S1). Overall, all less-conserved miRNAs displayed lower expression levels than the conserved miRNAs, with the exception of gma-miR482C2, which was expressed at abundances of 4,000 RPM and 8,000 RPM in ZP03-5373 and ZP03-5413, respectively (Table 1). However, as with the conserved miRNAs, some of the less-conserved miRNAs were expressed differentially between ZP03-5373 and ZP03-5413. For example, ZP03-5413-biased expression was observed for gma-miR395C1, while ZP03-5373-biased expression was apparent for gma-miR393C1 and gma-miR2109C1 (Table 1). To validate the miRNA RPM data, we performed stem-stoop-based qRT-PCR analysis for selected miRNAs representing conserved, less-conserved and soybean-specific (discussed below) examples in the two lines. We found that while the qRT-PCR results for most of the miRNAs (miR1509a, miR1509b, miR2111 (Figure 2A) and miR395C1 (Figure 2B), miRC2, miRC6, miRC20 (Figure 2C), etc.) were reflective of the relative abundances of the sequenced RNAs in the two lines, others displayed varying degrees of divergence between the two analyses. For example, the miRC18 RPM value for ZP03-5373 was fourfold higher than for miRC10, while the abundances of miRC18 and miRC10 were in agreement, based on qRT-PCR results (Figure 2C). For miR482C2 the opposite pattern between qRT-PCR and miRNA sequencing was observed (Figure S2), which may have resulted from deep-sequencing deviation.

**Table 1:** New members of conserved and less-conserved soybean miRNAs.

							Precursor position		5373			5413		
Name	miRNA-5p sequence	nt	Locus	Str	miRNA-3p sequence	nt	start	end	E^ a^	re^ b^	re^ c^	E ^d^	re^ e^	re^ f^
**gma-miR156C1**	ctgacagaagatagagagcac	21	Gm18	-	gctctctagtcttctgtca	19	61442581	61442703		0	0	▴	266	2
**gma-miR156C2**	gtgacagaagagagtgagcac	21	Gm04	+	gctcactctctatctgtcacc	21	4257055	4257167	•	4	109	▴	3	51
**gma-miR156C3**	tgacagaagagagtgagcaca	21	Gm17	-	gctcacttctctatctgtcagc	22	6149953	6150085	•	15	2066	▴	2	268
**gma-miR156C4**	tgacagaagagagtgagcaca	21	Gm02	+	gctcacttctctatctgtcagc	22	41864163	41864265	•			▴		
**gma-miR156C5**	tgacagaagagagtgagcaca	21	Gm06	-	gctcacttctctttctgtcaac	22	4699136	4699258	•	15	410	▴	30	308
**gma-miR156C6**	tgacagaagagagtgagcaca	21	Gm04	+	gctcacttctctttctgtcaac	22	4990845	4990967	•			▴		
**gma-miR156C7**	gtgacagaagagagtgagcac	21	Gm14	+	gctcattctctatctgtcacc	21	9431596	9431718	•	4	1706	▴	3	402
**gma-miR156C8**	gtgacagaagagagtgagcac	21	Gm17	-	gctcattctctatctgtcacc	21	4291652	4291764	•			▴		
**gma-miR156C9**	gtgacagaagagagtgagcac	21	Gm17	-	gcttactctctatctgtcacc	21	38431865	38431977	•		949	▴	3	206
**gma-miR156C10**	atgacagaagagagtgagcac	21	Gm06	+	gcttactctctatctgtcatc	21	4013568	4013680	•	6	391	▴	128	17
**gma-miR157C1**	acagaagatagagagcacaga	21	Gm07	+	gctctctaagcttctgtcatc	21	9347121	9347273	•	44	9	▴	43	5
**gma-miR157C2**	acagaagatagagagcacaga	21	Gm09	-	gctctctaggcttctgtcatc	21	37843733	37843885				▴		8
**gma-miR157C3**	acagaagatagagagcacaga	21	Gm05	-	gctctctatacttctgtcatc	21	38621682	38621814	•		1334	▴		1094
**gma-miR157C4**	acagaagatagagagcacaga	21	Gm02	+	tgctctctagtcttcttgtcatc	23	7812528	7812630	•		2	▴		2
**gma-miR157C5**	ctgacagaagatagagagcac	21	Gm18	-	gctctctagtcttctgtcatc	21	61442581	61442703	•	12	276		0	0
**gma-miR159C1**	agctgcttagctatggatccca	22	Gm09	+	cttccatatctggggagcttc	21	37672401	37672593	•	12	2401		0	0
**gma-miR159C2**	agctgcttagctatggatccca	22	Gm07	-	cttccatatctggggagcttc	21	9524917	9525129	•				0	0
**gma-miR160C1**	gtgcctggctccctgtatgcc	21	Gm19	-	cgtgcgaggagccatgcatg	20	43795945	43796047	•	152	3		0	0
**gma-miR160C2**	tgcctggctccctgaatgcca	21	Gm15	-	gcatgaggggagtcatgcagg	21	9547165	9547297	•	436	271	▴	561	5
**gma-miR162C1**	tggaggcagcggttcatcgat	21	Gm05	-	tcgataaacctctgcatccagc	22	7692586	7692708	•	62	2	▴	16	39
**gma-miR162C2**	tggaggcagcggttcatcgatc	22	Gm17	+	tcgataaacctctgcatccagc	22	10181486	10181608	•			▴	16	
**gma-miR162C3**	ggatgcagcggttcatcgatc	21	Gm06	-	ggatgcagcggttcatcgatc	21	20176237	20176339	•	40	2	▴	11	
**gma-miR164C1**	tggagaagcagggcacatgct	21	Gm07	+	cttgtgtcctacttctccagc	21	3508920	3509002	•	24	2		0	0
**gma-miR166C1**	ggaatggtgtctggttcgaga	21	Gm20	-	tcggaccaggcttcattccccc	22	43105388	43105500	•	110	44	▴	86	9
**gma-miR166C2**	ggaatggtgtctggttcgaga	21	Gm10	+	tcggaccaggcttcattccccc	22	41243362	41243474	•			▴		
**gma-miR166C3**	aatgttgtttggctcgaggta	21	Gm08	+	ctcggaccaggcttcattccc	22	14990528	14990750	•	3	18		0	0
**gma-miR166C4**	ggaatgttgtctggctcgagga	22	Gm16	-	tcggaccaggcttcattccccc	22	1912569	1912721	•	7	44		0	0
**gma-miR167C1**	tgaagctgccagcatgatctta	22	Gm10	-	agatcatgtggcagtttcacc	21	46574250	46574362	•	1405	44	▴	744	15
**gma-miR167C2**	tgaagctgccagcatgatctta	22	Gm20	+	agatcatgtggcagtttcacc	21	37901892	37902004	•			▴		
**gma-miR168C1**	ttcgcttggtgcaggtcgggaa	22	Gm09	-	cccgccttgcatcaactgaat	21	41353225	41353347	•	2	981	▴	4	385
**gma-miR168C2**	ttcgcttggtgcaggtcgggaa	22	Gm01	-	cccgccttgcatcaactgaat	21	48070302	48070424	•			▴		
**gma-miR169C1**	agccaaggatgacttgccggc	21	Gm09	+	ggcaagttgtgtttggctat	20	35771781	35771943	•	317	2	▴	237	4
**gma-miR169C2**	agccaaggatgacttgccggc	21	Gm10	-	ggcaagttggccttggctat	20	40332783	40332935	•		22		0	0
**gma-miR169C3**	agccaaggatgacttgccggc	21	Gm15	+	ccggcgagacatcttggctca	21	14191164	14191316	•		16	▴		5
**gma-miR169C4**	agccaaggatgacttgccggc	21	Gm09	+	ccggcgagacatcttggctca	21	5282105	5282217	•		16	▴		5
**gma-miR169C5**	agccaaggatgacttgccggc	21	Gm09	+	ggcaggttatcctgtggctac	21	5299562	5299754	•		9		0	0
**gma-miR169C6**	agccaaggatgacttgccggc	21	Gm15	+	agcgagacatccttgttcact	21	14194104	14194226	•		75	▴		25
**gma-miR169C7**	agccaaggatgacttgccggc	21	Gm15	+	ggtgagacatcttgactcact	21	14188499	14188621		0	0	▴		2
**gma-miR169C8**	agccaagggtgatttgccggc	21	Gm15	+	ggcaagtttctcttggctac	20	14150054	14150196		0	0	▴	33	28
**gma-miR171C1**	tgttggaacagttcaatcaaa	21	Gm08	-	tgattgagccgtgccaatatca	22	921788	921900	•	60	23	▴	6	18
**gma-miR171C2**	tgttggcttggctcaatcaaa	21	Gm16	-	tgattgagccgtgccaatatca	22	5347841	5347933		0	0	▴	17	
**gma-miR171C3**	agatattggtacggttcaatc	21	Gm15	-	ttgagccgtgccaatatcacat	22	8464103	8464215	•	188	4		0	0
**gma-miR171C4**	agatattggtacggttcaatc	21	Gm13	+	ttgagccgtgccaatatcacat	22	30650787	30650899	•				0	0
**gma-miR172C1**	ggagcatcatcaagattcaca	21	Gm18	+	gggaatcttgatgatgctgca	21	2968997	2969129		0	0	▴	41	5
**gma-miR172C2**	ggagcatcatcaagattcaca	21	Gm14	+	gggaatcttgatgatgctgca	21	5548763	5548895		0	0	▴		
**gma-miR172C1**	gtagcatcatcaagattcaca	21	Gm13	-	gagaatcttgatgatgctgcat	22	40401673	40401825	•	168	2		0	0
**gma-miR172C3**	cagcagcatcaagattcacac	21	Gm10	+	tgagaatcttgatgatgctgc	21	43474729	43474831	•	2	41	▴	3	20
**gma-miR172C4**	cagcagcatcaagattcacac	21	Gm20	-	tgagaatcttgatgatgctgc	21	40895738	40895850	•			▴		
**gma-miR172C5**	gcagcaccatcaagattcaca	21	Gm10	-	tgagaatcttgatgatgctgc	21	31592562	31592704	•			▴	3	
**gma-miR319C1**	agagcttccttcagtccactc	21	Gm14	+	ttggactgaagggagctccctc	22	47959347	47959549	•	99	22	▴	12	76
**gma-miR319C2**	agagcttccttcagtccactc	21	Gm02	+	ttggactgaagggagctccctc	22	45704224	45704416	•			▴		
**gma-miR319C3**	agagcttccttcagtccactc	21	Gm18	-	ttggactgaagggagctccctt	22	4278867	4279079	•	50	1216	▴	12	1207
**gma-miR319C4**	agagcttccttcagtccactc	21	Gm11	+	ttggactgaagggagctccctt	22	32902053	32902265	•			▴		
**gma-miR319C5**	agagctttcttcagtccactc	21	Gm05	+	ttggactgaagggagctccctt	22	40832090	40832292	•	31		▴	5	
**gma-miR319C6**	agagctttcttcagtccactt	21	Gm08	-	ttggactgaagggagctccctt	22	1647797	1647999	•	27		▴	2	
**gma-miR319C7**	agagctctcttcagcccactca	22	Gm11	+	ttggactgaagggagctccctt	22	1374016	1374208	•	5		▴	7	
**gma-miR390C1**	aaagctcaggagggatagcgcc	22	Gm18	+	cgctacccatcctgagtttca	21	53278033	53278165	•	3	19	▴	7	15
**gma-miR390C2**	aaagctcaggagggatagcgcc	22	Gm03	-	aaagctcaggagggatagcgcc	22	6558180	6558282	•		898	▴		306
**gma-miR390C3**	aaagctcaggagggatagcgcc	22	Gm01	+	aaagctcaggagggatagcgcc	22	42335602	42335724	•			▴		
**gma-miR390C4**	aaagctcaggagggatagcgcc	22	Gm18	-	cgctatctatcctgagtttca	21	5047763	5047875	•		537	▴		362
**gma-miR390C5**	aaagctcaggagggatagcgcc	22	Gm11	+	cgctatctatcctgagtttca	21	30272752	30272864	•			▴		
**gma-miR390C6**	aagctcaggagggatagcacca	22	Gm02	+	cgctatctatcttgagcttca	21	44954747	44954859	•	5	34		0	0
**gma-miR393C1**	tccaaagggatcgcattgatct	22	Gm16	+	tcatgcgatcccttaggaact	21	33891068	33891230	•	3323	28	▴	55	69
gma-miR395C1	gtttccctgaacacttcatt	20	Gm02	-	tgaagtgtttgggggaactcc	21	1723444	1723546		0	0	▴	11	36
gma-miR395C2	agttcctctgaatgcttcata	21	Gm02	+	tgaagtgtttgggggaactcc	21	1730681	1730793		0	0	▴	16	0
gma-miR395C3	gttccccttaatgcttcattg	21	Gm08	+	tgaagtgtttgggggaactcc	21	40840211	40840333		0	0	▴	9	0
gma-miR395C4	agttcctctgaacgcttcat	20	Gm01	-	tgaagtgtttgggggaactcc	21	4813256	4813388		0	0	▴	2	0
gma-miR395C5	gttccctcgaacacttcaacg	21	Gm18	-	ctgaagtgtttgggggaaccc	21	16316060	16316182		0	0	▴	20	8
gma-miR395C6	gttcctcttaacgcttcattg	21	Gm18	-	ctgaagtgtttgggggagctt	21	16305078	16305190	•	22	220	▴	12	70
gma-miR399C1	gtgcaattctcctttggcagg	21	Gm15	+	tgccaaaggagaattgccctg	21	6547375	6547497	•	7	14		0	0
gma-miR399C2	gggcatgtctcttttggcagg	21	Gm16	+	tgccaaaggagagctgccctg	21	35606648	35606790	•	69	237	▴	32	89
gma-miR399C3	tgccaaaggagatttgccctg	21	Gm05	+	gagcaaatctccagtggcaga	21	34951411	34951523	•	153	19		0	0
gma-miR399C4	tgccaaaggagatttgccctg	21	Gm08	+	gagcaaatctccattggcagt	21	9114310	9114422	•		31		0	0
gma-miR399C5	gggctcctctctcctggcatg	21	Gm20	-	tgccaagggagagttgccctg	21	38248027	38248139	•	12	63		0	0
gma-miR399C6	gggctcctctctcctggcatg	21	Gm10	+	tgccaagggagagttgccctt	21	46279386	46279498	•		40		0	0
gma-miR399C7	gggcttctctttattggcagg	21	Gm20	-	ttgccaaaggagagttgccctg	22	38251187	38251279	•	537	14		0	0
gma-miR399C8	gggcttctctttattggcagg	21	Gm10	+	ttgccaaaggagagttgccctg	22	46275313	46275405	•				0	0
gma-miR408C1	ctgggaacaggcagggcacga	21	Gm03	-	atgcactgcctcttccctggct	22	44626689	44626841	•	38	3	▴	122	51
gma-miR479C1	cgtgatattggtacggctcatc	22	Gm06	-	cgagccgaatcaatatcactct	22	10859604	10859716	•	185	10	▴	133	3
gma-miR479C2	cgtgatattggtacggctcatc	22	Gm04	+	cgagccgaatcaatatcactct	22	46988567	46988679	•					
gma-miR482C1	ggaatgggctgattgggaagt	21	Gm18	-	tcccaattccgcccattcctatga	24	61452891	61453023	•	1010	3	▴	2720	2
gma-miR482C2	ggaatgggctgattgggaagc	21	Gm02	+	tcccaattccgcccattcctatga	24	7783795	7783937	•	32443	3	▴	52497	2
gma-miR862C1	tccctcaaaggcttccagtat	21	Gm08	+	gctggatgtctttgaaggaac	21	46853887	46854009	•	1161	8	▴	537	2
gma-miR1509C1	ttaatcaaggaaatcacggttg	22	Gm05	-	actgtgtttccttggttaaag	21	7774097	7774209	•	26429	39	▴	7837	27
gma-miR1510C1	gagggataggtaaaacaactact	23	Gm02	+	tgttgttttacctattccacca	21	6599288	6599400	•	2	1915	▴	4	337
gma-miR1514C1	ttcatttttaaaataggcattg	22	Gm07	-	atgcctattttaaaatgaaaa	21	43175789	43175931	•	1038	32	▴	494	19
gma-miR1514C2	attcccctgaccacttcatta	21	Gm01	-	ttgaagtgttttggggaactc	21	4760437	4760549		0	0	▴	2	39
gma-miR2109C1	tgcgagtgtcttcgcctctga	21	Gm04	-	ggaggcgtagatactcacacc	21	28532441	28532543	•	21019	9943	▴	4187	9027
gma-miR2118C1	gggagatgggagggtcggtaaa	22	Gm10	-	ttgccgattccacccattccta	22	48573991	48574163	•	17	29035	▴	2	8825
gma-miR3522C1	tgagaccaaatgagcagctga	21	Gm15	+	agctgctcatctgttctcagg	21	4318762	4318894	•	3592	340	▴	7602	207
gma-miR4416C1	tgggtgagagaaacacgtatt	21	Gm02	-	acgggtcgctctcacctggag	21	30498947	30499129	•	94	2		0	0
gma-miR5037C1	cctcaaaggcttccactactt	21	Gm18	-	tggtggaactttgaggctt	19	61631519	61631621	•	55	2		0	0
gma-miR5044C1	cctcaaaggcttccactactgcat	24	Gm08	+	gtagtggatgcctggaggtcc	21	46838000	46838122	•	2	363	▴	8	264

Bold means conserved miRNAs and no bold means less-conserved miRNAs; E^a^(•): the miRNAs in ZP03-5373;re^b^: miRNA-5P reads of 5373(normalized reads per million reads,RPM); re^c^: miRNA-3P reads of 5373 (RPM); E^d^(▴): the miRNAs in ZP03-5413;re^e^: miRNA-5P reads of 5413 (RPM);re^f^: miRNA-3P reads of 5413 (RPM). Empty apace means the same number with the previous row.

### Prediction and validation of novel soybean-specific miRNAs

Because numerous species-specific miRNAs considered to be of a more recent evolutionary origin [11] have been identified in other species, soybean is likely to have evolved unique miRNAs. After excluding sRNA reads homologous to known miRNAs or families (two or fewer mismatches, miRBase 20), the remaining 21–22-nt sRNAs were subjected to rigorous secondary structural analysis of their precursors using the RNAfold software (http://mfold.rna.albany.edu/). Those precursors with a canonical stem-loop structure were further analyzed by means of a series of stringent filter strategies to ensure that they met the criteria established by the research community [37]. A total of 74 miRNA family candidates derived from 88 loci (Table 2 and Figure S3) met the screening criteria, of which all had miRNA star (miRNA*) sequences identified from the same libraries. We termed these soybean-specific miRNAs. Of the 88 soybean-specific miRNAs, 75 belonged to the 21-nt class and 13 to the 22-nt class (Table 2 and Figure S2). In general, the soybean-specific miRNAs were less abundant than the conserved and less-conserved miRNAs in the two lines examined. For example, only gma-miRC20 displayed a read abundance above 600 RPM in ZP03-5373, while 50% of the 88 miRNA family candidates yielded levels below 10 RPM (Table 2). This low level of expression was confirmed by stem-loop qRT-PCR analysis. (Figure 2C). As reported above for conserved miRNAs, the RPM values of some soybean-specific miRNAs corresponded to their relative abundance determined by miRNA qRT-PCR (gma-miR2 and gma-miR20, *etc.*), but several exhibited divergence (*e.g.*, gma-miRC4, gma-miRC14 and gma-miRC32 (Figure 2C and Figure S2)).

**Table 2:** Candidate soybean-specific miRNAs.

							Precursor position		5373			5413		
Name	miRNA-5p sequence	nt	Locus	Str	miRNA-3p sequence	nt	start	end	E^a^	Re^b^	Re^c^	E^d^	Re^e^	Re^f^
gma-miRC1	aaagatggtgctgacgtcgac	21	Gm01	+	tgatgtcagcaccgtctttga	21	5506384	5506536	•	2	1		0	0
gma-miRC2	aaatcatgactttctcttgta	21	Gm20	-	tatgagagaaagccatgactt	21	223675	223767	•	257	16		0	0
gma-miRC3	actctccctcaagggcttctcg	21	Gm08	+	tagaggcccttggggaggagta	22	1771387	1771489		0	0	▴	3	0
gma-miRC4	actgctattcccatttctaaa	21	Gm16	+	tagaaagggaaatagcagttga	22	32903724	32903836	•	11	2		0	0
gma-miRC5	agagatgtatggagtgagaga	21	Gm17	+	tctcattccatacatcgtctgac	23	6190584	6190716	•	101	5	▴	84	1
gma-miRC6	agaggtgtatggagtgagaga	21	Gm13	+	tctcattccatacatcgtctgac	23	25849768	25849890	•	270	5		0	0
gma-miRC7	agccaagggtgatttgccggc	21	Gm15	+	cggcaagtttctcttggctac	21	14150045	14150207	•	7	1		0	0
gma-miRC8	aagtgttgctaacagagttta	21	Gm17	+	agctctgttggctacactttg	21	41783743	41783905	•	11	82	▴	11	14
gma-miRC9	aggagcttccctcagcccatt	21	Gm14	-	tggactgaagggagctccttct	22	45953431	45953653		0	0	▴	2	1
gma-miRC10	atacatatcgtgttgccaagc	21	Gm13	+	aaggcagaacgatatgtacgcaga	24	41358317	41358479	•	17	1		0	0
gma-miRC11	aatgttgtttggctcgaggta	21	Gm08	+	atctcggaccaggcttcattcc	22	14990538	14990740		0	0	▴	0	2
gma-miRC12	cagctacatgttttaccatct	21	Gm14	-	atggtgatacatgtagttgca	21	6304109	6304381	•	1	11		0	0
gma-miRC13	attagaaatcacctattttga	21	Gm15	+	aaaattggtggtttccaataa	21	42969095	42969237	•	2	0		0	0
gma-miRC14	attagctaattcgtagaagct	21	Gm10	-	catctacaaattagctaatgg	21	10090333	10090465		0	0	▴	6	1
gma-miRC15	attgacagaagagagtgagcac	22	Gm14	-	gctcaccactctttctgtcggtt	23	10664497	10664609	•	4	1	▴	8	2
gma-miRC16	attagctaatttgtagaagtt	21	Gm08	-	catctacaaattagctaatgg	21	2218171	2218293	•	1	2		0	0
gma-miRC17	attagctaatttgtagaagtt	21	Gm13	+	catctacaaattagctaatgg	21	6220748	6220840	•				0	0
gma-miRC18	gggaagacatgggtatggggg	21	Gm10	-	cccataccactgtttttcctc	21	48569602	48569754	•	1	81		0	0
gma-miRC19	cctaactgaaaattcttaaagt	21	Gm18	+	tttaagaatttcagttatgca	21	60819567	60819659	•	32	5		0	0
gma-miRC20	agaggtgtttgggatgagaga	21	Gm09	-	cctcattccaaacatcatctaa	22	16565915	16566037	•	64	635	▴	52	426
gma-miRC21	aatcaaggaaatcacggtcgcg	22	Gm17	+	cgaccgtgtttccttggttaa	21	10099753	10099885	•	36	2	▴	17	4
gma-miRC22	agccaaggatgacttgccgga	21	Gm17	-	cggcaagtaatctttggctgc	21	4864155	4864297	•	1	1	▴	1	3
gma-miRC23	gtgcctggctccctgtatgcc	21	Gm03	-	cgtgcgaggagccatgcatgc	21	41268398	41268510		0	0	▴	1	5
gma-miRC24	tctgcatcctgaggtttagag	21	Gm18	-	ctaatccttgggatgcagatt	21	49962676	49962778	•	2	2		0	0
gma-miRC25	ctaatctgcatcctgaggttt	21	Gm07	-	tccttgggatgcagattatct	21	16402343	16402445	•	4	1		0	0
gma-miRC26	ctatacaactatacatggatg	21	Gm02	-	ttcatgtatgattgtatgtct	21	7455342	7455504	•	2	1		0	0
gma-miRC27	acaaagcccccgagtgaagaa	21	Gm19	+	ctccactcgaggactttgtcc	21	41390254	41390386	•	0	2	▴	1	4
gma-miRC28	cttagattttgggttttggtc	21	Gm04	+	cccaaactcaaattctaagaa	21	7505150	7505252	•	2	0		0	0
gma-miRC29	gcaccaatccgtggttcttcct	21	Gm18	+	gaagagccacagattggtgctg	22	17786331	17786523	•	1	1		0	0
gma-miRC30	gaatgttgtctggctcgagga	21	Gm16	-	gtcggaccaggcttcattccc	21	1912569	1912721		0	0	▴	3	1
gma-miRC31	gagctttatggatcacctgat	21	Gm01	-	caggtgattcgtaaaactcac	21	55781589	55781691	•	15	2		0	0
gma-miRC32	gagttccctgcactccaagtct	22	Gm16	-	attggagtgaagggagctccaga	23	2794127	2794309	•	80	0		0	0
gma-miRC33	ccaaagggatcgcattgatccc	22	Gm11	+	gatcatgctatccctttggat	21	36567510	36567642	•	2	96	▴	3	75
gma-miRC34	ccaaagggatcgcattgatccc	22	Gm18	-	gatcatgctatccctttggat	21	2409738	2409860		0	0		0	0
gma-miRC35	ccaaagggatcgcattgatccc	22	Gm02	-	gatcatgctatccctttggat	21	47136196	47136328		0	0		0	0
gma-miRC36	acagaagatagagagcacaga	21	Gm09	-	gctctctaggcttctgtcatcc	22	37843733	37843885	•	6	12		0	0
gma-miRC37	tgccaagtaaatgtgaaaagta	21	Gm20	-	gcttttctatttattgtggca	21	46584855	46584987		0	0	▴	0	2
gma-miRC38	ggccaaacctaaaagattcca	21	Gm08	+	gaaactcttaagtttggcctt	21	13130608	13130690	•	41	23		0	0
gma-miRC39	agaggcctgattccatagccat	22	Gm05	+	ggctctgtgaatctgtctccga	22	35743158	35743320	•	0	2		0	0
gma-miRC40	gggaaggcatgggtatggggg	21	Gm20	+	cccataccactgtttttcctc	21	35360269	35360441	•	133	81	▴	127	44
gma-miRC41	gggcacctctctcctggcagg	21	Gm09	+	ttgccaaaggagagttgccctg	22	34181516	34181638	•	4	2		0	0
gma-miRC42	gggcacctctctcctggcagg	21	Gm16	+	ttgccaaaggagagttgccctg	22	35612504	35612616	•				0	0
gma-miRC43	ggttcgtgcgtgaatctaatc	21	Gm10	+	ttagattcacgcacaaacttgt	22	1085226	1085328	•	1	0		0	0
gma-miRC44	gtggtatcaggtcctgcttca	21	Gm18	+	aaccaggctctgataccatgg	21	21161231	21161333	•	35	31	▴	55	37
gma-miRC45	gttccccttaatgcttcattg	21	Gm08	+	actgaagtgtttgggggaact	21	40840221	40840313	•	2	0		0	0
gma-miRC46	atcagtagcatcatcatcaaa	21	Gm07	+	gtttgatgatgatgttaccga	21	10004506	10004628	•	1	12		0	0
gma-miRC47	atcagtagcatcatcatcaaa	21	Gm14	+	gtttgatgatgatgttaccga	21	13818986	13819108		0	0		0	0
gma-miRC48	atcagtagcatcatcatcaaa	21	Gm07	+	gtttgatgatgatgttaccga	21	10001904	10002006		0	0		0	0
gma-miRC49	gtttgtaaatcatgactttct	21	Gm20	-	gaaagccatgacttacacacgc	22	223675	223767		0	0	▴	40	2
gma-miRC50	taagacggtaatgtccccaaa	21	Gm12	+	tggggacataaccgtcttaga	21	25767259	25767361	•	2	1	▴	2	1
gma-miRC51	tagatcaatagagcttaagag	21	Gm05	-	cgtaagctctattgatctatt	21	32896903	32897065		0	0	▴	4	1
gma-miRC52	tatgagagaaagccatgactt	21	Gm17	-	gtcatggcattatctcatatc	21	1401425	1401527	•	16	1		0	0
gma-miRC53	tcaatctgaatacatgactatt	22	Gm13	-	cagccatgtactttgattgagc	22	39325490	39325602		0	0	▴	6	1
gma-miRC54	tcacgcctaatcactgacgca	21	Gm03	+	tgttagtgataaggcgtgatgatg	24	25186806	25187048	•	11	1		0	0
gma-miRC55	tcattgagtgcagcgttgatga	22	Gm08	-	atcgacactgcactcaatcatg	22	4639027	4639169	•	8	1	▴	5	1
gma-miRC56	gaacgatttgatggtttggaat	22	Gm13	-	tcccaagcaacgagtcttcggt	22	2155583	2155665		0	0	▴	0	13
gma-miRC57	gaacgatttgatggtttggaat	22	Gm08	+	tcccaagcaacgagtcttcggt	22	14019990	14020072		0	0	▴	0	0
gma-miRC58	tctccctcaagggcttctcgct	22	Gm08	+	ctagaggcccttggggaggagt	22	1771404	1771486	•	15	3		0	0
gma-miRC59	tctcttgggtgcattgtaatt	21	Gm01	-	ttacaaatgcacgcaagaaatc	22	39527403	39527505	•	3	0		0	0
gma-miRC60	agacatcaccacaaacaagtc	22	Gm19	+	tcttgtttgtggtgatgtctag	22	43786812	43786914	•	1	16	▴	1	4
gma-miRC61	tgaaaaattcatggatcagtt	21	Gm08	-	atcctaggacttttcatcttc	21	27936700	27936802	•	4	1		0	0
gma-miRC62	agttcctctgaacgcttcatg	21	Gm01	-	tgaagtgtttgggggaactct	21	4810812	4810944	•	10	33	▴	10	128
gma-miRC63	agttcctctgaacgcttcatg	21	Gm02	+	tgaagtgtttgggggaactct	21	1736337	1736459	•			▴		
gma-miRC64	agttcctctgaacgcttcatg	21	Gm02	+	tgaagtgtttgggggaactct	21	1750582	1750684	•			▴		
gma-miRC65	agttcctctgaacgcttcatg	21	Gm01	-	tgaagtgtttgggggaactct	21	4797899	4798021	•			▴		
gma-miRC66	tgagctaaggatgacttgccgg	22	Gm09	+	agcaagacatcctttctcact	21	5287902	5288004	•	5	0		0	0
gma-miRC67	ggttagctcaaggatctcaca	21	Gm16	+	tgatatccttgagctaataca	21	35590506	35590718	•	3	4	▴	3	6
gma-miRC68	tgaaaaattcatggatcagt	21	Gm01	-	tgatccaggaacttttcatct	21	24948431	24948533	•	1	8	▴	1	7
gma-miRC69	tgcctcaatctgaatacatga	21	Gm13	-	atgtactttgattgagccgcg	21	39325490	39325602	•	6	0		0	0
gma-miRC70	tgcgggtatctttgcctctga	21	Gm04	-	agtggcgtagatccccacaaca	22	28578959	28579081	•	30	0	▴	25	1
gma-miRC71	aactggaaattcttaaagcatt	21	Gm02	-	tgctttaagaatttcagttat	21	8618676	8618788	•	0	25	▴	1	2
gma-miRC72	tatcttggatcacagccccattg	21	Gm18	+	tggggcttgatccaagatagg	21	10413939	10414031	•	0	9	▴	47	2
gma-miRC73	tgttggcttggctcaatcaaa	21	Gm16	-	tgattgagccgtgccaatatca	22	5347841	5347933	•	7	0		0	0
gma-miRC74	tgttgtaagcacatctgagtc	21	Gm16	-	ctcagttgtacttacaacaca	21	31233995	31234117	•	2	0	▴	5	1
gma-miRC75	ttaaagtgcttcactttgtgg	21	Gm04	+	acaaagtgaagcactctaaca	21	869303	869405		0	0	▴	30	0
gma-miRC76	ttaaggtattggcgtgcctca	21	Gm12	+	agccgcgtcaatatcttattt	21	35489085	35489197	•	14	0	▴	3	1
gma-miRC77	ttagcttctttcacctttccc	21	Gm17	-	gtgagaggtgaaggaagctaa	21	14170501	14170623	•	0	8		0	0
gma-miRC78	ttcatttttaaaatagacattg	22	Gm17	+	atgcctattttaaaatgaaaa	21	1497604	1497836	•	39	4	▴	35	3
gma-miRC79	atgttggtgaggttcaatccga	22	Gm13	+	ttgagccgcgccaatatcactt	22	26271133	26271245	•	1	8	▴	1	3
gma-miRC80	atgttggtgaggttcaatccga	22	Gm17	-	ttgagccgcgccaatatcactt	22	9101688	9101800	•			▴		
gma-miRC81	tggagggataggtaaaacaatg	22	Gm16	+	ttgttttacctattccacccat	22	31518896	31519008	•	16	94	▴	18	48
gma-miRC82	tttaatgaaatgttttctgtt	21	Gm08	-	tagaaaacatttccttaaacc	21	10928837	10929089		0	0	▴	7	1
gma-miRC83	tttatcagtagcatcatcatc	21	Gm07	+	tgatgatgttaccgataatga	21	10001904	10002006		0	0	▴	10	0
gma-miRC84	taaccattcattttcatgaaa	21	Gm04	-	tttcaagaaaatgaatggtga	21	5771445	5771537		0	0	▴	1	5
gma-miRC85	aatgtcgtttggttcgagatc	21	Gm10	-	tttcggaccaggcttcattcc	21	2905311	2905423	•	4	114	▴	2	61
gma-miRC86	gaatgttgtctggctcgagga	21	Gm07	-	tttcggaccaggcttcattcc	21	4453642	4453794	•	1		▴	3	
gma-miRC87	aatgtcgtctggttcgagacc	21	Gm02	+	tttcggaccaggcttcattcc	21	14340763	14340875	•	4		▴	0	
gma-miRC88	tttattgaaaatcacaaatta	21	Gm18	+	tttgtgattttcaataaatta	21	61878800	61878912	•	2	8	▴	1	4

E^a^(•): the miRNAs in ZP03-5373;re^b^: miRNA-5P reads of 5373 (RPM); re^c^: miRNA-3P reads of 5373 (RPM); E^d^(▴): the miRNAs in ZP03-5413;re^e^: miRNA-5P reads of 5413 (RPM);re^f^: miRNA-3P reads of 5413 (RPM). Empty apace means the same number with the previous row.

### Identification of the targets of miRNAs by degradome analysis

To identify the targets of the conserved and soybean-specific miRNAs reported here, we performed degradome sequencing to generate a total of 12.8 million short reads representing the 5′ ends of uncapped, poly-adenylated RNAs. About 77.66% of the unique reads were perfectly aligned to the soybean genome (Soybean Genome V9.0, http://www.phytozome.net/search.php). These reads were subsequently screened and analyzed using the Cleaveland 3.0 software [38]. A total of 42 targets in five categories (0 to 4) were identified (Table 3 and Figure S4), with 42 targets for 76 conserved and soybean-specific miRNAs belonging to 21 families (Table 3 and Figure S4).

**Table 3:** Identification of soybean miRNAs targets using the degradome.

miRNA	Target	Cs^a^	C^b^	P-value	Location	Target gene annotation
Targets for known miRNAs						
gma-miR1508a	Glyma16g27802.1	347	1	0.02	CDS	PPR superfamily protein
gma-miR1510a-3p	Glyma15g37255.2	743	0	0.01	CDS	TIR-NBS-LRR class
	Glyma15g37276.3	901	3	0.02	CDS	Auxin signaling F-box
gma-miR156c/d/e/i/j/l/m	Glyma04g32002.1	1937	0	0.02	3′-UTR	SBP dom ain containing protein
	Glyma11g36980.6	1243	0	0.01	CDS	SBP domain containing protein
	Glyma01g08056.1	1408	3	0.04	CDS	SBP domain containing protein
gma-miR156f	Glyma04g32002.1	1937	0	0.02	3′-UTR	SBP domain containing protein
	Glyma03g29901.1	1149	3	0.05	3′-UTR	SBP domain containing protein
	Glyma11g36980.6	1243	0	0.02	CDS	SBP domain containing protein
	Glyma18g36960.1	902	3	0.05	CDS	SBP domain containing protein
gma-miR156k/n/o	Glyma04g32002.1	1937	0	0.02	3′-UTR	SBP domain containing protein
	Glyma11g36980.6	1243	0	0.01	CDS	SBP domain containing protein
	Glyma01g08056.1	1408	3	0.05	CDS	SBP domain containing protein
gma-miR156p/r/t	Glyma04g32002.1	1937	0	0.02	3′-UTR	SBP domain containing protein
	Glyma11g36980.6	1243	0	0.01	CDS	SBP domain containing protein
	Glyma01g08056.1	1408	3	0.02	CDS	SBP domain containing protein
gma-miR164a/e/f/g/h/i/j/k	Glyma15g40510.1	734	2	0.01	CDS	NAC domain containing protein
gma-miR164b/c/d		734	2	0.02		NAC domain containing protein
gma-miR169o/r	Glyma07g01870.1	1328	1	0.03	3′-UTR	Flavonol synthase/flavanone 3-hydroxylase-like
gma-miR169p	Glyma03g36140.5	1569	3	0.02	3′-UTR	Nuclear transcription factor Y
	Glyma08g45030.1	1407	0	0.01	3′-UTR	Nuclear transcription factor Y
gma-miR171c/i-3p/o/q	Glyma06g11610.2	380	3	0.02	CDS	GRAS family transcription factor
gma-miR171k-3p	Glyma06g11610.2	380	3	0.04	CDS	GRAS family transcription factor
	Glyma13g02840.1	565	3	0.03	CDS	Nodulation-signaling pathway 2protein-like
gma-miR319a/b/e	Glyma08g10350.1	2130	3	0.04	3′-UTR	Transcription factor TCP2-like
	Glyma05g27367.3	2063	3	0.04	3′-UTR	Transcription factor TCP2-like
gma-miR319h/j/k/m	Glyma08g10350.1	2130	3	0.04	3′-UTR	Transcription factor TCP2-like
	Glyma05g27367.3	2063	3	0.04	3′-UTR	Transcription factor TCP2-like
gma-miR393a	Glyma02g17170.2	1741	0	0.01	CDS	F-box/RNI-like superfamily protein
gma-miR393c/d/e/f/g/	Glyma02g17170.2	1741	0	0.01	CDS	F-box/RNI-like superfamily protein
gma-miR393h/i/i/k	Glyma19g27280.1	2247	3	0.01	3′-UTR	Auxin signaling F-box
	Glyma02g43980.5	268	3	0.04	CDS	Ribosomal protein L20
gma-miR408a/b/c-3p	Glyma07g13840.1	885	0	0.00	3′-UTR	Stellacyanin-like
	Glyma04g42120.1	33	1	0.02	CDS	Plantacyanin
gma-miR4354	Glyma01g37690.2	402	1	0.01	CDS	Uncharacterized
gma-miR5770a	Glyma01g07860.1	235	1	0.03	CDS	Copper amine oxidase family protein
gma-miR5770b	Glyma01g07860.1	235	1	0.05	CDS	Copper amine oxidase family protein
Targets for conserved miRNA candidates						
gma-miR1510C1	Glyma02g08415.1	97	3	0.01	CDS	Uncharacterized
	Glyma16g27510.1	214	1	0.03	3′-UTR	Uncharacterized
gma-miR1514C1	Glyma07g01730.2	890	1	0.04	3′-UTR	Uncharacterized
gma-miR156C1	Glyma04g32002.1	1937	0	0.03	3′-UTR	SBP domain containing protein
	Glyma11g36980.6	1243	0	0.02	CDS	SBP domain containing protein
gma-miR156C2	Glyma04g32002.1	1937	0	0.03	3′-UTR	SBP domain containing protein
	Glyma11g36980.6	1243	0	0.02	CDS	SBP domain containing protein
gma-miR156C10	Glyma04g32002.1	1937	0	0.03	3′-UTR	SBP domain containing protein
	Glyma11g36980.6	1243	0	0.02	CDS	SBP domain containing protein
gma-miR157C1	Glyma17g18640.1	1974	3	0.02	3′-UTR	Integrase-type DNA-binding superfamily protein
	Glyma10g22390.2	1647	0	0.03	3′-UTR	ERF RAP2–7-like
gma-miR164C1	Glyma15g40510.1	734	2	0.01	CDS	NAC domain containing protein
gma-miR167C1	Glyma10g22390.2	1647	0	0.00	3′-UTR	ERF RAP2–7-like
gma-miR169C6	Glyma08g14700.1	227	1	0.02	CDS	Sulfate transporter
gma-miR171C3	Glyma08g10360.1	192	0	0.01	CDS	F-box family protein
	Glyma10g22790.2	201	0	0.00	CDS	F-box family protein
gma-miR172C3	Glyma14g37730.1	1307	3	0.01	CDS	UDP-Glycosyltransferase superfamily protein
gma-miR393C1	Glyma02g17170.2	1741	0	0.01	CDS	F-box/RNI-like superfamily protein
Targets for soybean-specific miRNA candidates						
gma-miRC15	Glyma01g08056.1	1409	3	0.04	CDS	SBP domain containing protein
	Glyma18g00903.1	2438	0	0.01	3′-UTR	SBP domain containing protein
	Glyma16g05895.2	1557	0	0.02	3′-UTR	SBP domain containing protein
	Glyma02g13371.2	1400	3	0.04	CDS	SBP domain containing protein
gma-miRC23	Glyma10g35481.1	1610	0	0.01	CDS	Auxin response factor
gma-miRC26	Glyma13g34690.2	953	3	0.04	CDS	Transcription factor TCP like
	Glyma08g10350.1	2130	3	0.04	3′-UTR	Transcription factor TCP2-like
	Glyma05g27367.3	2063	3	0.04	3′-UTR	Transcription factor TCP2-like
gma-miRC33	Glyma16g05500.1	2303	3	0.01	3′-UTR	Auxin signaling F-box
gma-miRC61	Glyma06g11610.2	380	3	0.03	CDS	GRAS family transcription factor
	Glyma01g38360.1	835	1	0.02	CDS	GRAS family transcription factor
gma-miRC83	Glyma07g13840.1	885	0	0.00	3′-UTR	Stellacyanin-like
	Glyma04g42120.1	33	1	0.05	CDS	Plantacyanin

a Cleavage site;^ b^Category 0: >1 raw read at the position, abundance at position is equal to the maximum on the transcript and there is only one maximum on the transcript. Category 1: >1 raw read at the position, abundance at position is equal to the maximum on the transcript, and there is more than one maximum position on the transcript. Category 2: >1 raw read at the position, abundance at position is less than the maximum but higher than the median for the transcript. Category 3: >1 raw read at the position, abundance at position is equal to or less than the median for the transcript. Category 4: only one raw read at the position. P-value should not exceed 0.05.

Among these targets for the conserved miRNA families, eight fell into category 0, which represented the most abundant degradome tags corresponding to the cleavage site and matching cognate transcripts, and one of them into category 2, whose cleavage abundance was higher than the median but below the maximum. The number of identified gene targets varied among the miRNAs, from one to four (Table 3). However, miRNAs that targeted members of a gene family usually had more targets. For example, miR156 could target four members of the squamosa promoter-binding-like protein family (Table 3). Although most of the genes (36 of 42) identified were the conserved targets of these miRNAs across a wide range of plant species, some (6 of 42) had not previously been reported in other species. For example, miR169, which is known to target NF-YA (nuclear factor-Y subunit alpha) in other species, was found to target the genes encoding flavonol synthase and sulfate transporter. Similarly, miR393, which exclusively targeted mRNAs for the F-box auxin receptors TIR1 (Transport Inhibitor Response Protein 1), and several members of auxin signaling F-box protein, the growth regulating factor (AFB) gene family in plants also targeted the ribosomal protein L20 gene (Table 3). It was noted that a few identified soybean-specific gene targets fell into category 4, a low-confidence group, and so should be further validated experimentally. Therefore, the targets falling into category 4 were not listed in the results.

Gene targets were also identified for six soybean-specific miRNAs. Of the 13 gene targets identified, 4 belonged to category 0 and 2 to category 1, while the remainder was classified into category 3 (Table 3). The soybean-specific miRNAs, like the conserved miRNAs, targeted genes of diverse functions. For example, gma-miRC23 targeted the gene encoding the auxin response factor, while gma-miRC33 targeted the gene encoding auxin-signaling F-box. Gma-miRC26 and gma-miRC61 each targeted members of gene families that encode the transcription factor TCP and the GRAS family transcription factor, respectively. Furthermore, gma-miRC15 targeted up to four members of the SBP-domain-containing-protein gene families. Hence, these soybean-specific miRNAs may be involved in the regulation of an array of metabolic and biological processes and signaling pathways.

### miRNAs triggered secondary siRNAs biogenesis pathway in soybean root

TAS transcripts are directed by miRNAs to produce tasiRNAs, which then guide the cleavage of other transcripts. To date, four TAS gene families have been characterized in *Arabidopsis*, of which the miR390-TAS3 and miR828-TAS4 pathways are conserved in plants [39], [40]. Here we identified TAS3 soybean orthologous genes (Glyma09g03731.1 and Glyma15g14675.1), together with their corresponding trigger miRNAs-miR390. These two genes also contained two complementary sites for gma-miR390, and the signatures were detected only at the 3′ target site (Figure S5). We also found similar siRNA biogenesis patterns in the cleaved TAS3 (Table S3). Together, these data indicate that miR390-TAS3 biogenesis pathways and functions are at least partially conserved in soybean root. Because auxin signaling and modulation are essential for diverse biological processes in soybean, especially root development and seed ripening [41], [42], miR390-TAS3 biogenesis-derived tasiARFs in roots could orchestrate auxin signaling that might be directly relevant to seed growth and development. In addition, four gma-miR393 target transcripts and three gma-miR1510 target transcripts in both ZP03-5373 and ZP03-5413 were identified as producing secondary siRNAs (Figure S5). Gma-miR393 and gma-miR1510–triggered secondary siRNA biogenesis pathways have been reported in soybean [15].

**Figure 3:**
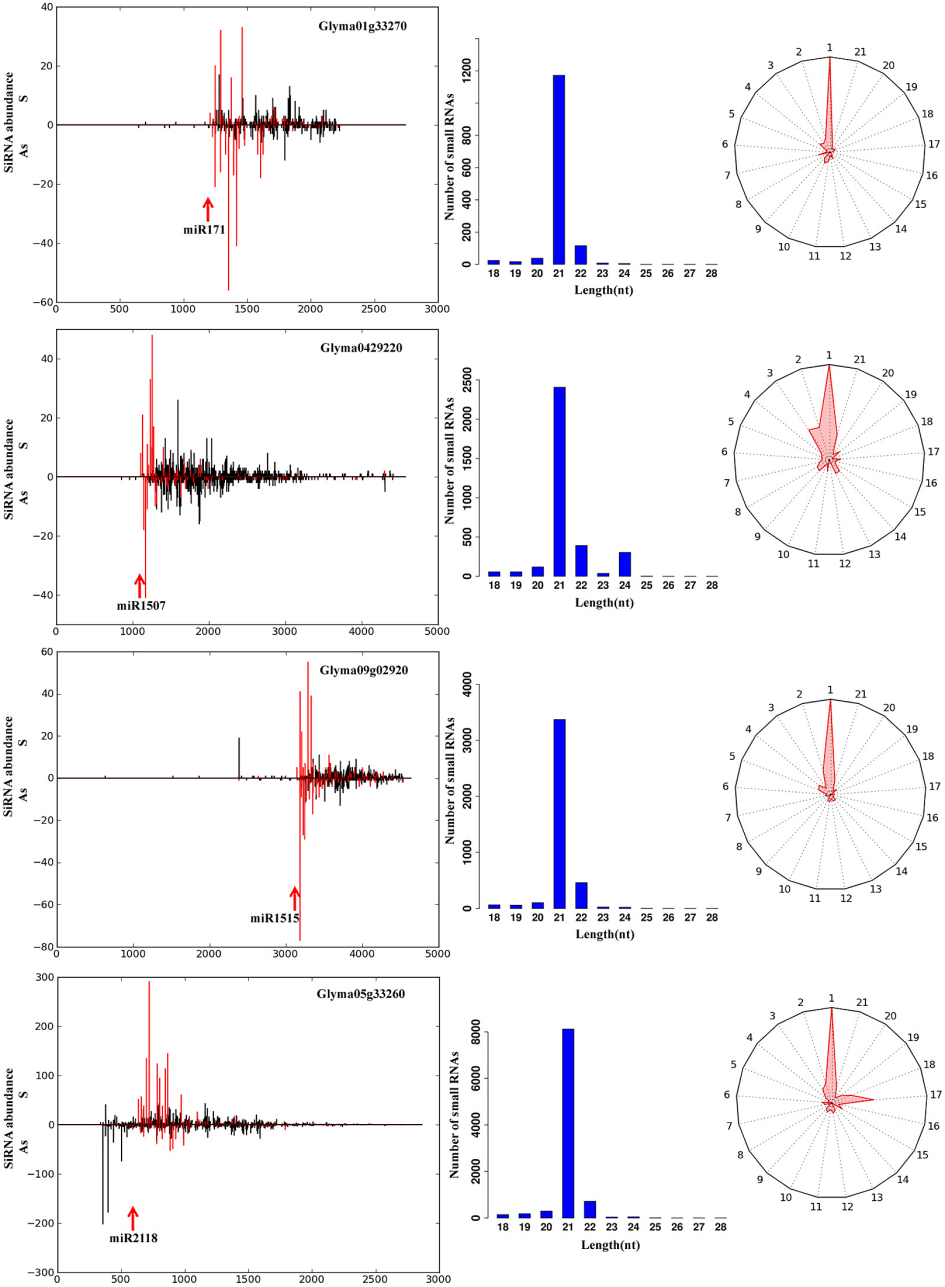
Five novel phasi-acting siRNA biogenesis pathways in soybean root. The abundance of each secondary siRNAs is plotted (left). The phasing secondary siRNAs corresponding to the miRNA cleavage sites are highlighted in red. The miRNA complementary sites are shown with red arrows. The length distribution is plotted on the right (middle). The phasing radial graph is represented next to this (right). Each spoke of the radial graph represents 1 of the 21 phasing registers, with the total number of sRNAs mapping to that register plotted as distance from the center. A, sense transcript; AS, antisense transcript.

The secondary small RNAs derived from all identified miRNA targets by PsRobot [16] in soybean were searched, and four transcripts (Glyma01g33270.1, Glyma04g29220.3, Glyma09g02920.2, Glyma05g33260.1), targeted respectively by gma-miR171, gma-miR1507, gma-miR1515, and gma-miR2118, were identified to produce secondary siRNAs (Figure 3).

The targets of these phasiRNAs were identified by analysis of the soybean degradome (Table S3). Besides the ARF4, a further five novel targets of miR390-TAS3 were found. Moreover, we identified six novel targets for the five phasiRNAs derived from gma-miR393 targets, eight novel targets for the five phasiRNAs derived from gma-miR1507 targets, 29 novel targets for the 22 phasiRNAs derived from gma-miR1510 targets, eight novel targets for the seven phasiRNAs derived from gma-miR1515 targets, five novel targets for the four phasiRNAs derived from gma-miR171 targets and 15 novel targets for the nine phasiRNAs derived from gma-miR2118 targets (Table S3).

### Verification of miRNA-guided cleavage of target mRNAs in soybean

**Figure 4:**
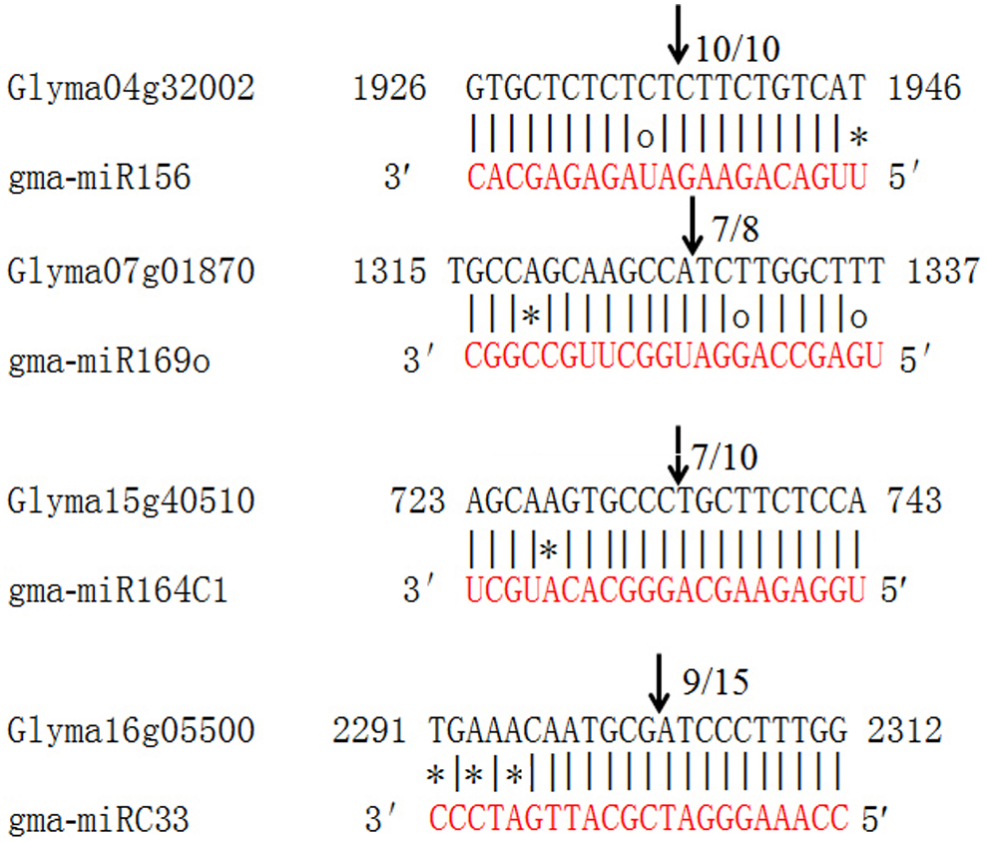
Differential expressed miRNAs in response to SCN.

To verify the miRNA-guided target cleavage, RLM-5′ RACE experiments were performed to detect cleavage products of the four predicted gma-miRNAs. As shown in Figure 4, all four gma-miRNA guided target cleavages occurred at nucleotide 10 or 11 (Figure 4). Thus, all four predicted targets had specific cleavage sites corresponding to the miRNA complementary sequences.

### SCN-infection-associated miRNAs

**Figure 5:**
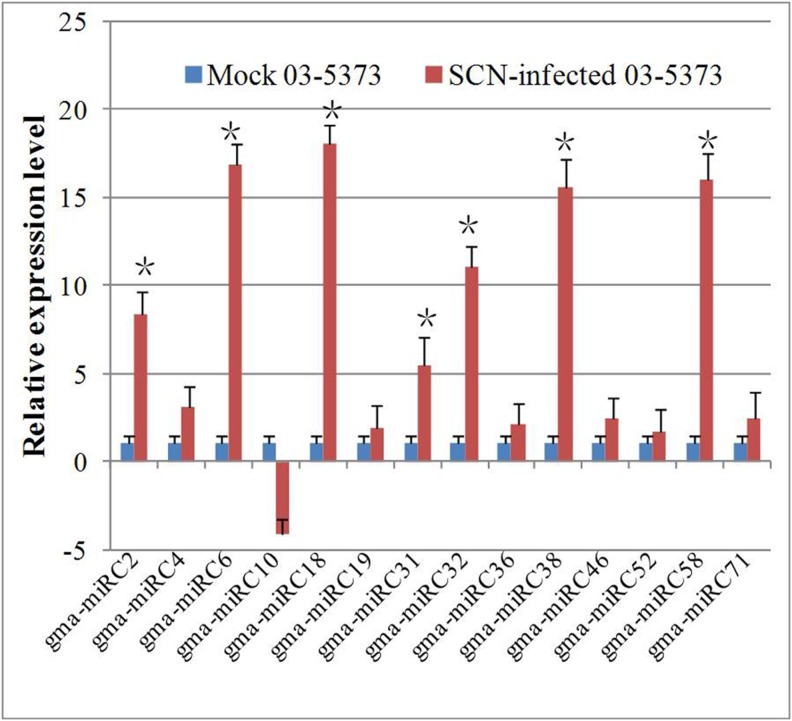
Predicted schematic model of miRNA-SCN system in soybean root.

The sequencing frequencies for miRNAs in the two libraries were used as an index for estimation of the relative abundance. The expression levels in SCN-resistant soybean root and SCN-sensitive soybean root were compared based on the “reads per million” genome-matched reads (RPM) of miRNAs. Using ZP03-5373 (RPM)/ZP03-5413 (RPM) values >5 or <5, a total of 34 miRNAs belonging to 27 families were identified to be significantly differentially expressed. The results are shown in Table S4. Most of the differentially expressed miRNAs were up regulated in the roots of the SCN-resistant line ZP03-5373 (Table S4). Although the absolute expression level of miRNA is useful, the identification of differential expression profiles at the whole-genome level in response to endogenous cues or stresses is often desirable to detect miRNA function in particular cell processes. In order to examine if the miRNAs might play a role in SCN resistance, the expression pattern of 14 miRNAs that were expressed specifically in ZP03-5373 were analysed using qRT-PCR in SCN-infected and uninfected ZP03-5373 plants. Seven miRNAs were up regulated significantly after the SCN-infection (Figure 5), and therefore appeared to be important in SCN infection and re-generation. A search of the SCN genome sequences identified 44 potential target genes in SCN by these seven SCN-inducible soybean miRNAs, suggesting a possible function of these miRNAs in regulating the expression of these SCN genes (Table 4).

**Table 4:** Potential targets in SCN for miRNAs expressed at a high level in the ZP03-5373 line.

miRNAs	Target gene^a^	E-value^b^	Annotation
gma-miRC6	gi|10713756	9.00E-28	Hypothetical protein WUBG_11282,partial
	gi|10713770	2.00E-08	Hypothetical protein CBG14284
	gi|10713984	2.00E-08	Hypothetical protein CBG14284
	gi|10713995	2.00E-08	Hypothetical protein CBG14284
	gi|10714125	2.00E-08	Hypothetical protein CBG14284
	gi|10713899	3.00E-47	Translocon-associated proteinsubunit beta(SSR2)
	gi|10713915	4.00E-25	transcription regulator NC2 alphachain
	gi|10713976	3.00E-13	FLP-16 protein
	gi|10714333	3.00E-13	FLP-16 protein
	gi|10714022	2.00E-09	CBN-ATP-4 protein
	gi|10714052	4.00E-16	acyl carrier protein (ACP)
	gi|10714121	1.00E-66	Protein HSP-25, isoform
	gi|10714146	5.00E-16	Protein VHA-14
	gi|10714164	8.00E-14	Protein NEF1
	gi|10714245	2.00E-66	Protein mago nashi homolog(MAGOH)
	gi|10714275	9.00E-13	hypothetical proteinDAPPUDRAFT_330564
	gi|10714325	5.00E-08	Patched domain-containingprotein 3 (PTCHD3) homolog
	gi|10714342	2.00E-12	hypothetical protein Bm1_39195
gma-miRC6/miRC46	gi|10714166	1.00E-12	Ribosomal protein L39(RPL39)
gma-miRC18	gi|10713732	1.00E-26	hypothetical proteinCAEBREN_03276
	gi|10713748	6.00E-77	Ubiquitin- Conjugating Enzyme(Ubc-2)
	gi|10713868	4.00E-49	Hypothetical protein CBG12012
	gi|10713929	6.00E-08	Immediate early response3-interacting protein 1(IER3IP1)
	gi|10713932	2.00E-14	RE18871p
	gi|10713953	3.00E-21	conserved hypothetical proteinDUF1242
	gi|10714016	1.00E-08	39 S ribosomal protein L32
	gi|10714199	6.00E-59	hypothetical proteinCAEBREN_23803
	gi|10714274	3.00E-11	Protein ATP-4
gma-miRC18/miRC38	gi|10714087	3.00E-06	hypothetical proteinLOAG_04475
gma-miRC31	gi|10713799	2.00E-54	40 S ribosomal protein S18
	gi|10714049	2.00E-54	40 S ribosomal protein S18
gma-miRC32	gi|10713767	3.00E-27	cleavage stimulation factorsubunit 2
	gi|10714061	3.00E-27	cleavage stimulation factorsubunit 2
	gi|10713949	7.00E-11	Import inner membranetranslocase subunit tim-13
	gi|10713988	1.00E-82	troponin C-like protein
	gi|10714106	3.00E-05	hypothetical proteinLOAG_03714
	gi|10714297	1.00E-25	NADH dehydrogenaseubiquinone 1 alpha subcomplexsubunit2 (NDUFA2)
	gi|10714305	9.00E-20	cytochrome P450, family 3,subfamily A, polypeptide 5
gma-miRC32/miRC58	gi|10714119	1.00E-39	Eukaryotic translation initiationfactor 1A, Y-chromosomal
gma-miRC58	gi|10714213	3.00E-06	Protein MICAL-3
	gi|10713990	3.00E-06	Protein MICAL-3
	gi|10714084	3.00E-06	Protein MICAL-3
	gi|10714090	3.00E-06	Protein MICAL-3
	gi|10714167	3.00E-11	Transcriptional activator proteinPur-alpha

a The target gene is the transcript identified from the SCN ESTs. (http://www.ncbi.nlm.nih.gov/Taxonomy/Browser/wwwtax.cgi?id=51029); b E-value was calculated according to Blast and should be less than 1.00E-5.

Forty-four transcripts were predicted to be potential targets of differentially expressed miRNAs. A large number of the identified targets were function proteins (Table 4), including NADH dehydrogenase, SSR2, FLP and CBN-ATP-4; *i.e.*, the relative expression level of gma-miRC6 in ZP03-5373 was markedly higher than that of ZP03-5413 (Table 2 and figure 5). Nineteen targets of gma-miRC6 were identified. One of the gma-miRC6 targets, the SSR2 gene, encodes a translocon-associated protein subunit beta, which is associated with protein translocation across the endoplasmic reticulum (ER) membrane. Another miRC6-targeted FLP-16 protein was potentially involved in the neuropeptide signaling pathway and negatively regulated striated muscle contraction. The acyl carrier protein (ACP) is an important component in the fatty acid synthase complex. MAGOH mutations in the mago nashi (grandchildless) gene produce progeny with defects in germplasm assembly and germline development [43], [44]. PTCHD3 plays a role in reproduction development [45], while others are related to protein phosphorylation (AGC family protein kinase) and ATPase activity (Protein VHA-14). All of these genes are important in the development and regeneration of SCN. Ten genes were potential targets of gma-miRC18, which is involved in both the folding and transportation of proteins, and degradation pathways (Table 4). The gma-miRC32 targets encode a hypothetical NADH dehydrogenase, which is the first enzyme required in the respiratory chain pathway.

## Discussion

Soybean is an important economic crop. Recently, high-throughput sequencing of sRNAs and RNA degradome has been successfully used to reveal large numbers of soybean miRNAs and their targets. A number of miRNAs have been reported to be involved in organ development [46], nutrient signaling [47], biotic and abiotic stress [48], [49]. These studies imply the important roles of miRNAs in soybean development and interaction with environment, which provide clues for deciphering the functions for microRNA/target modules in soybean. SCN is a significant plant pathogen responsible for an estimated $2 billion annually in yield losses worldwide. The planting of resistant soybean cultivars is the key to managing SCN population levels in the field. Despite some resistant cultivars having been developed and used, there remains a lack of understanding of the molecular basis of the resistance to this pathogen because only two major loci, rhg1 and rhg4 have been cloned [50]; the remaining quantitative trait loci (QTLs) are distributed on the other 16 linkage groups (LG) (except LG D1b and F) (soybase.net) and remain unknown. Progress in understanding the effectiveness and durability of natural plant resistance and enabling the design of novel strategies for resistance through biotechnological approaches has, therefore, been limited. Comparison of the gene expression profiles of soybean–SCN interactions has revealed distinct differences in gene expression between the resistant and susceptible reactions. Therefore, it is important to select suitable soybean lines to detect differently expressed genes.

In this study, to develop a better understanding of the molecular events associated with resistance to SCN race 4, we employed the sister lines, ZP03-5373 and ZP03-5413, in a comparative analysis of sRNA expression using deep sequencing. ZP03-5373 and ZP03-5413 have similar agronomic traits except for resistance to SCN race 4. Our previous study showed that ZP03-5373 was resistant but ZP03-5413 was susceptible to SCN 4, suggesting that some differentially expressed genes may have negative impacts on syncytium development and maintenance.

SCNs are highly evolved sedentary plant endoparasites that can enter soybean roots to successfully parasitize plants. RNA interference (RNAi) involving host-induced gene silencing in parasites has been reported [5]. A potential mechanism underlying the involvement of miRNAs in controlling cyst nematodes is proposed. Here, the candidate targets of differentially expressed miRNAs in SCN were predicted (Table 4). Our results predicted the existence of a novel miRNA-mediated regulatory cascade involved in the SCN life cycle in soybean root. These observations demonstrate the relevance of the targeted genes of SCN during the nematode life cycle and, potentially more importantly, suggest that an effective resistance to cyst nematodes in soybean may be achieved using this technology. But which should be confirmed by experiment in the future.

## Conclusions

This study describes large scale cloning and characterization of two genetically related soybean sister lines miRNAs, phasiRNAs and their potential targets, we also found that the expression of 34 miRNAs differed significantly between the two lines. Seven ZP03-5373-specific miRNAs were differentially expressed after SCN infection. Forty-four transcripts from SCN were predicted to be potential targets of ZP03-5373-specific differential miRNAs. These findings suggest that miRNAs play an important role in the soybean response to SCN and providing the foundation for further characterization of their roles in the regulation of diverse physiological processes.

## Methods

### Plant materials

Two genetically related soybean lines, Zhongpin03-5373 (ZP03-5373) and Zhongpin03-5413 (ZP03-5413), which are resistant and susceptible, respectively, to SCN race 4 were used in this study. The two sister lines, ZP03-5373 and ZP03-5413 were developed from the cross of two SCN resistant parents “Jin 1265” ⋅ “Hartwig”. The former was resistant and the latter was susceptible to SCN race 4. Elite line Jin 1265 was derived from cultivar Hupizhi Heidou for its resistance. Thus, ZP03-5373 and ZP03-5413 have the same genetically pedigrees but different resistance to SCN race 4, which provided an opportunity to gain further insight into the underlying genetic control of resistance. Soybean were grown in a glasshouse at 22–25°C with a 16 h light/8 h dark photoperiod and light intensity of >8000 lx. Roots from 3-weeks-old seedlings were collected and used for RNA extraction. And was used for small RNA expression and degradome analysis.

### RNA extraction and preparation of sRNA and degradome cDNA libraries for Solexa sequencing

Soybean root total RNA was extracted using TRIzol (Invitrogen, Carlsbad, CA, USA). Total RNA was size-fractionated by 15% denaturing polyacrylamide gel electrophoresis, after which small RNA fragments of 18–28 nt were isolated from the gel and purified. The small RNA molecules were then sequentially ligated to a 5′ adaptor and a 3′ adaptor and converted to cDNA by RT-PCR following the Illumina protocol. The concentration of the sample was adjusted to ∼10 nM and a total of 10 µL were used in a sequencing reaction. The purified cDNA library was sequenced on an Illumina GAIIx.

The degradome library was constructed as described previously [33]. For the short RNA libraries, the degradome cDNA library was sequenced on an Illumina GAIIx.

### Bioinformatic analyses

After masking adaptor sequences and removal of contaminated reads, the clean reads were filtered for miRNA prediction. First, reads that matched rRNA, tRNA, snRNA, snoRNA, repeat sequences, and other ncRNAs deposited in Rfam (http://www.sanger.ac.uk/software/Rfam) [51] and the GenBank noncoding RNA database (http://www.ncbi.nlm.nih.gov/) were discarded. The retained 18–28-nt reads were mapped onto the genome of soybean, using V 9.0 (http://www.phytozome.net) by the bowtie2 software. All perfectly matched sRNAs were retained for miRNA prediction. After rigorous screening, all retained sequences of 18–28 nt with a frequency of three or more copies were considered potential miRNAs. We then attempted to align the predicted miRNAs to all soybean known mature miRNA sequences in miRBase, version 19.0 [51] to identify novelty. Finally, secondary structure prediction of individual miRNAs was performed with the MFOLD software (Version 2.38, http://mfold.rna.albany.edu/?q=mfold/RNA-Folding-Form) using the default folding conditions [52], and novel miRNAs were predicted using the psRobot software [53]. The identification of phased transcripts in soybean was performed by a method described previously [54].

The degradome analysis and the classification of target categories were performed using CleaveLand 3.0 [38]. Small RNA target prediction was run against the transcriptome of interest. The alignment scores (using the [8] rubric) for each hit up to a user-defined cutoff were calculated, full RNA-RNA alignments were printed, and the ‘cleavage site’ associated with each prediction was also calculated. The cleavage site is simply the 10^th^ nt of complementarity to the aligned sRNA. For randomized queries, no alignments were retained; however, concise records of each predicted target for the random queries were retained, including the predicted cleavage sites. We also used the psRobot software to identity the targets of phasiRNAs. miRNA targets were predicted in SCN using the microTar software (http://tiger.dbs.nus.edu.sg/microtar/) [55].

### End-point and SYBR Green I real-time PCR assays of soybean miRNAs

End-point and real-time looped RT-PCR [56] were used to validate and measure the levels of soybean miRNA. Stem–loop RT primers, a universal reverse primer and miRNA-specific forward primers for gma-miR160a, gma-miR398a, gma-miR398c, gma-miR399a, gma-miR399d, gma-miR4412–5p, gma-miR862a, gma-miR156C1, gma-miR160C1, gma-miR157C5, gma-miR159C1, gma-miR172C1, gma-miR2109C1, gma-miR393C1, gma-miR395C1, gma-miR399C3, gma-miR399C5, gma-miR399C7, gma-miR1C2, gma-miRC4, gma-miRC6, gma-miRC10, gma-miRC18, gma-miRC19, gma-miRC31, gma-miRC32, gma-miRC36, gma-miRC38, gma-miRC46, gma-miRC49, gma-miRC52, gma-miRC58, gma-miRC71 and gma-miRC75 were designed according to Varkonyi-Gasic *et al*.[48] (Additional file 10: Table S5). One microgram of total RNA was reverse-transcribed to cDNA using ReverTra Ace (TOYOBO, Osaka, Japan). Stem-loop pulsed reverse transcription and end-point PCR were performed according to [56]. Real time qRT-PCR (quantitative reverse transcriptase PCR) was performed using SYBR Premix Ex Taq™ of TaKaRa (TaKaRa Code: DRR041A) on a model 7500 thermocycler (Applied Biosystems, Foster City, CA, USA). All reactions were run in triplicate. After the reaction, the threshold cycle (Ct) was determined using default threshold settings. The Ct was defined as the fractional cycle number at which the fluorescence surpasses the fixed threshold.

## Supporting Information

Figure S1 **Secondary structures of 71 putative less-conserved soybean miRNAs and miRNAs.** Pink section represents miRNA-5p; yellow section represents miRNA-3p.
(PDF)

Figure S2 **qRT-PCR results.** qRT-PCR confirming express pattern of miRNAs in ZP03-5373 and ZP03-5413. The expression levels were normalized against the U6 RNA.
(JPG)

Figure S3 **Secondary structures of 75 putative soybean-specific miRNAs and miRNAs counterparts.** Pink section represents miRNA-5p; yellow section represents miRNA-3p.
(DOCX)

Figure S4 **degradome T-plot.** We used reads in plotting the cleavages on target mRNAs, which were referred to as ‘target plots’ (t-plots) by German et al [17]. Signature abundance throughout the length of the indicated transcripts is shown. miRNA:mRNA alignments along with the detected cleavage frequencies are shown. The frequencies of degradome tags with 5′ends at the indicated positions are shown in black, with the frequency at position 10 of the inset miRNA target alignment highlighted in red.
(PDF)

Figure S5 **The small RNAs corresponding to the miRNA targets.** The abundance of each secondary siRNAs is plotted (A). The phasing secondary siRNAs corresponding to the miRNA cleavage sites are highlighted in red. The miRNA complementary sites are shown with red arrows. The length distribution is plotted on the right (B). The phasing radial graph is represented next to this (C). Each spoke of the radial graph represents 1 of the 21 phasing registers, with the total number of sRNAs mapping to that register plotted as distance from the center. A, sense transcript; AS, antisense transcript.
(PDF)

Table S1 **Statistics of sRNA sequences from** ***G. max.*** a: Redundancy(%) = 100-(Total unique high quality Reads/Total high quality Reads x 100); b: using soap2.0 aligner;c: Glycine max Genome were downlaod from Phytozome (http://www.phytozome.net/index.php),and the version is 9.0.
(XLSX)

Table S2 **Known miRNAs identified in** ***G. max.***
(XLSX)

Table S3 **miRNAs triggered secondary phasiRNAs and its targets.**
(XLS)

Table S4 **Differential expressed soybean miRNAs.**
(XLS)

Table S5 **miRNA and primer sequences.**
(XLSX)
