# Supplementary material for: Novel MiRNA and PhasiRNA Biogenesis Networks in Soybean Roots from Two Sister Lines That Are Resistant and Susceptible to SCN Race 4
Source: PLoS One. 2014 Oct 30;9(10):e110051. doi: 10.1371/journal.pone.0110051 (PMC4214822; doi:10.1371/journal.pone.0110051)
Supplement: Figure S1 — Secondary structures of 71 putative less-conserved soybean miRNAs and miRNAs. Pink section represents miRNA-5p; yellow section represents miRNA-3p. (PDF) [file pone.0110051.s001.pdf]

Figure S1 Secondary structures of 71 putative less-conserved soybean miRNAs and miRNAs*

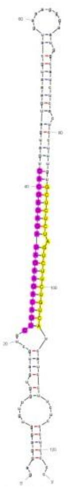	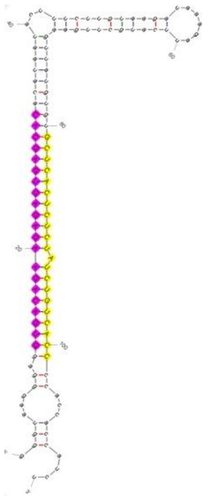	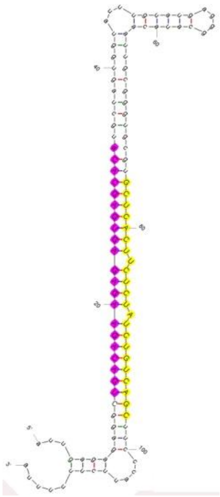	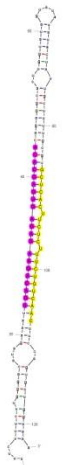	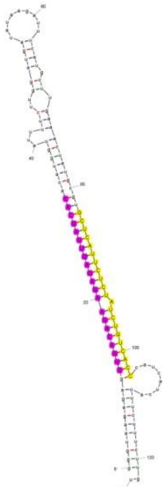
gma-miR156C1	gma-miR156C2	gma-miR156C3-C4	gma-miR156C5-C6	gma-miR156C7-C8
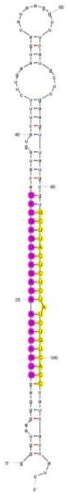	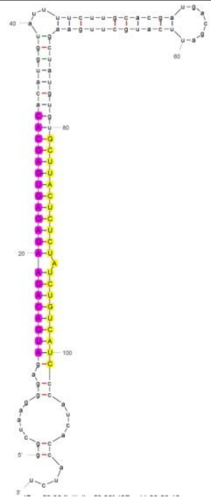	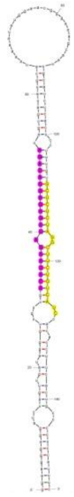	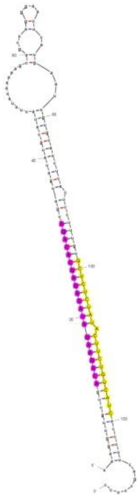	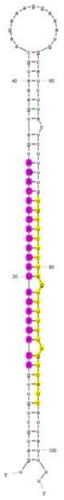
gma-miR156C9	gma-miR156C10	gma-miR157C1-C2	gma-miR157C3	gma-miR157C4
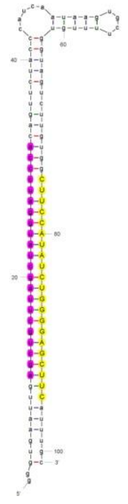	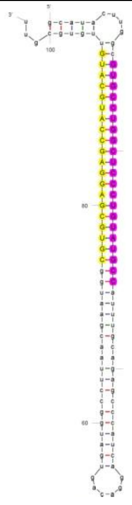	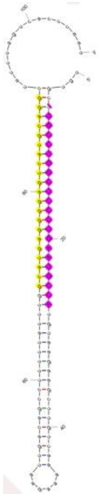	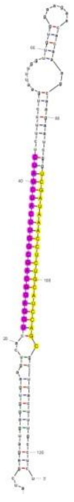	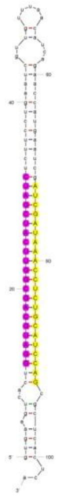
gma-miR159C1-C2	gma-miR160C1	gma-miR160C2	gma-miR162C1-C2	gma-miR162C3

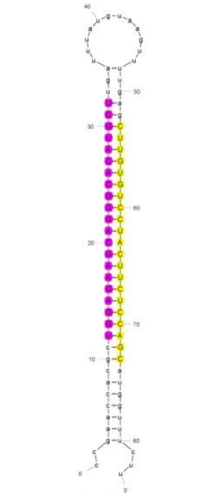	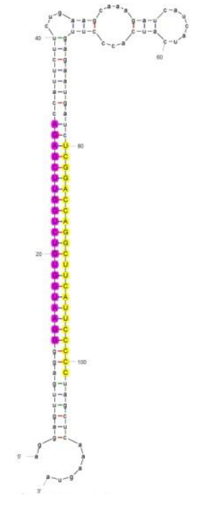	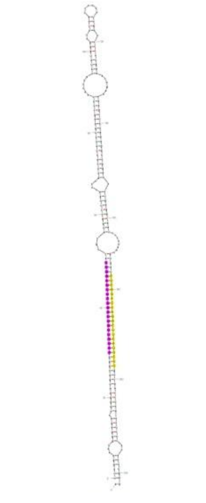	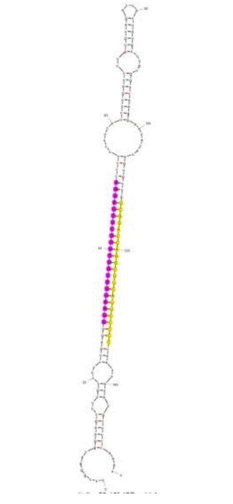	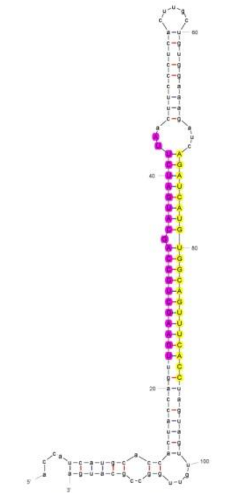
gma-miR164C1	gma-miR166C1-C2	gma-miR166C3	gma-miR166C4	gma-miR167C1-C2
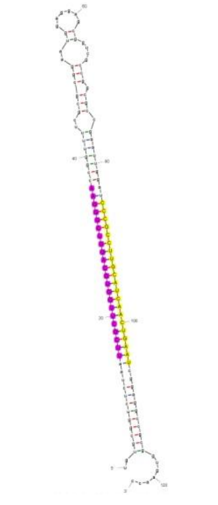	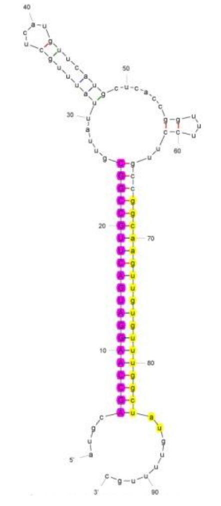	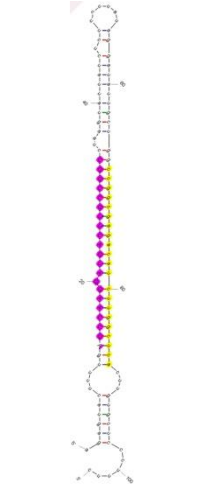	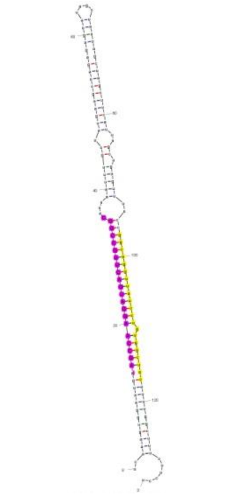	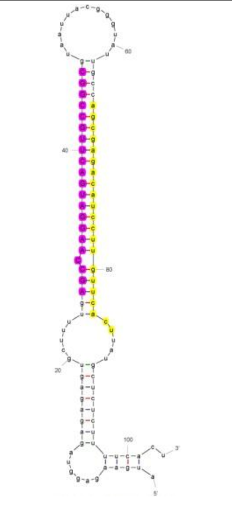
gma-miR168C1-C2	gma-miR169C1-C2	gma-miR169C3-C4	gma-miR169C5	gma-miR169C6
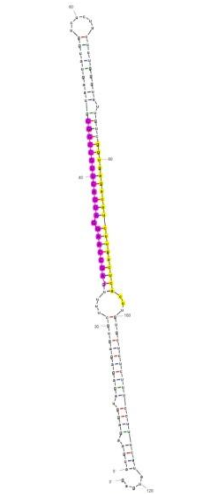	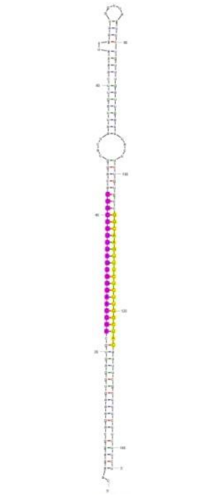	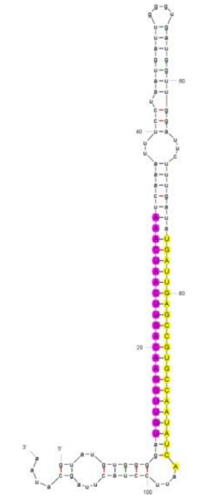	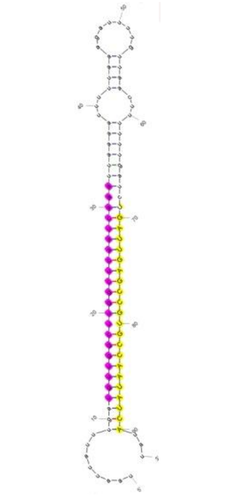	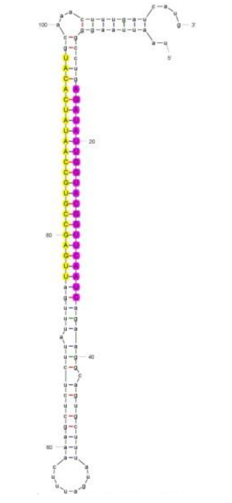
gma-miR169C7	gma-miR169C8	gma-miR71C1	gma-miR171C2	gma-miR171C3-C4

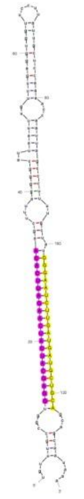	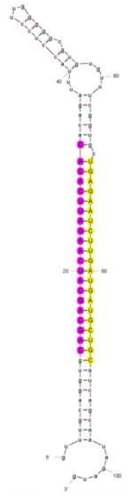	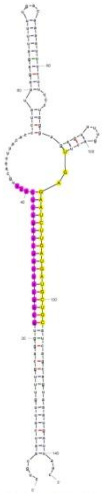	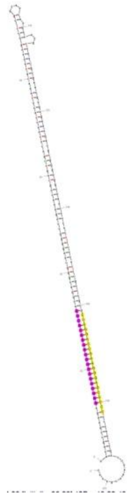	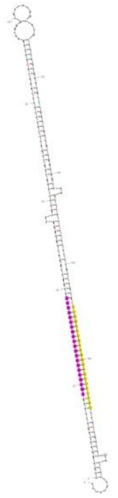
gma-miR172C1-C2	gma-miR172C3-C4	gma-miR172C5	gma-miR319C1-C2	gma-miR319C3-C4
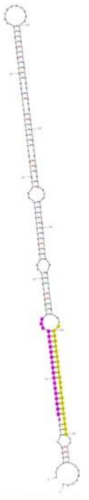	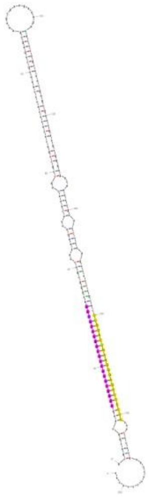	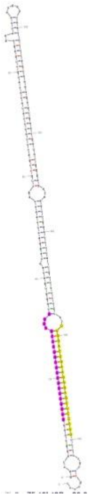	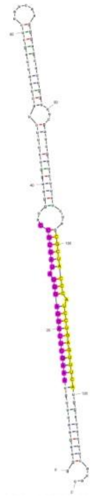	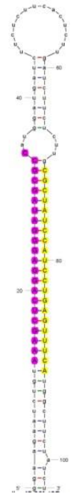
gma-miR319C5	gma-miR319C6	gma-miR319C7	gma-miR390C1	gma-miR390C2-C3
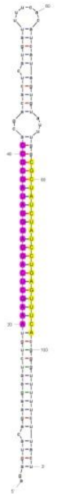	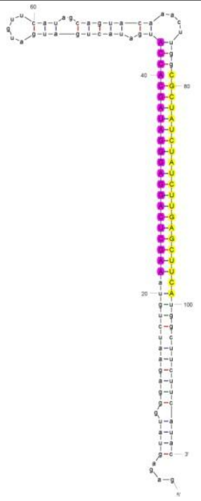	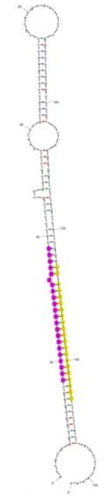	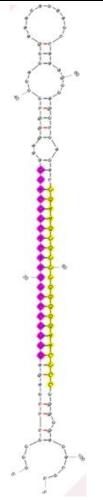	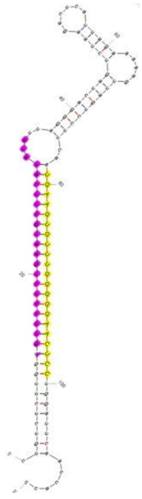
gma-miR390C4-C5	gma-miR390C6	gma-miR393C1	gma-miR395C1	gma-miR395C2

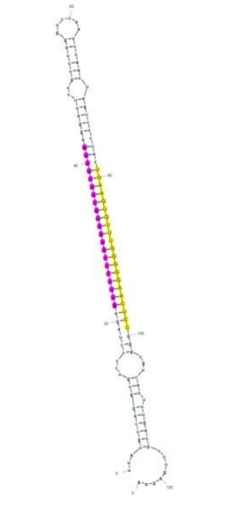	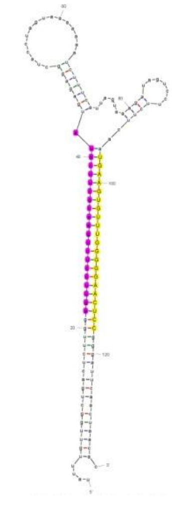	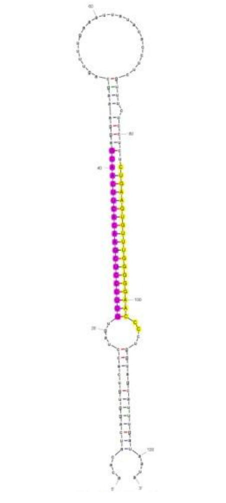	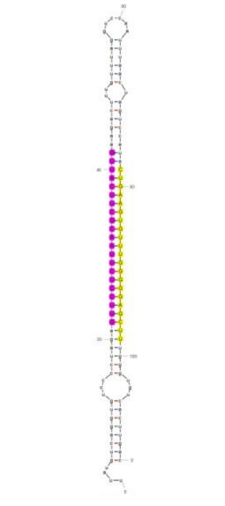	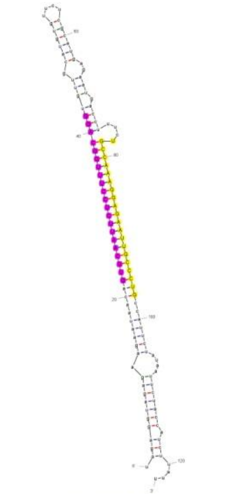
gma-miR395C3	gma-miR395C4	gma-miR395C5	gma-miR395C6	gma-miR399C1
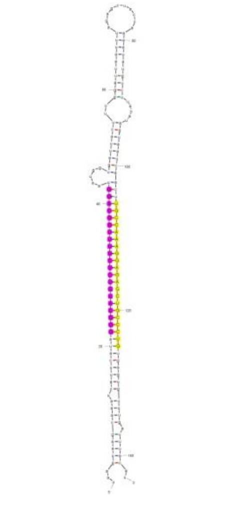	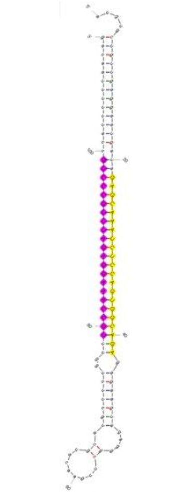	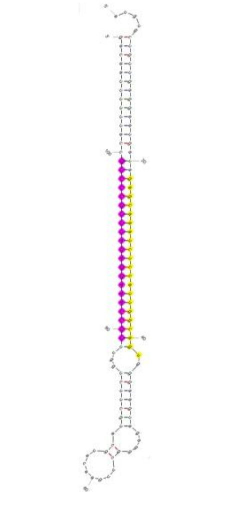	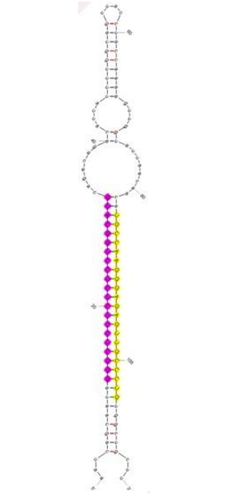	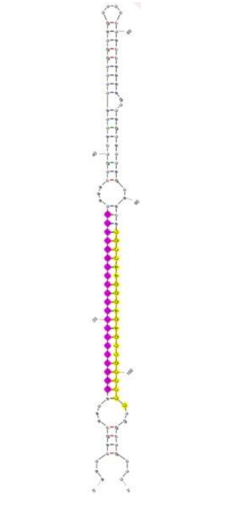
gma-miR399C2	gma-miR399C3	gma-miR399C4	gma-miR399C5	gma-miR399C6
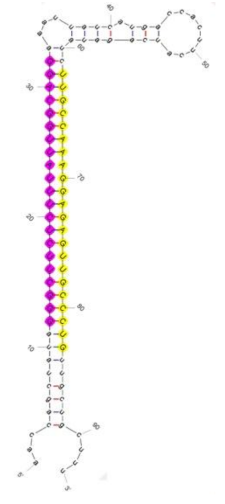	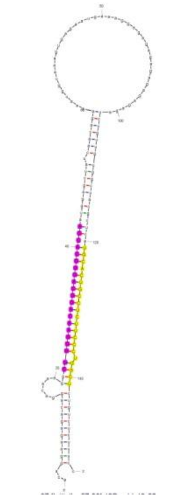	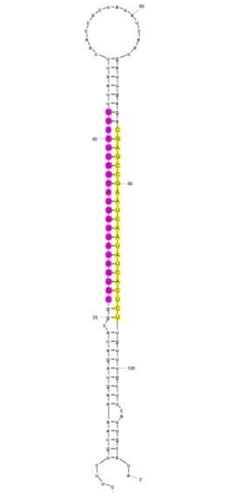	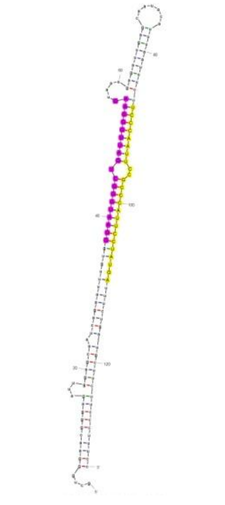	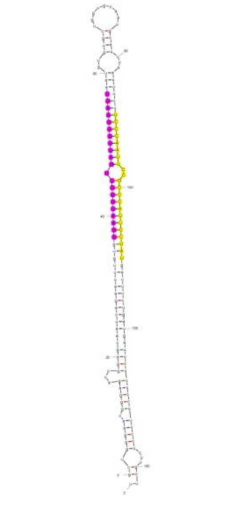
gma-miR399C7-C8	gma-miR408C1	gma-miR479C1-C2	gma-miR482C1	gma-miR482C2

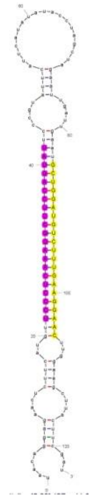	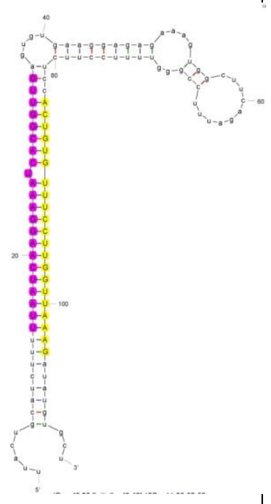	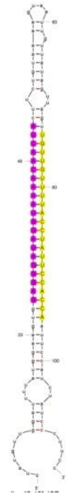	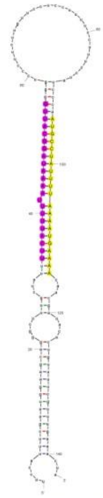	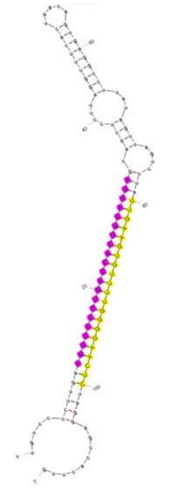
gma-miR862C1	gma-miR1509C1	gma-miR1510C1	gma-miR1514C1	gma-miR1514C2
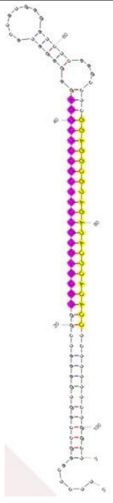	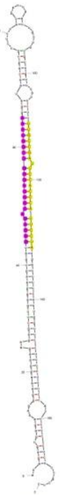	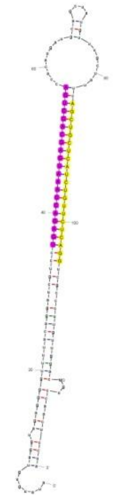	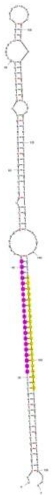	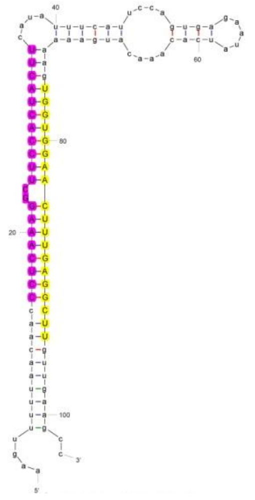
gma-miR2109C1	gma-miR2118C1	gma-miR3522C1	gma-miR4416C1	gma-miR5037C1
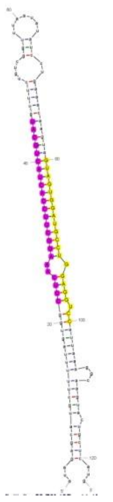				
gma-miR5044C1				
