# Supplementary figures and images for: Novel MiRNA and PhasiRNA Biogenesis Networks in Soybean Roots from Two Sister Lines That Are Resistant and Susceptible to SCN Race 4
Source: PLoS One. 2014 Oct 30;9(10):e110051. doi: 10.1371/journal.pone.0110051 (PMC4214822; doi:10.1371/journal.pone.0110051)

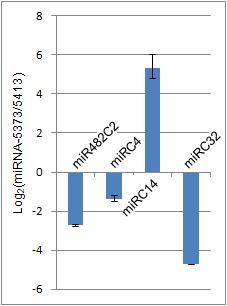

Supplement: Figure S2 — qRT-PCR results. qRT-PCR confirming express pattern of miRNAs in ZP03-5373 and ZP03-5413. The expression levels were normalized against the U6 RNA. (JPG) [file pone.0110051.s002.jpg]
