# Supplementary material for: Novel MiRNA and PhasiRNA Biogenesis Networks in Soybean Roots from Two Sister Lines That Are Resistant and Susceptible to SCN Race 4
Source: PLoS One. 2014 Oct 30;9(10):e110051. doi: 10.1371/journal.pone.0110051 (PMC4214822; doi:10.1371/journal.pone.0110051)
Supplement: Figure S3 — Secondary structures of 75 putative soybean-specific miRNAs and miRNAs counterparts. Pink section represents miRNA-5p; yellow section represents miRNA-3p. (DOCX) [file pone.0110051.s003.docx]

| 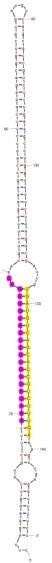 | 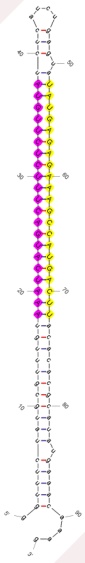 | 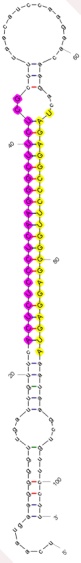 | 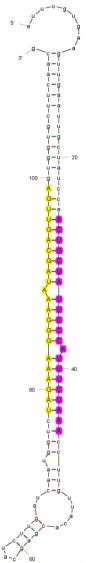 | 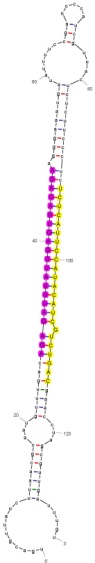 |
| --- | --- | --- | --- | --- |
| gma-miRC1 | gma-miRC2 | gma-miRC3 | gma-miRC4 | gma-miRC5 |
| 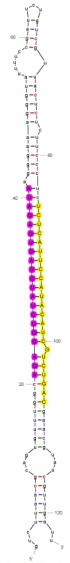 | 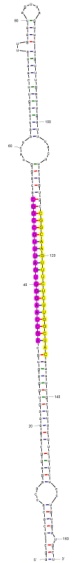 | 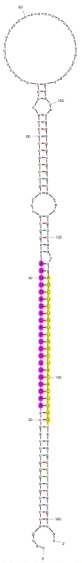 | 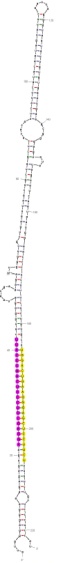 | 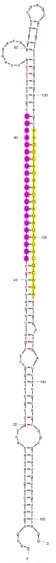 |
| gma-miRC6 | gma-miR6C7 | gma-miRC8 | gma-miR C9 | gma-miR C10 |
| 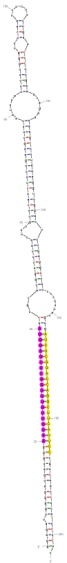 | 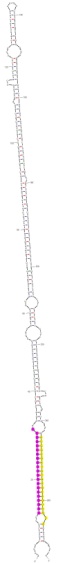 | 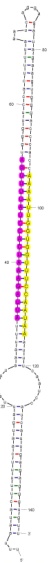 | 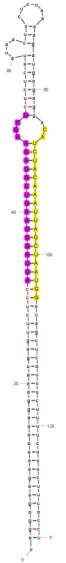 | 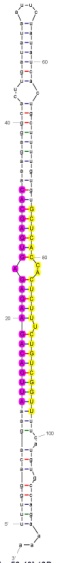 |
| gma-miR C11 | gma-miR C12 | gma-miR C13 | gma-miRC14 | gma-miR C15 |

Figure S3 Secondary structures of 75 putative soybean-specific miRNAs and miRNAs* counterparts

| 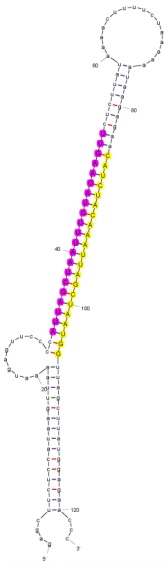 | 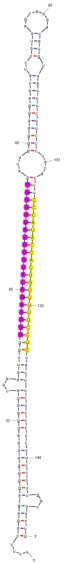 | 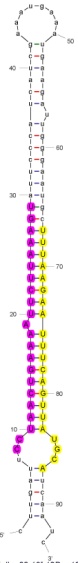 | 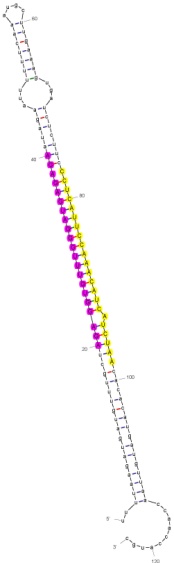 | 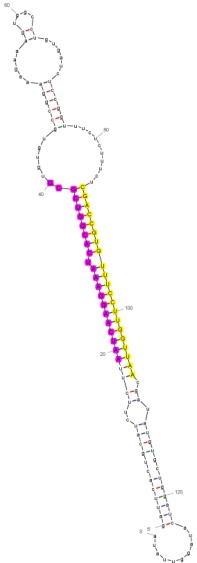 |
| --- | --- | --- | --- | --- |
| gma-miRC16-C17 | gma-miRC18 | gma-miRC19 | gma-miRC20 | gma-miRC21 |
| 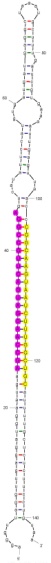 | 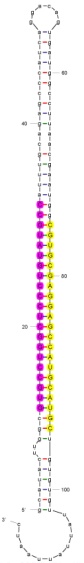 | 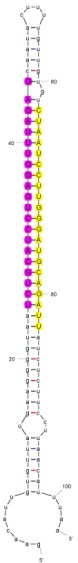 | 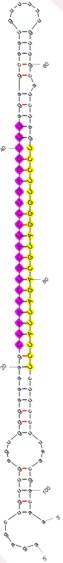 | 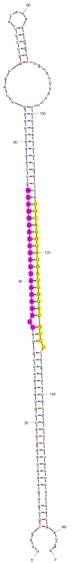 |
| gma-miRC22 | gma-miRC23 | gma-miRC24 | gma-miRC25 | gma-miRC26 |
| 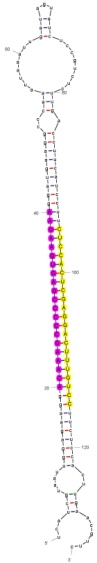 | 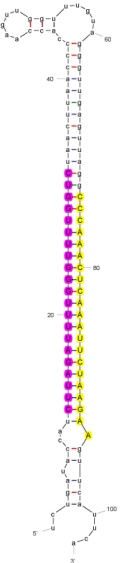 | 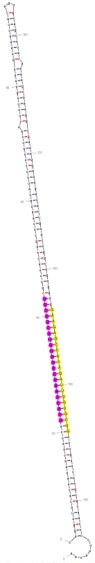 | 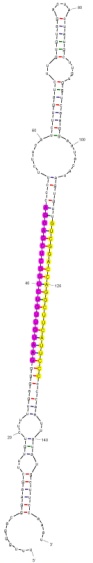 | 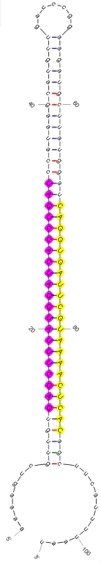 |
| gma-miRC27 | gma-miRC28 | gma-miRC29 | gma-miRC30 | gma-miRC31 |

| 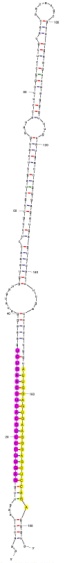 | 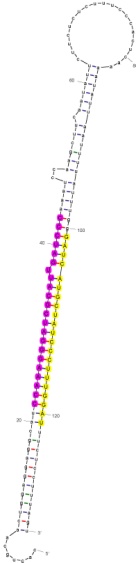 | 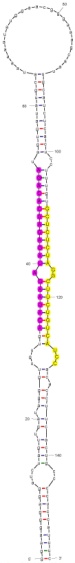 | 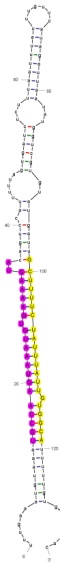 | 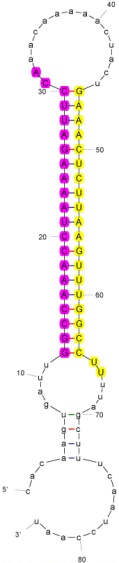 |
| --- | --- | --- | --- | --- |
| gma-miRC32 | gma-miRC33-C34-C35 | gma-miRC36 | gma-miRC37 | gma-miRC38 |
| 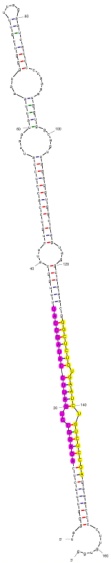 | 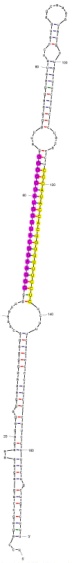 | 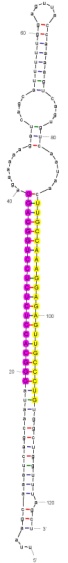 | 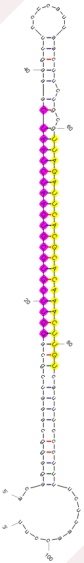 | 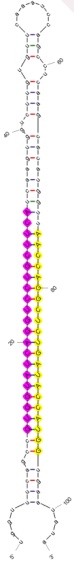 |
| gma-miRC39 | gma-miRC40 | gma-miRC41-C42 | gma-miRC43 | gma-miRC44 |
| 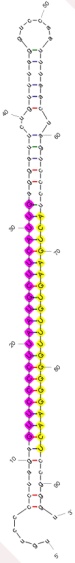 | 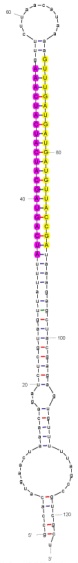 | 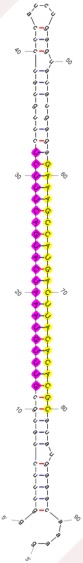 | 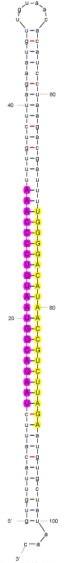 | 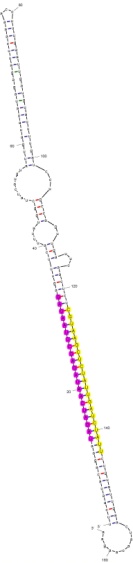 |
| gma-miRC45 | gma-miRC46- C47- C48 | gma-miRC49 | gma-miRC50 | gma-miRC51 |

| 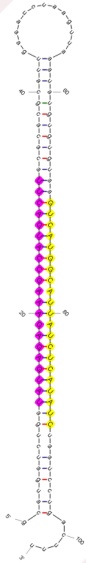 | 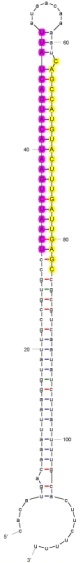 | 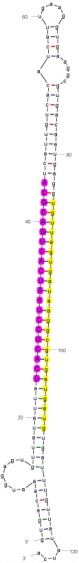 | 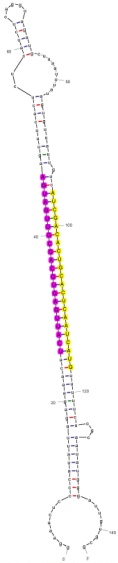 | 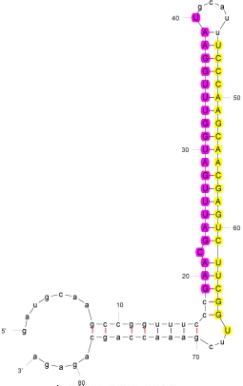 |
| --- | --- | --- | --- | --- |
| gma-miRC52 | gma-miRC53 | gma-miRC54 | gma-miRC55 | gma-miRC56-C57 |
| 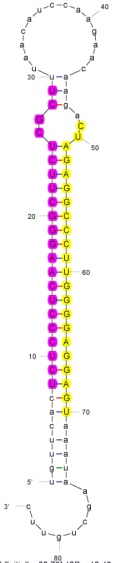 | 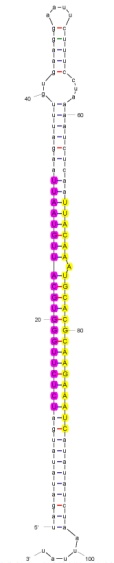 | 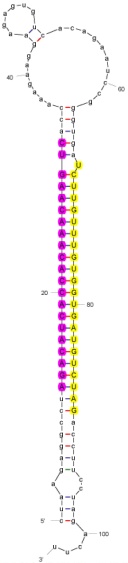 | 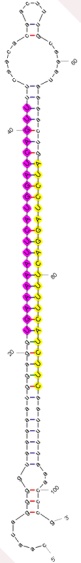 | 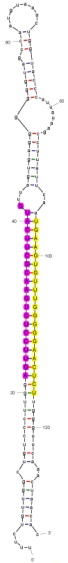 |
| gma-miRC58 | gma-miRC59 | gma-miRC60 | gma-miRC61 | gma-miRC62- C65 |
| 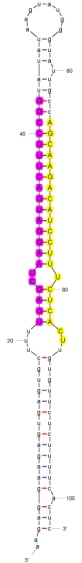 | 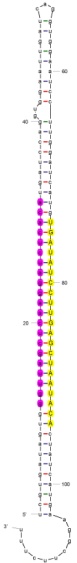 | 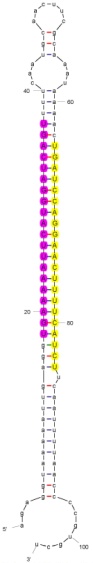 | 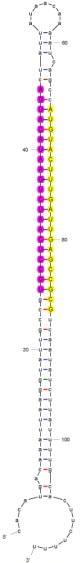 | 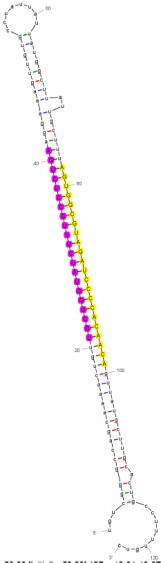 |
| gma-miRC66 | gma-miRC67 | gma-miRC68 | gma-miRC69 | gma-miRC70 |

| 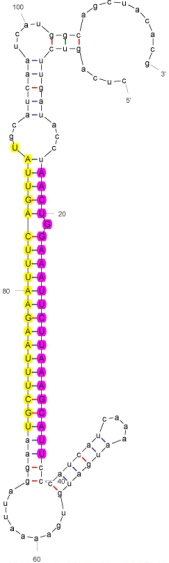 | 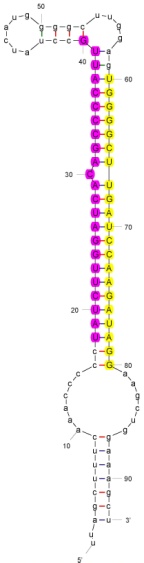 | 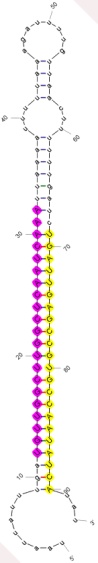 | 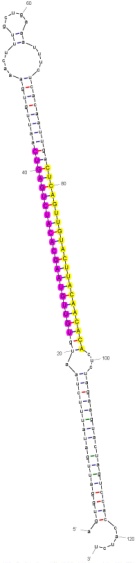 | 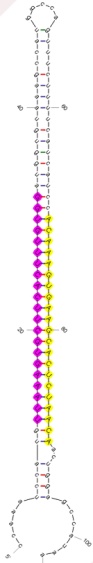 |
| --- | --- | --- | --- | --- |
| gma-miRC71 | gma-miR7C72 | gma-miRC73 | gma-miRC74 | gma-miRC75 |
| 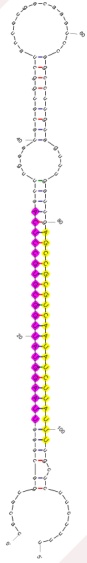 | 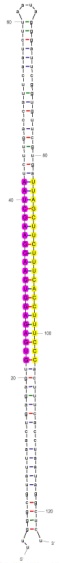 | 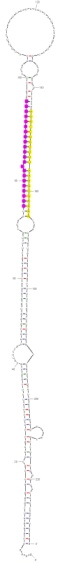 | 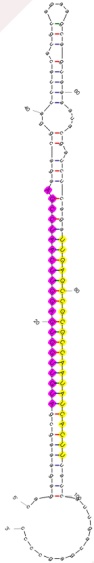 | 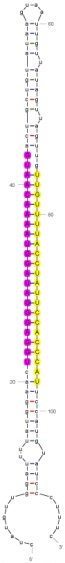 |
| gma-miRC76 | gma-miR7C77 | gma-miRC78 | gma-miRC79- C80 | gma-miRC81 |
| 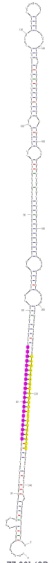 | 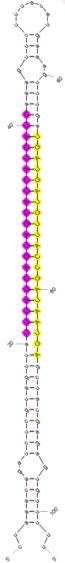 | 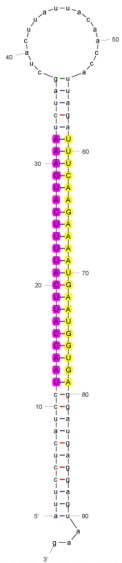 | 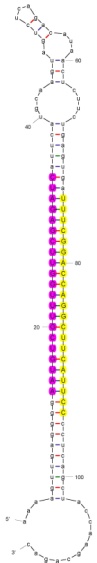 | 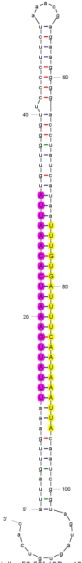 |
| gma-miRC82 | gma-miR7C83 | gma-miRC84 | gma-miRC85-C87 | gma-miRC88 |
