# Supplementary material for: Novel MiRNA and PhasiRNA Biogenesis Networks in Soybean Roots from Two Sister Lines That Are Resistant and Susceptible to SCN Race 4
Source: PLoS One. 2014 Oct 30;9(10):e110051. doi: 10.1371/journal.pone.0110051 (PMC4214822; doi:10.1371/journal.pone.0110051)
Supplement: Figure S4 — degradome T-plot. We used reads in plotting the cleavages on target mRNAs, which were referred to as ‘target plots’ (t-plots) by German et al [17]. Signature abundance throughout the length of the indicated transcripts is shown. miRNA:mRNA alignments along with the detected cleavage frequencies are shown. The frequencies of degradome tags with 5′ends at the indicated positions are shown in black, with the frequency at position 10 of the inset miRNA target alignment highlighted in red. (PDF) [file pone.0110051.s004.pdf]

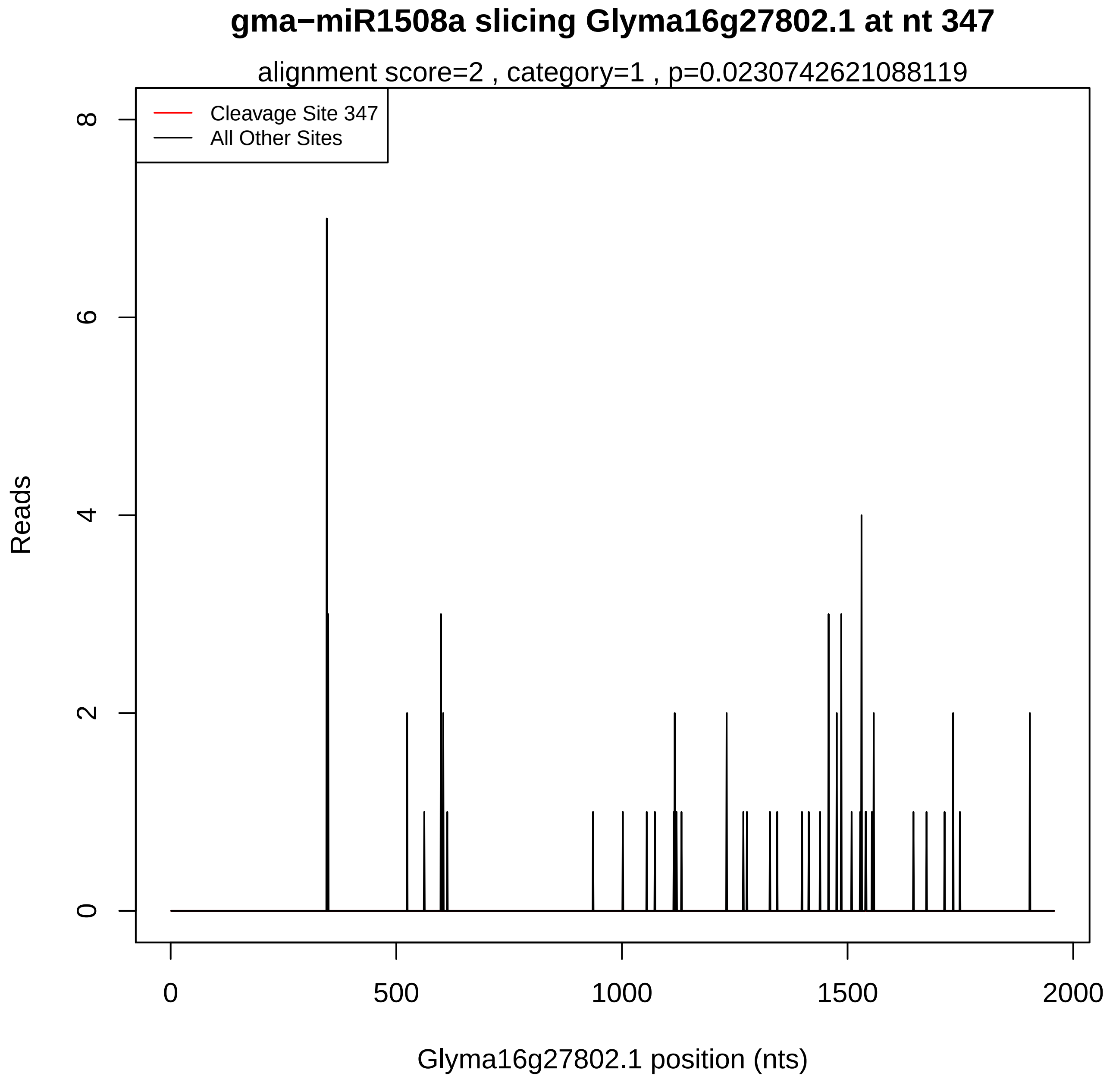
gma−miR1508a slicing Glyma16g27802.1 at nt 347
alignment score=2 , category=1 , p=0.0230742621088119
Cleavage Site 347
All Other Sites
Reads
0
2
4
6
8
0
500
1000
1500
2000
Glyma16g27802.1 position (nts)

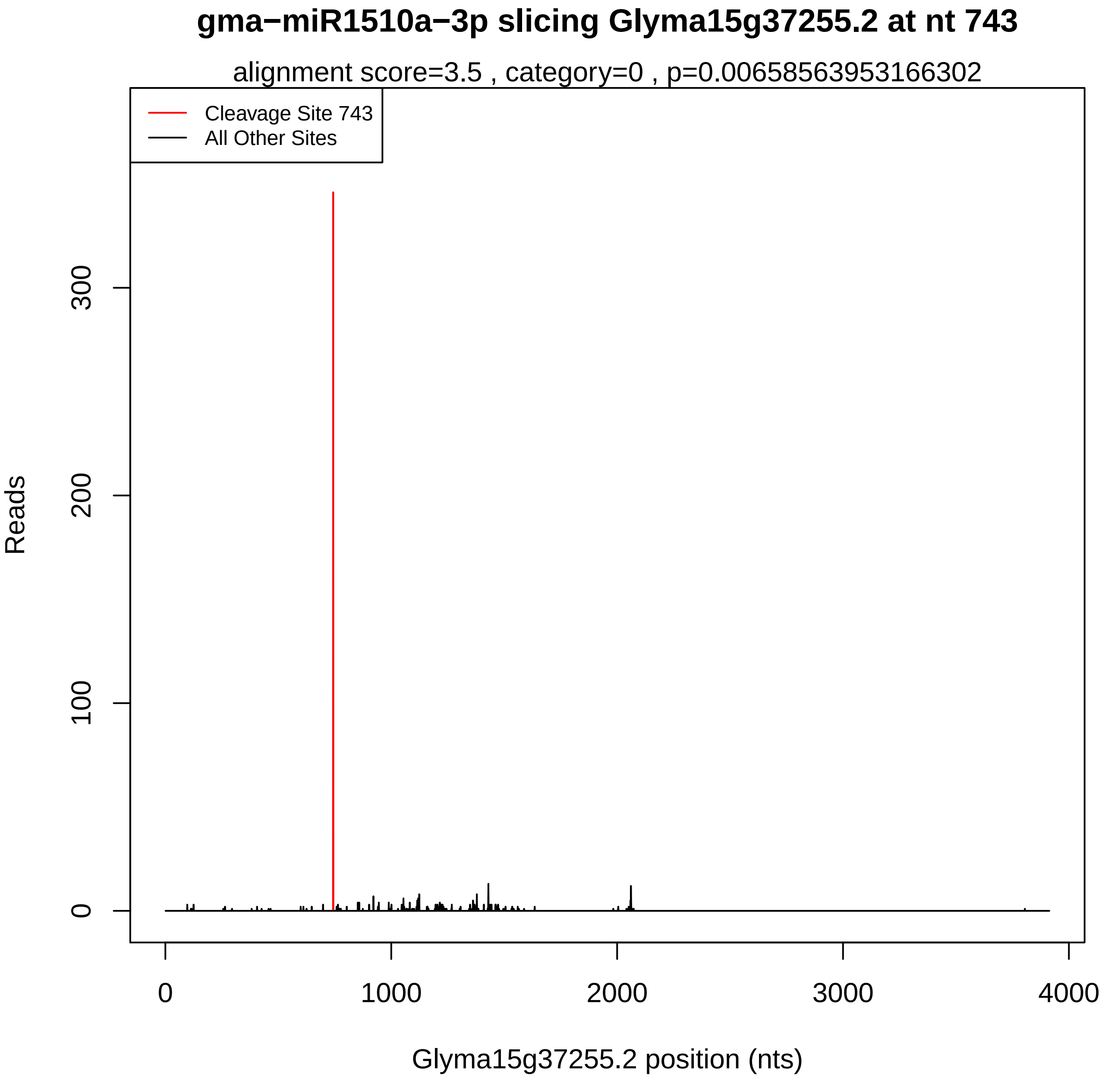
gma−miR1510a−3p slicing Glyma15g37255.2 at nt 743
alignment score=3.5 , category=0 , p=0.00658563953166302
Cleavage Site 743
All Other Sites
Reads
0
100
200
300
0
1000
2000
3000
4000
Glyma15g37255.2 position (nts)

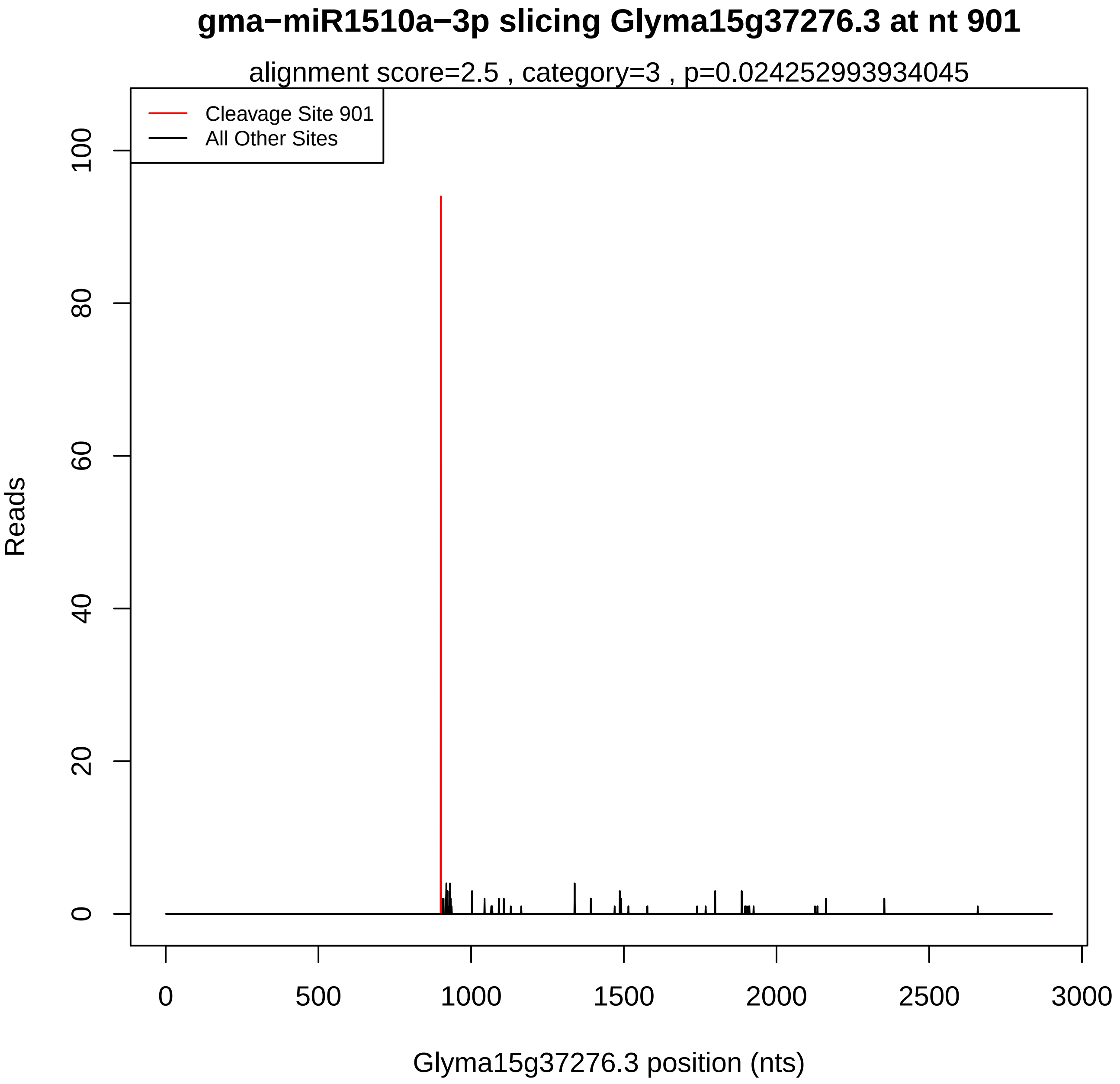

gma−miR1510a−3p slicing Glyma15g37276.3 at nt 901
alignment score=2.5 , category=3 , p=0.024252993934045
Cleavage Site 901
All Other Sites
Reads
0
20
40
60
80
100
0
500
1000
1500
2000
2500
3000
Glyma15g37276.3 position (nts)

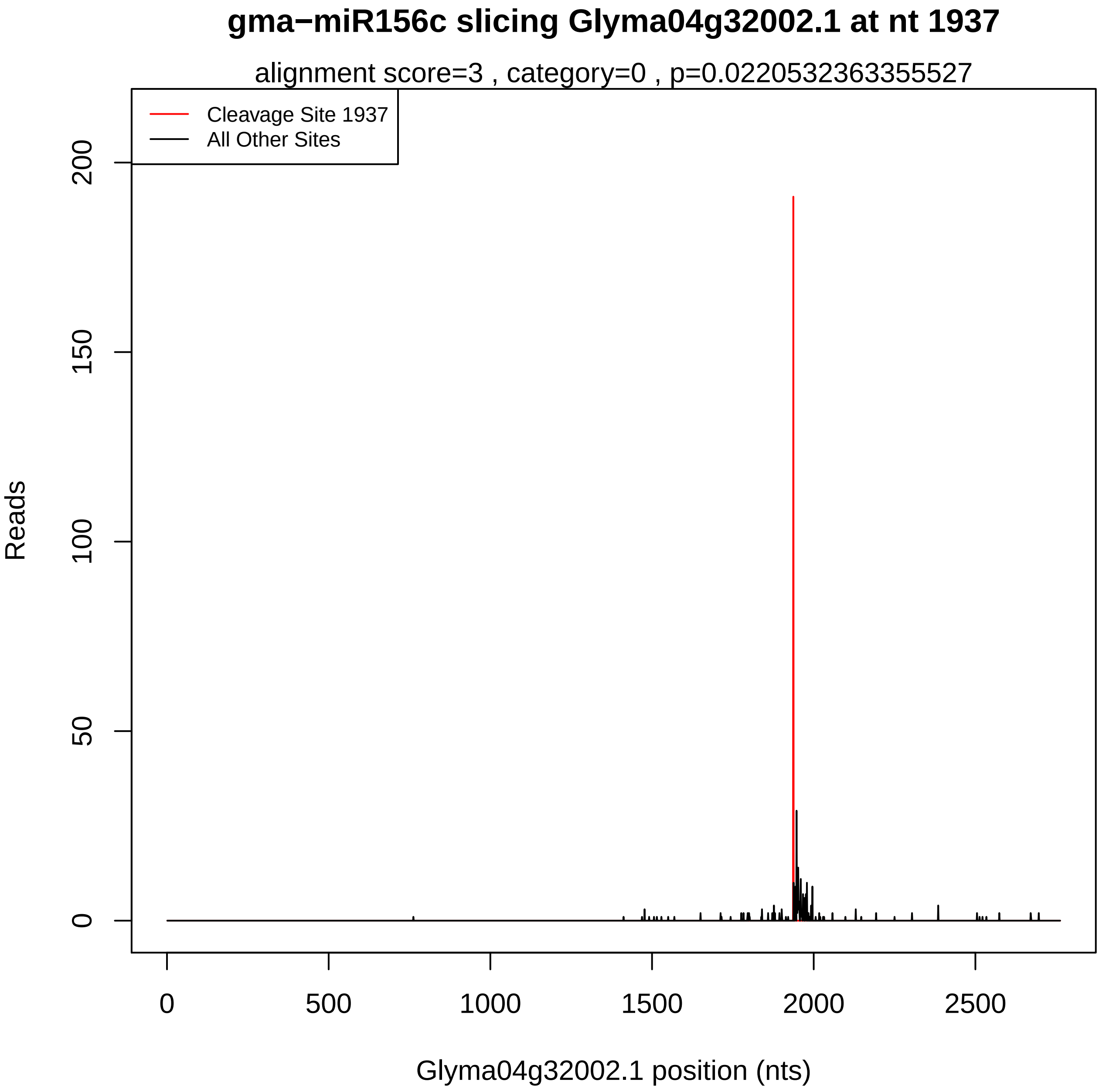
gma−miR156c slicing Glyma04g32002.1 at nt 1937
alignment score=3 , category=0 , p=0.0220532363355527
Cleavage Site 1937
All Other Sites
Reads
0
50
100
150
200
0
500
1000
1500
2000
2500
Glyma04g32002.1 position (nts)

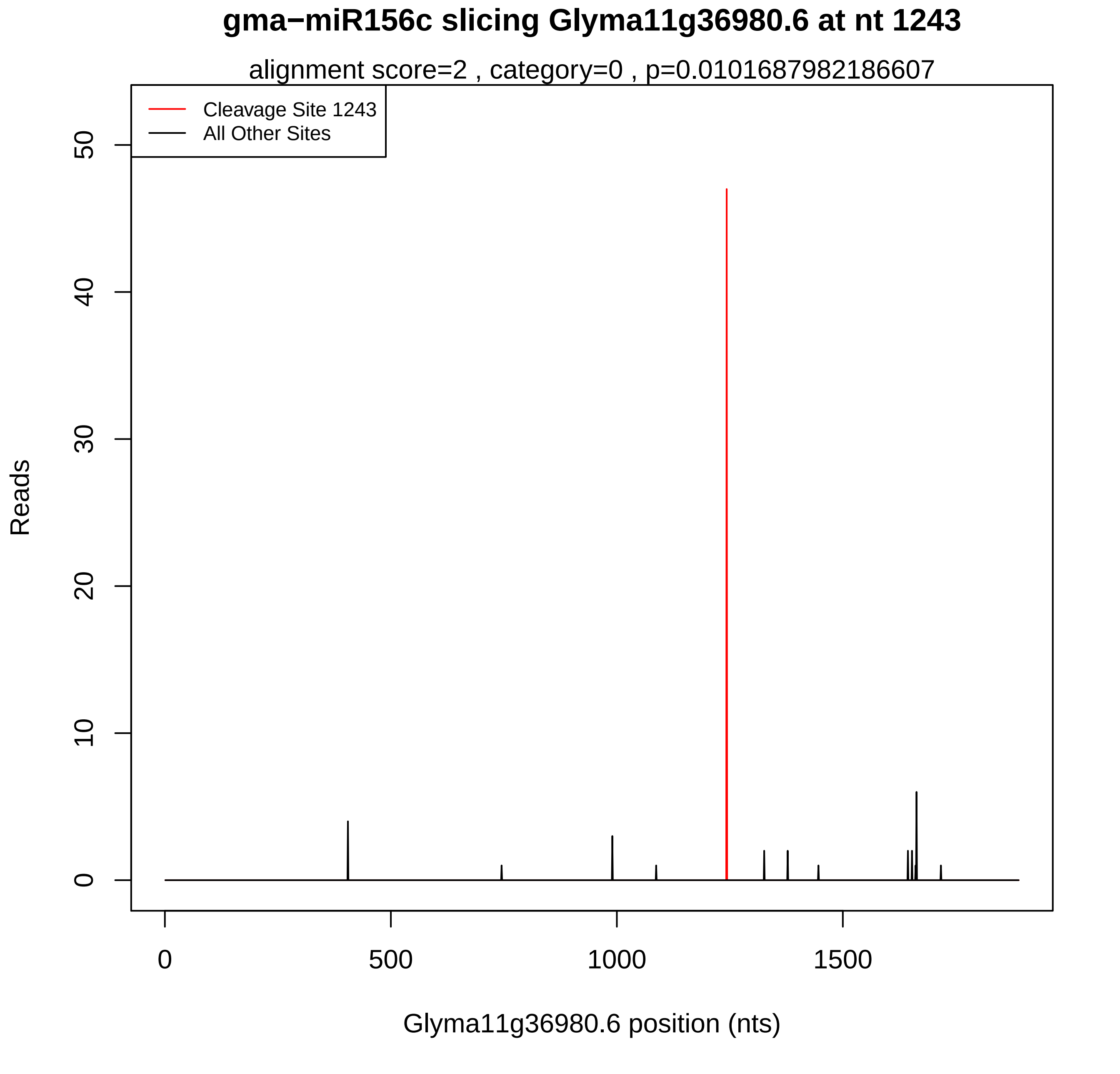
gma−miR156c slicing Glyma11g36980.6 at nt 1243
alignment score=2 , category=0 , p=0.0101687982186607
Cleavage Site 1243
All Other Sites
Reads
0
10
20
30
40
50
0
500
1000
1500
Glyma11g36980.6 position (nts)

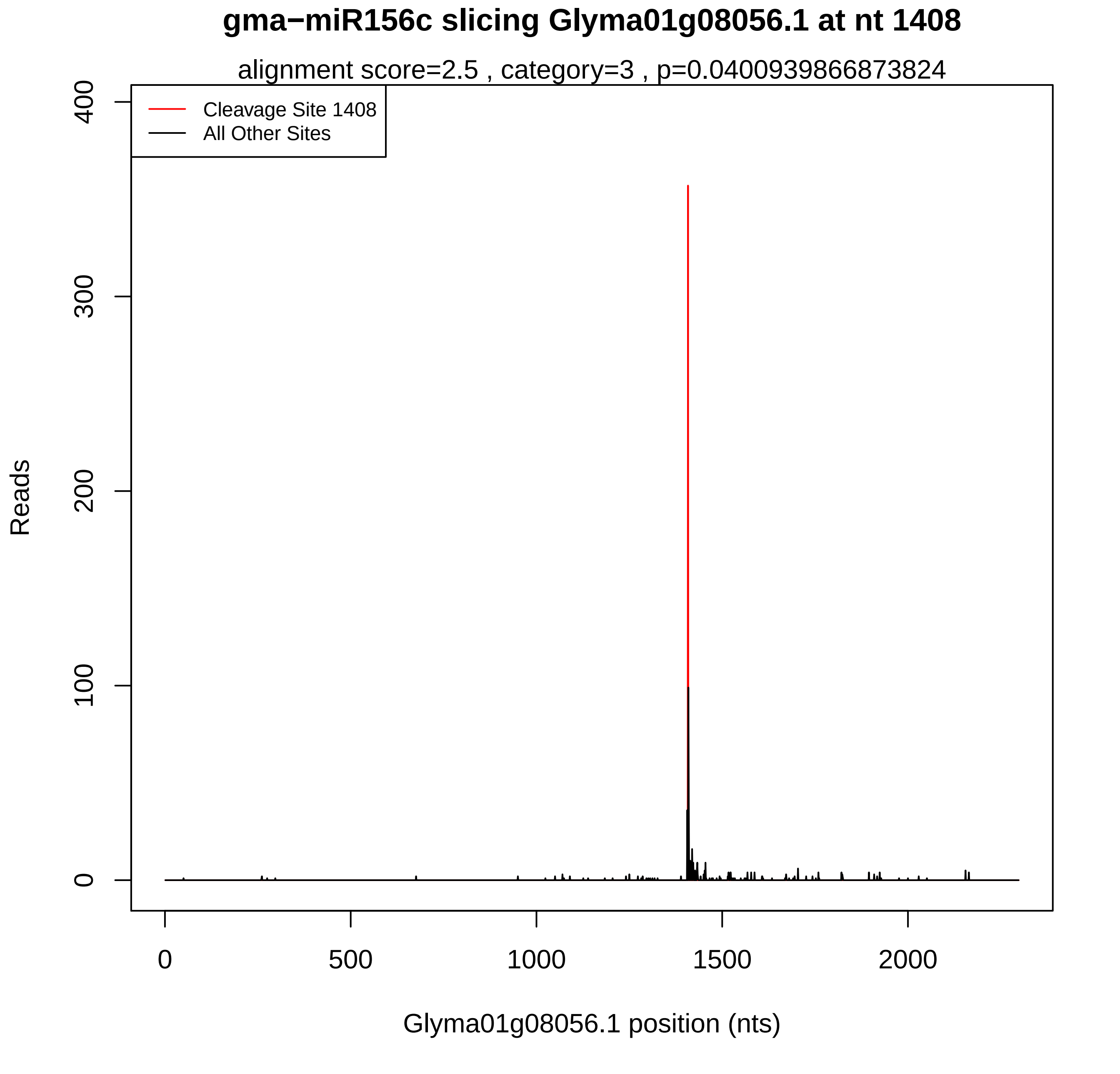

gma−miR156c slicing Glyma01g08056.1 at nt 1408
alignment score=2.5 , category=3 , p=0.0400939866873824
Cleavage Site 1408
All Other Sites
Reads
0
100
200
300
400
0
500
1000
1500
2000
Glyma01g08056.1 position (nts)

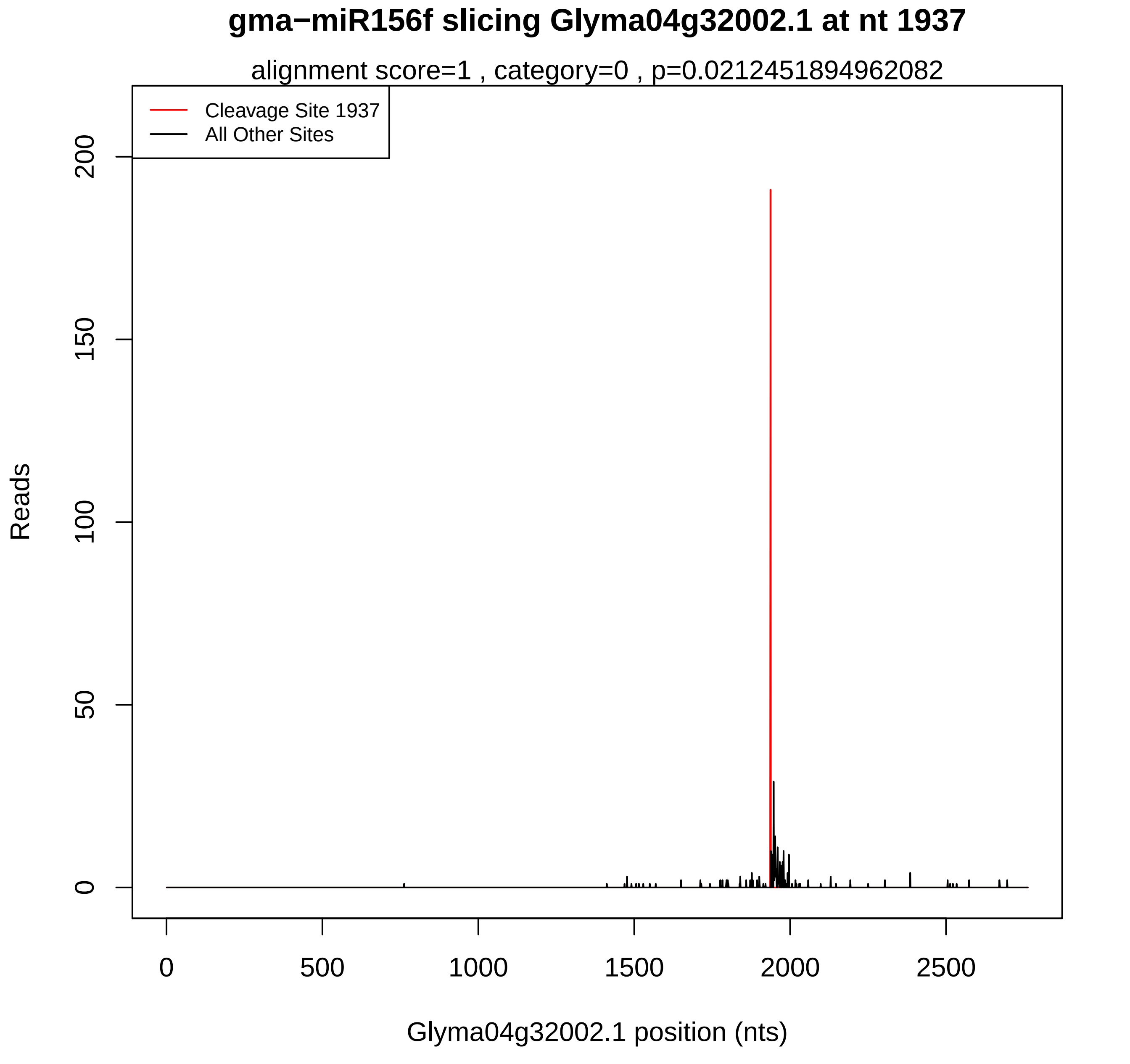

gma−miR156f slicing Glyma04g32002.1 at nt 1937
alignment score=1 , category=0 , p=0.0212451894962082
Cleavage Site 1937
All Other Sites
Reads
0
50
100
150
200
0
500
1000
1500
2000
2500
Glyma04g32002.1 position (nts)

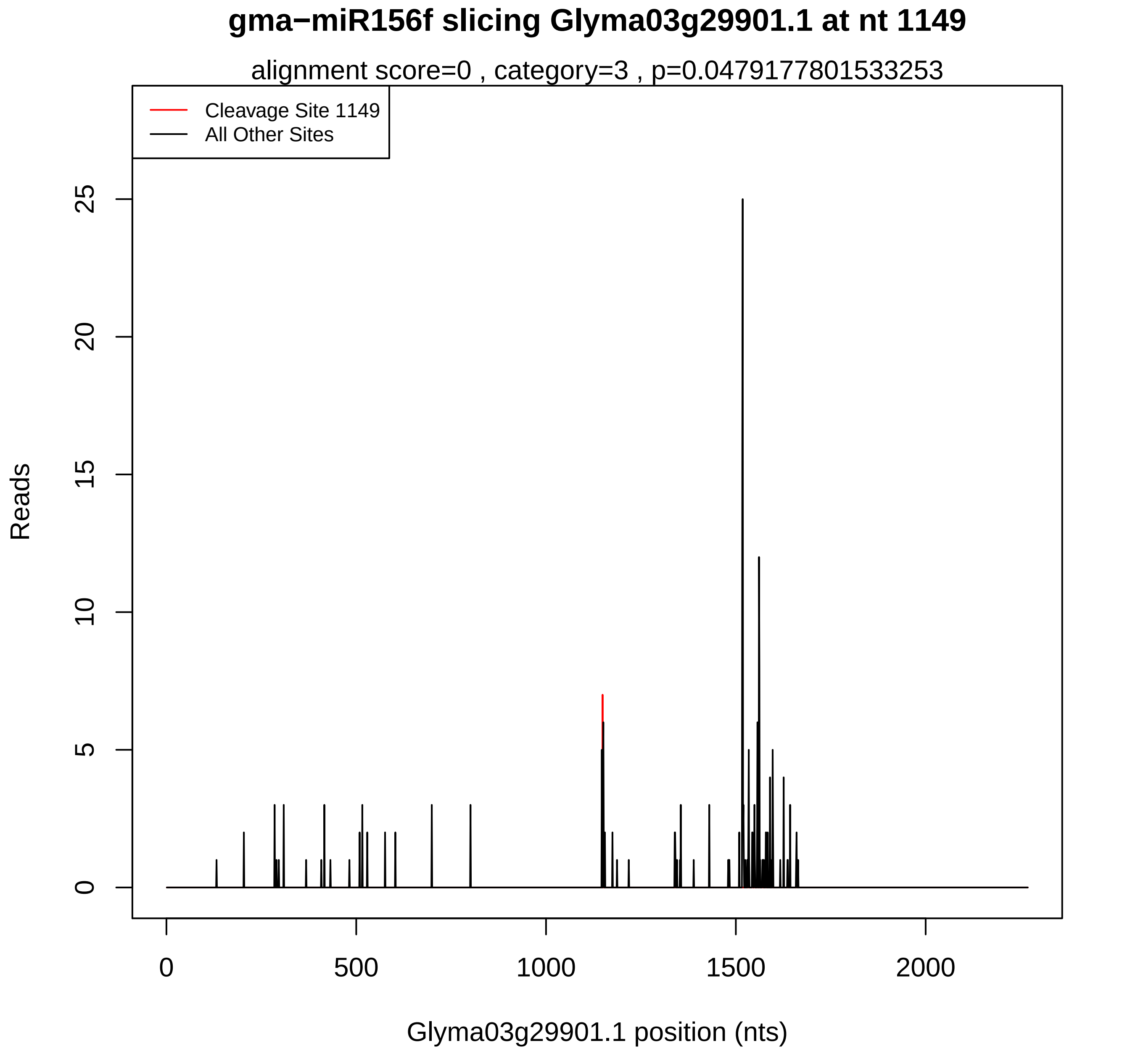

gma−miR156f slicing Glyma03g29901.1 at nt 1149
alignment score=0 , category=3 , p=0.0479177801533253
Cleavage Site 1149
All Other Sites
Reads
0
5
10
15
20
25
0
500
1000
1500
2000
Glyma03g29901.1 position (nts)

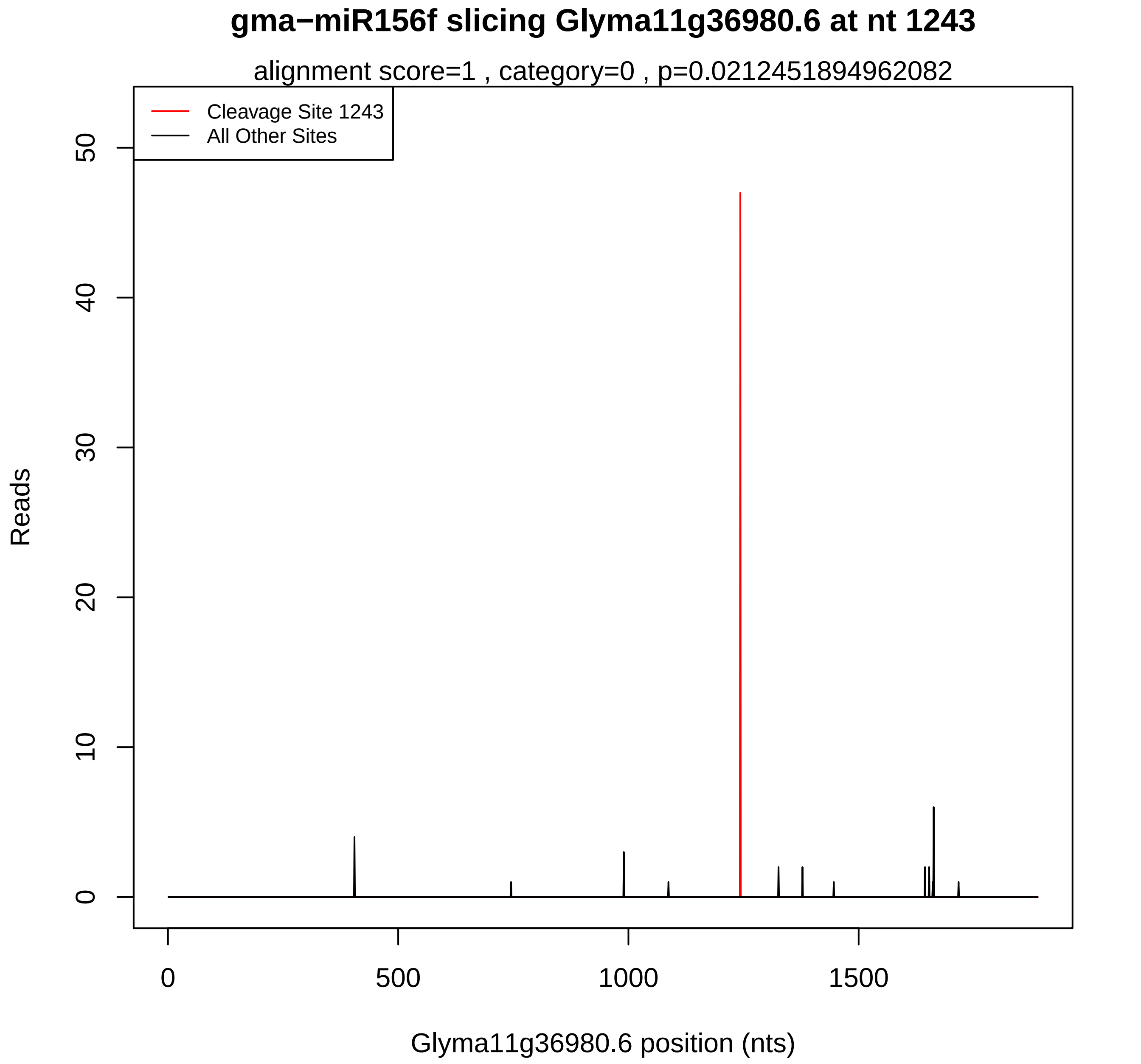
gma−miR156f slicing Glyma11g36980.6 at nt 1243
alignment score=1 , category=0 , p=0.0212451894962082
Cleavage Site 1243
All Other Sites
Reads
0
10
20
30
40
50
0
500
1000
1500
Glyma11g36980.6 position (nts)

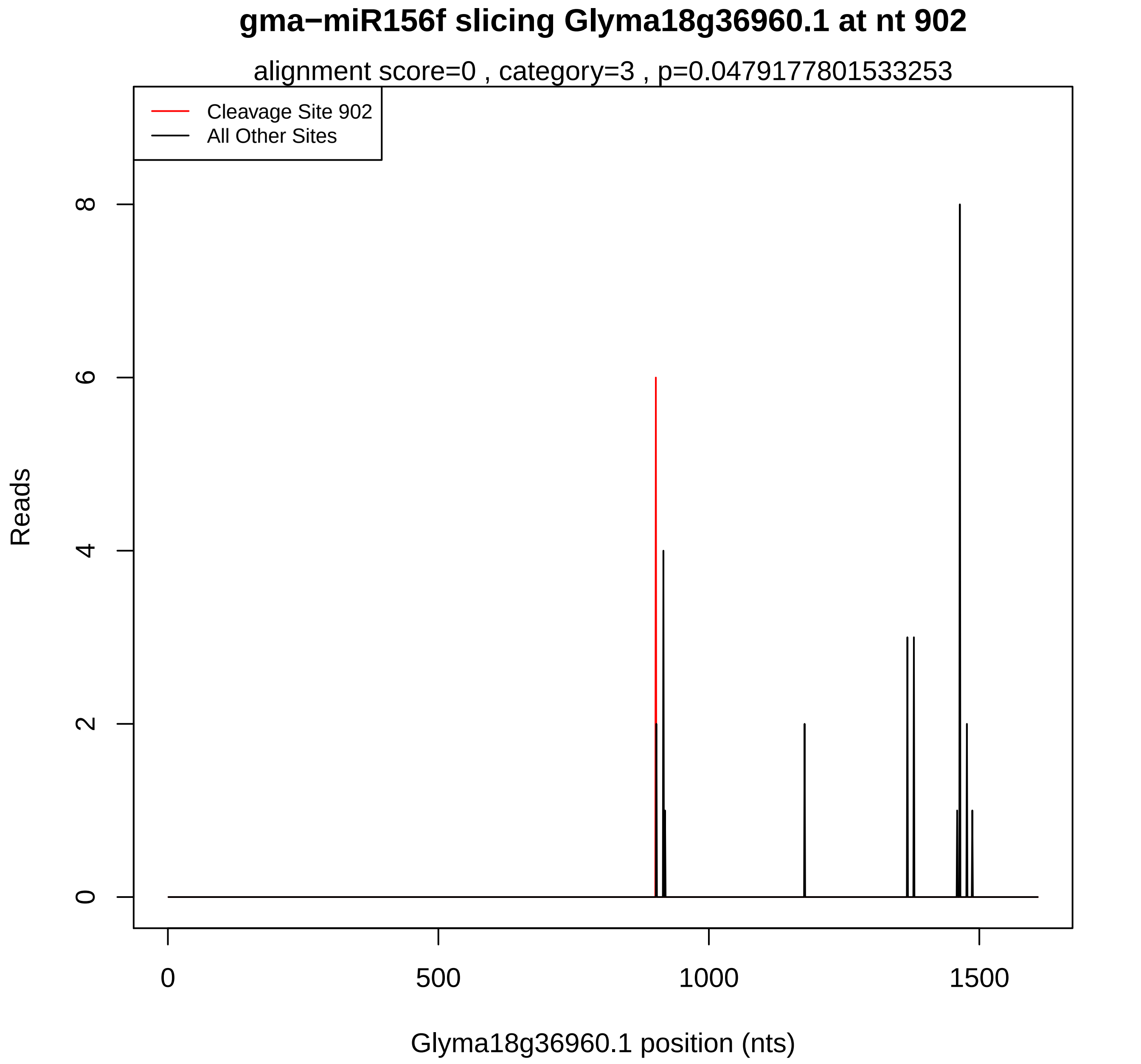

gma−miR156f slicing Glyma18g36960.1 at nt 902
alignment score=0 , category=3 , p=0.0479177801533253
Cleavage Site 902
All Other Sites
Reads
0
2
4
6
8
0
500
1000
1500
Glyma18g36960.1 position (nts)

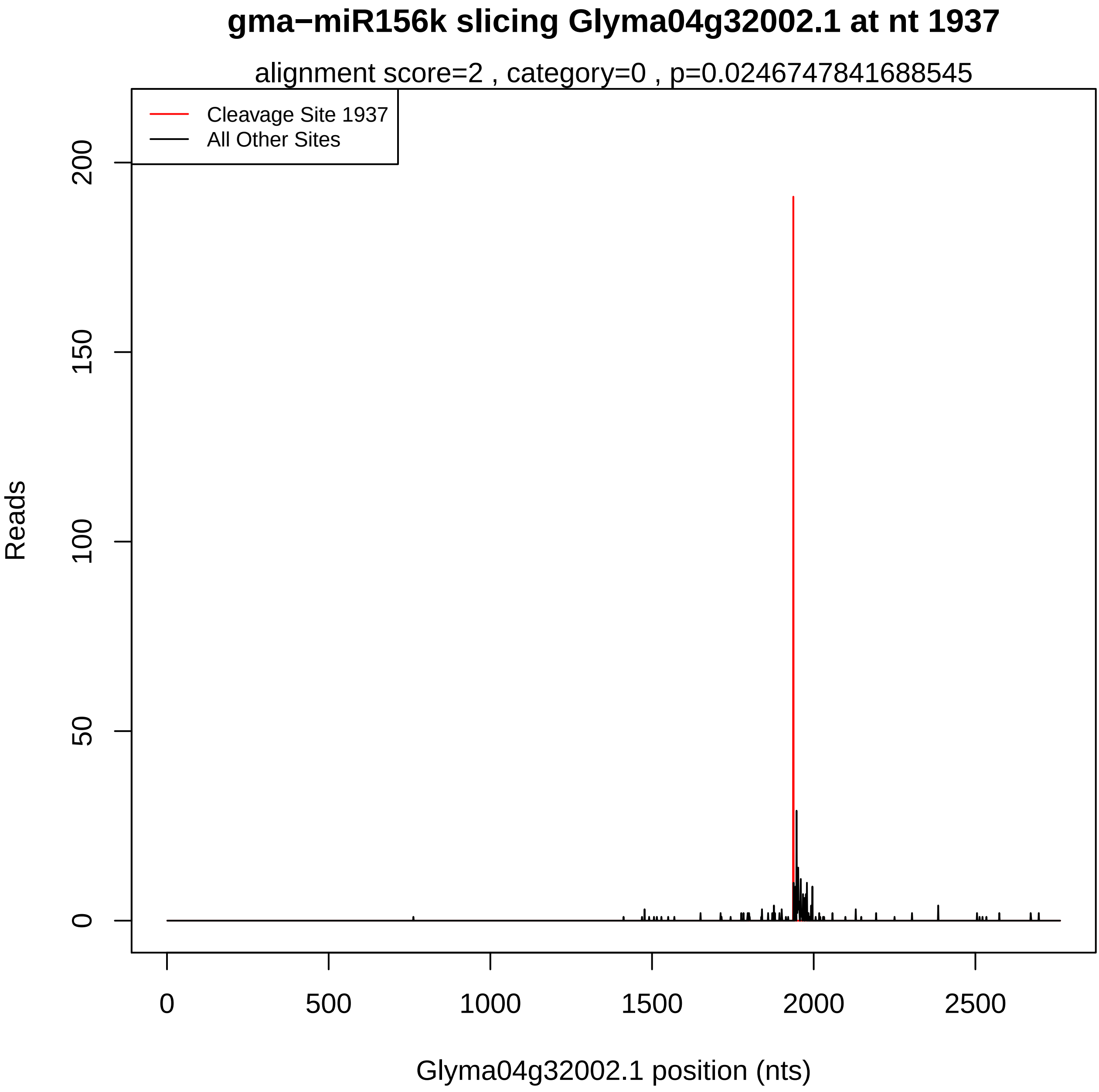
gma−miR156k slicing Glyma04g32002.1 at nt 1937
alignment score=2 , category=0 , p=0.0246747841688545
Cleavage Site 1937
All Other Sites
Reads
0
50
100
150
200
0
500
1000
1500
2000
2500
Glyma04g32002.1 position (nts)

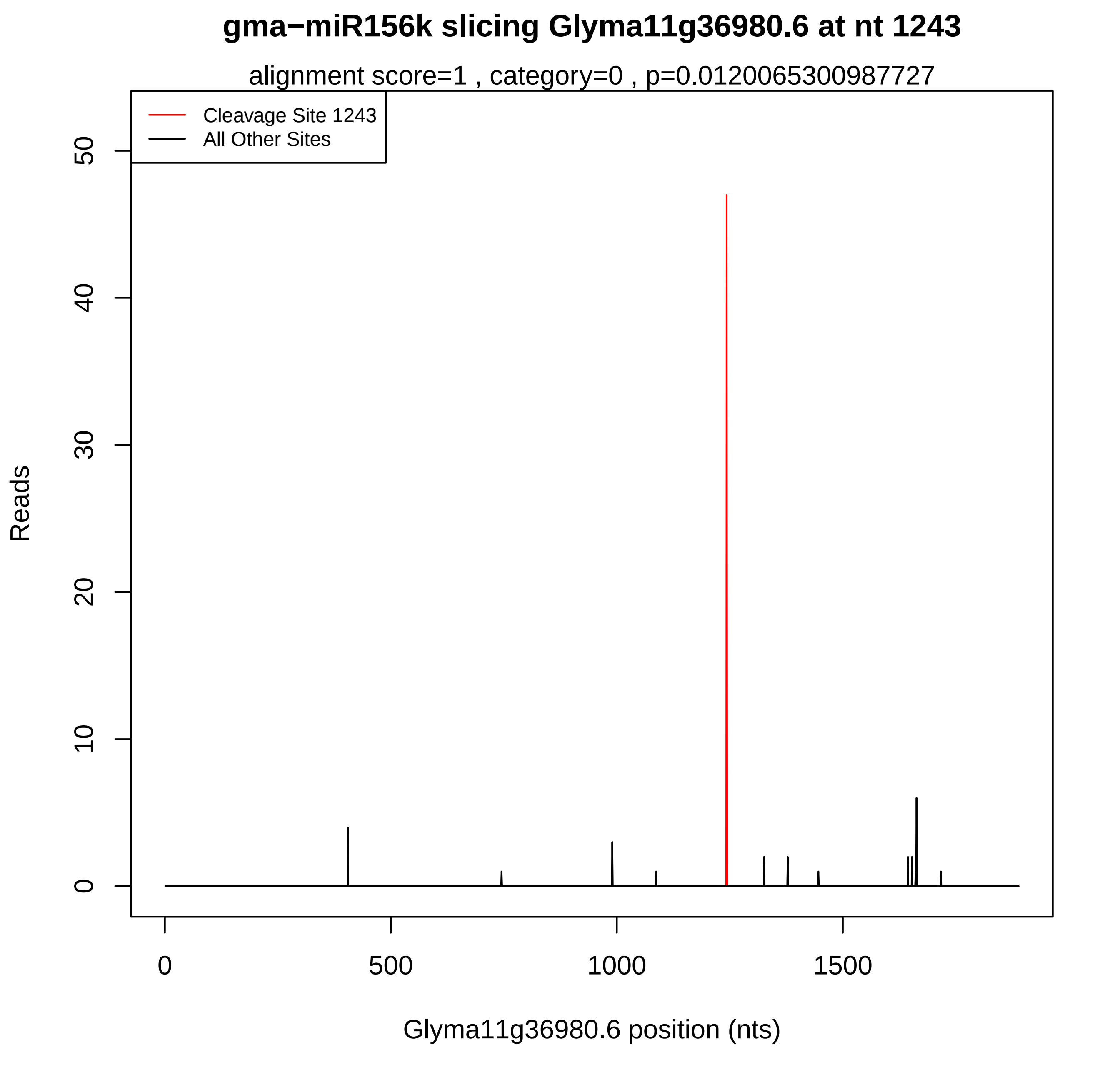

gma−miR156k slicing Glyma11g36980.6 at nt 1243
alignment score=1 , category=0 , p=0.0120065300987727
Cleavage Site 1243
All Other Sites
Reads
0
10
20
30
40
50
0
500
1000
1500
Glyma11g36980.6 position (nts)

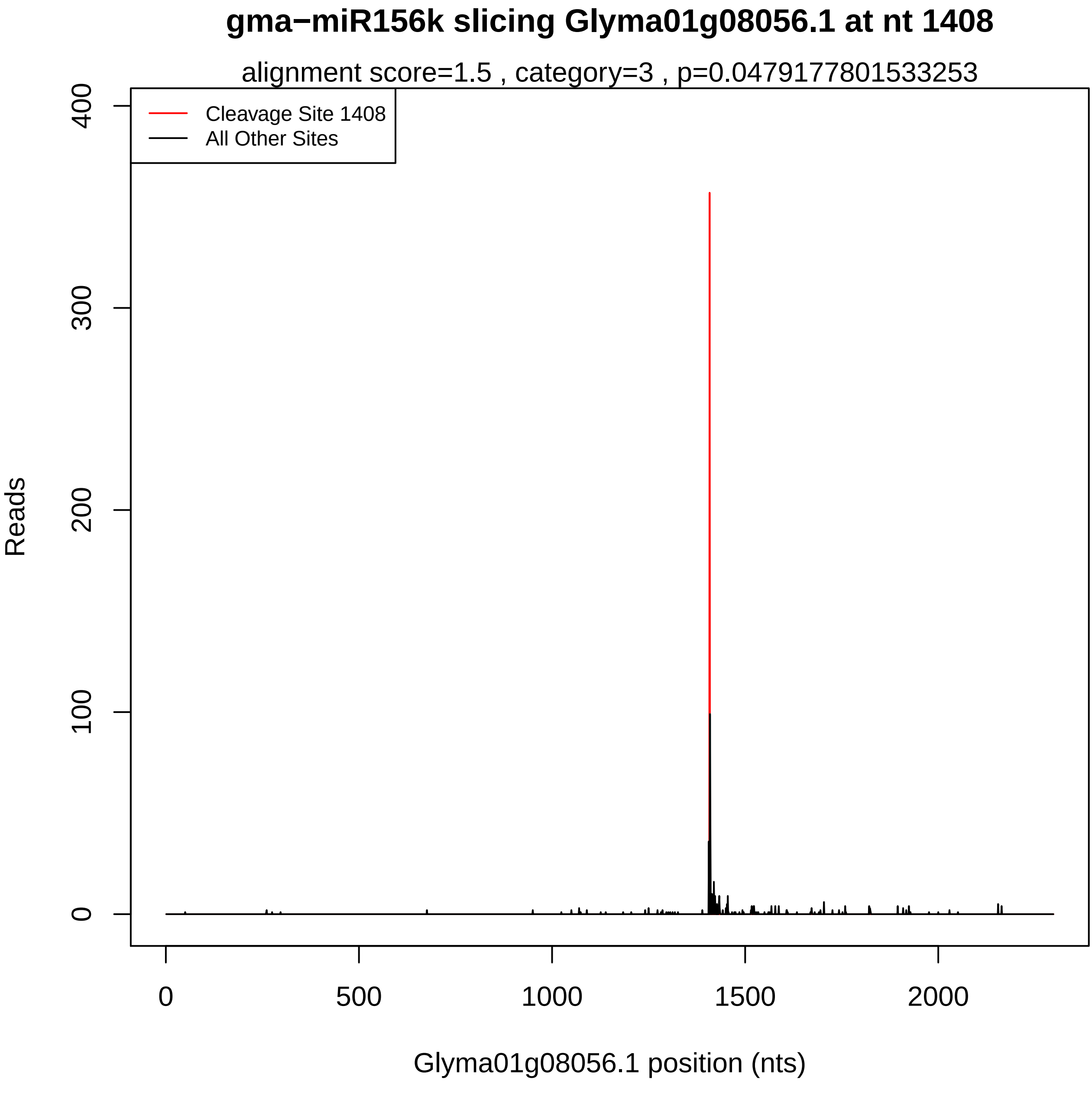
gma−miR156k slicing Glyma01g08056.1 at nt 1408
alignment score=1.5 , category=3 , p=0.0479177801533253
Cleavage Site 1408
All Other Sites
Reads
0
100
200
300
400
0
500
1000
1500
2000
Glyma01g08056.1 position (nts)

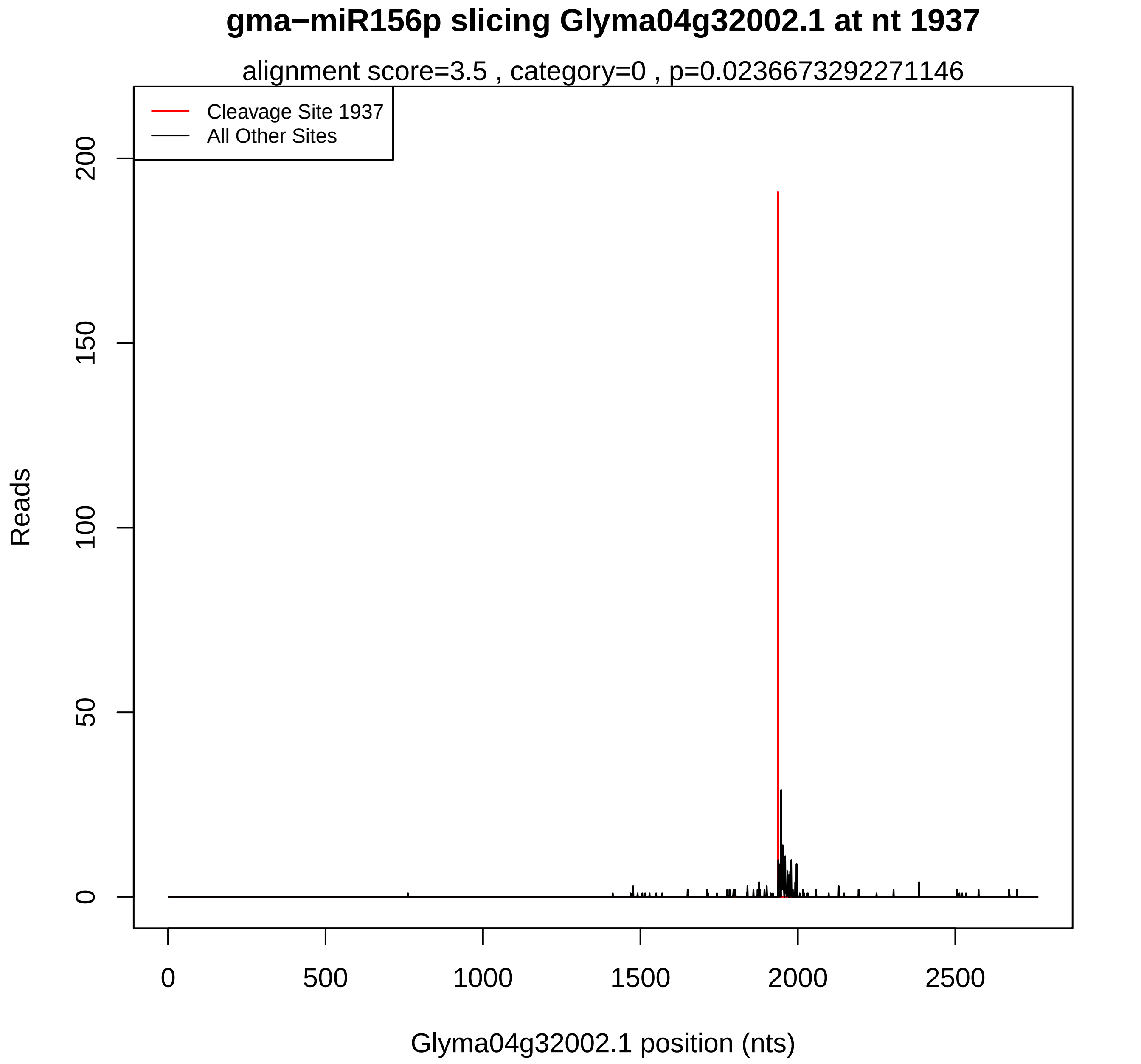
gma−miR156p slicing Glyma04g32002.1 at nt 1937
alignment score=3.5 , category=0 , p=0.0236673292271146
Cleavage Site 1937
All Other Sites
Reads
0
50
100
150
200
0
500
1000
1500
2000
2500
Glyma04g32002.1 position (nts)

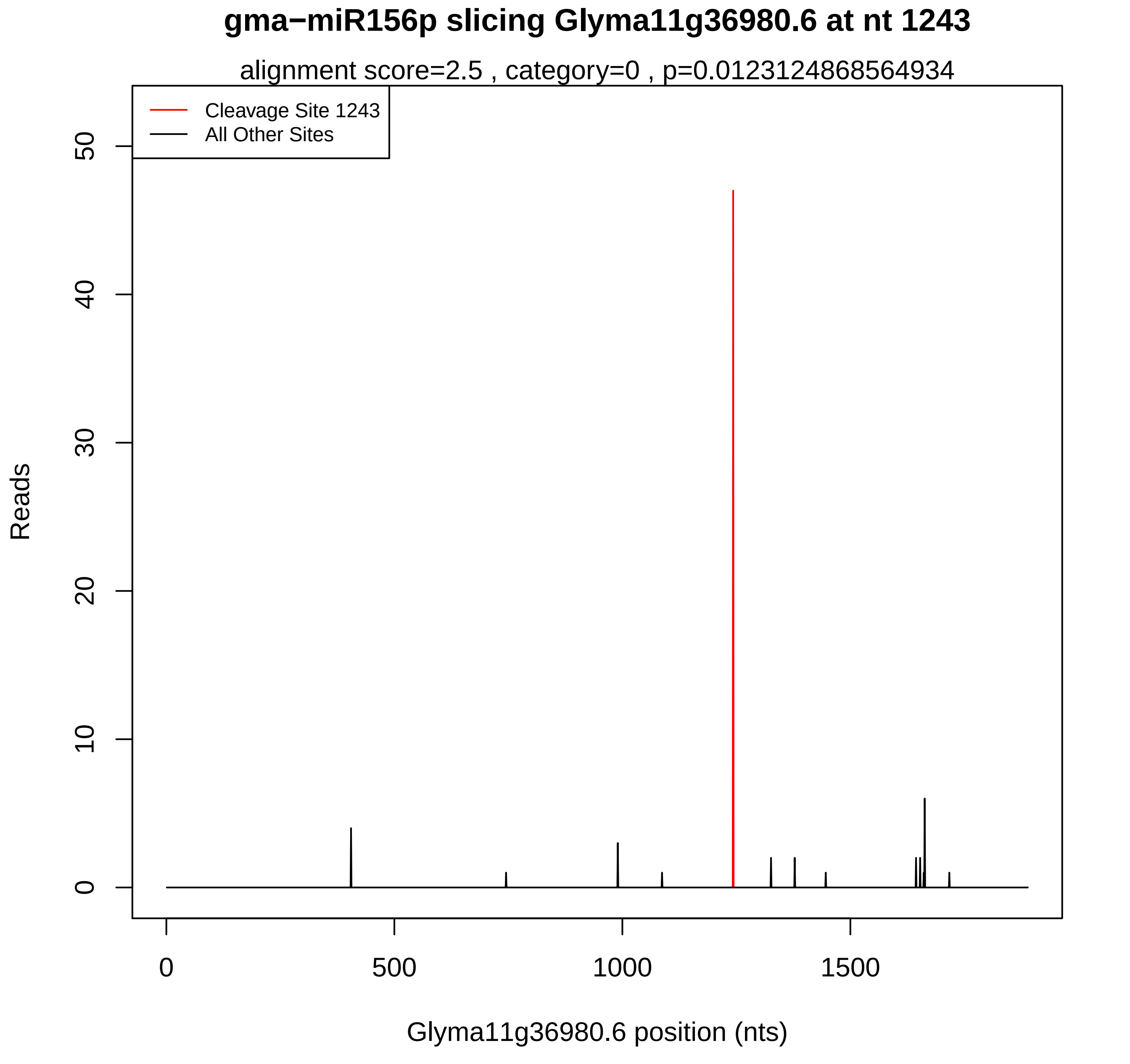

gma−miR156p slicing Glyma11g36980.6 at nt 1243
alignment score=2.5 , category=0 , p=0.0123124868564934
Cleavage Site 1243
All Other Sites
Reads
0
10
20
30
40
50
0
500
1000
1500
Glyma11g36980.6 position (nts)

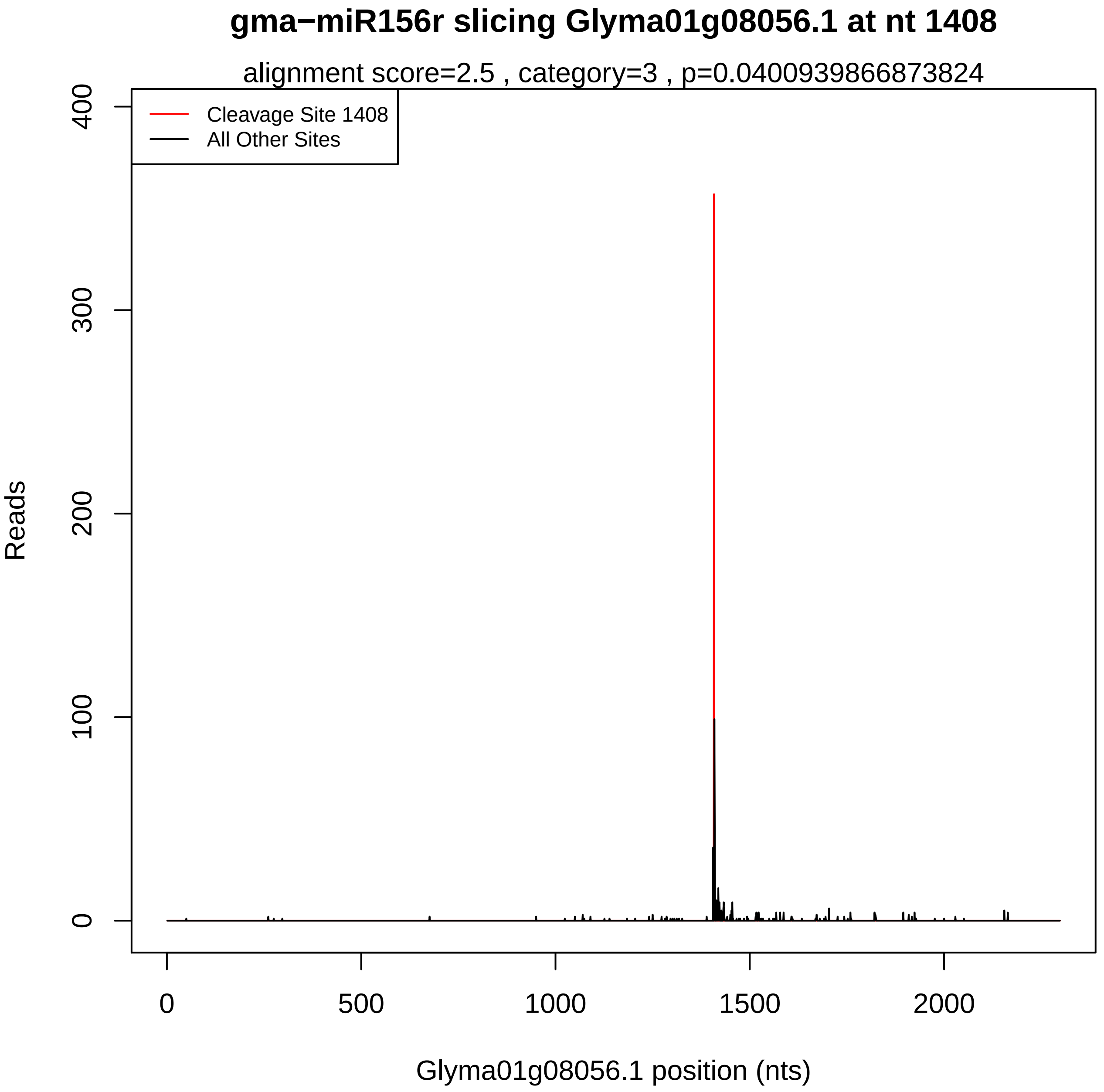

gma−miR156r slicing Glyma01g08056.1 at nt 1408
alignment score=2.5 , category=3 , p=0.0400939866873824
Cleavage Site 1408
All Other Sites
Reads
0
100
200
300
400
0
500
1000
1500
2000
Glyma01g08056.1 position (nts)

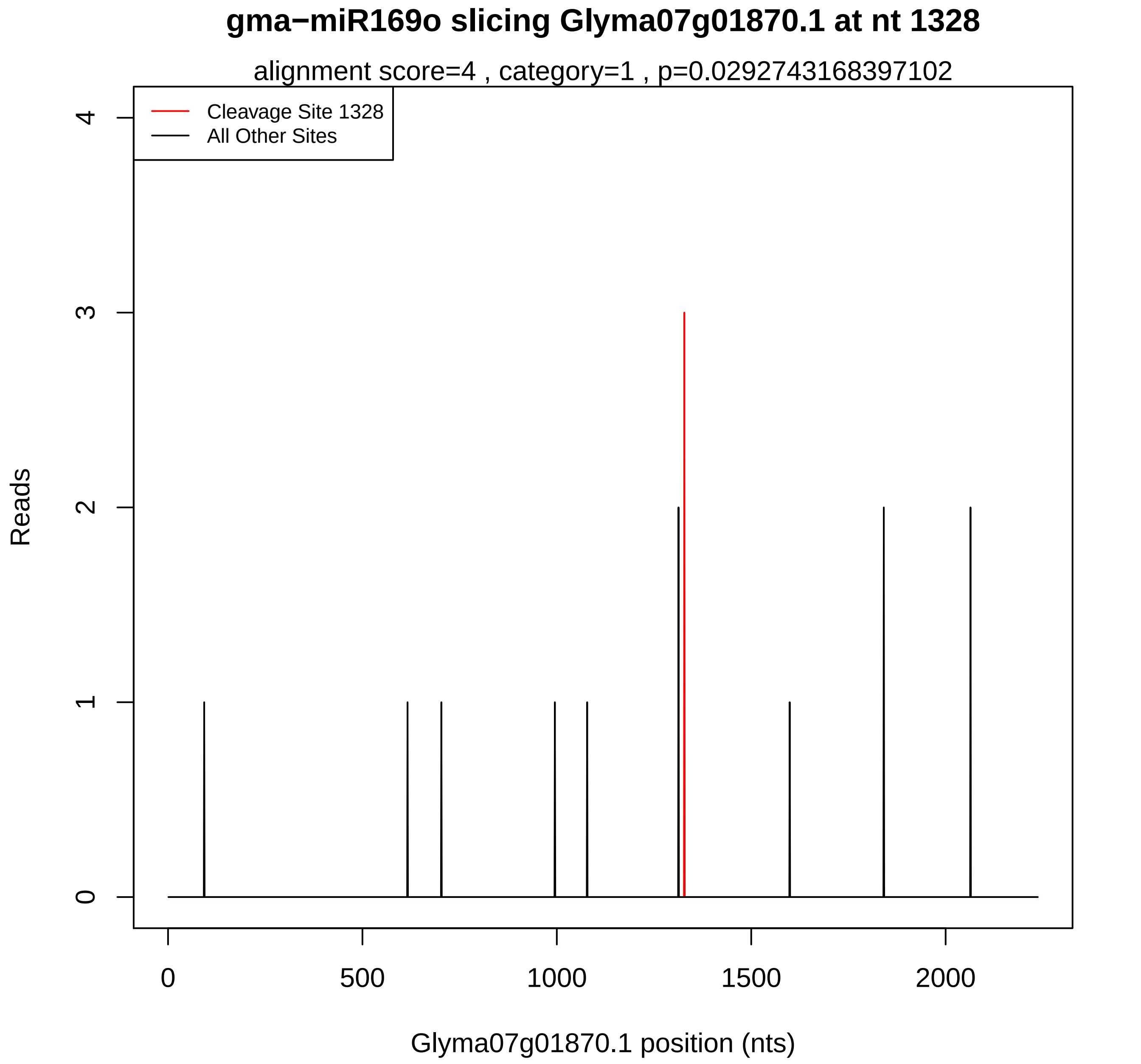
gma−miR169o slicing Glyma07g01870.1 at nt 1328
alignment score=4 , category=1 , p=0.0292743168397102
Cleavage Site 1328
All Other Sites
Reads
0
1
2
3
4
0
500
1000
1500
2000
Glyma07g01870.1 position (nts)

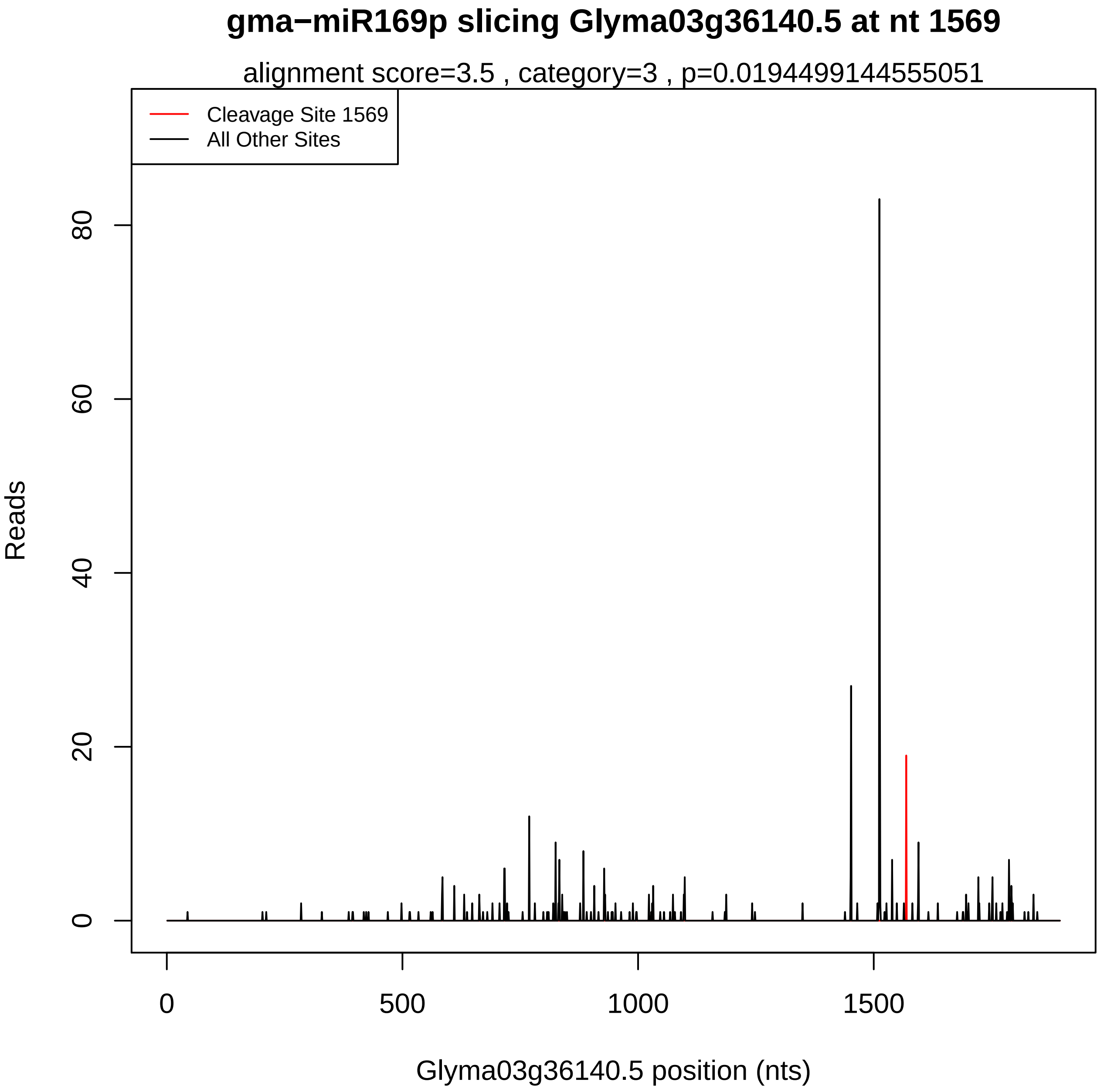
gma−miR169p slicing Glyma03g36140.5 at nt 1569
alignment score=3.5 , category=3 , p=0.0194499144555051
Cleavage Site 1569
All Other Sites
Reads
0
20
40
60
80
0
500
1000
1500
Glyma03g36140.5 position (nts)

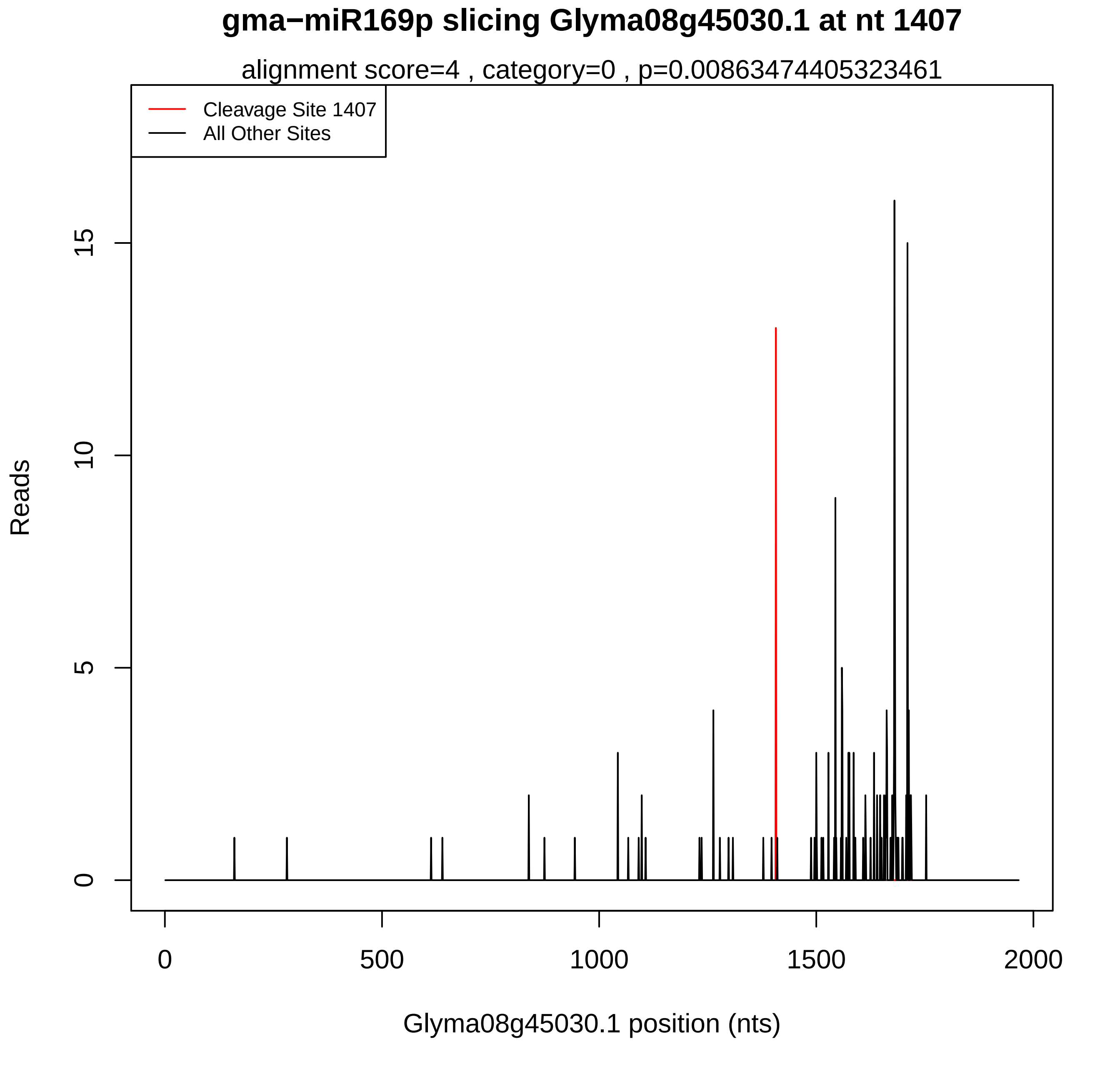
gma−miR169p slicing Glyma08g45030.1 at nt 1407
alignment score=4 , category=0 , p=0.00863474405323461
Cleavage Site 1407
All Other Sites
Reads
0
5
10
15
0
500
1000
1500
2000
Glyma08g45030.1 position (nts)

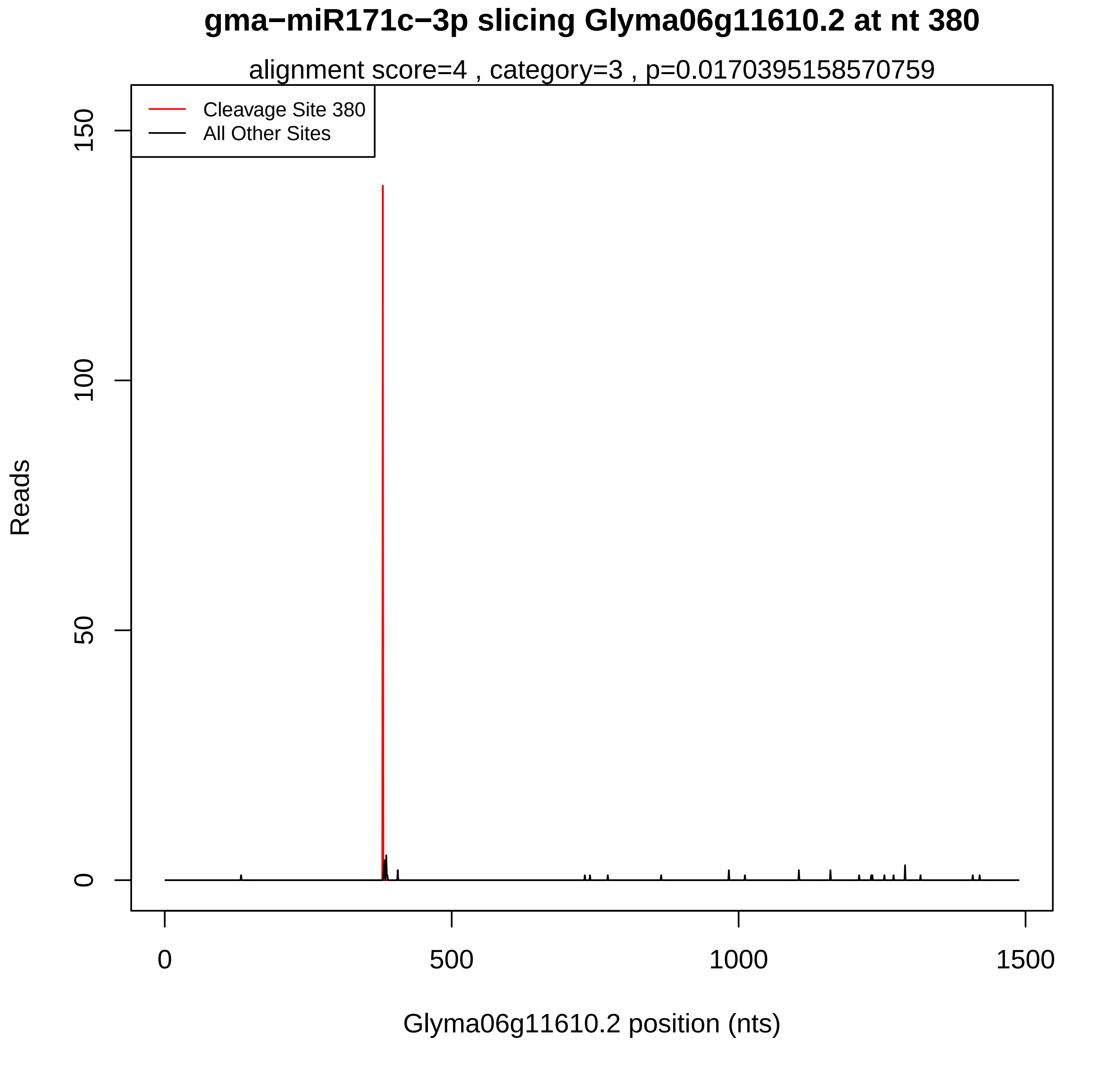

gma−miR171c−3p slicing Glyma06g11610.2 at nt 380
alignment score=4 , category=3 , p=0.0170395158570759
Cleavage Site 380
All Other Sites
Reads
0
50
100
150
0
500
1000
1500
Glyma06g11610.2 position (nts)

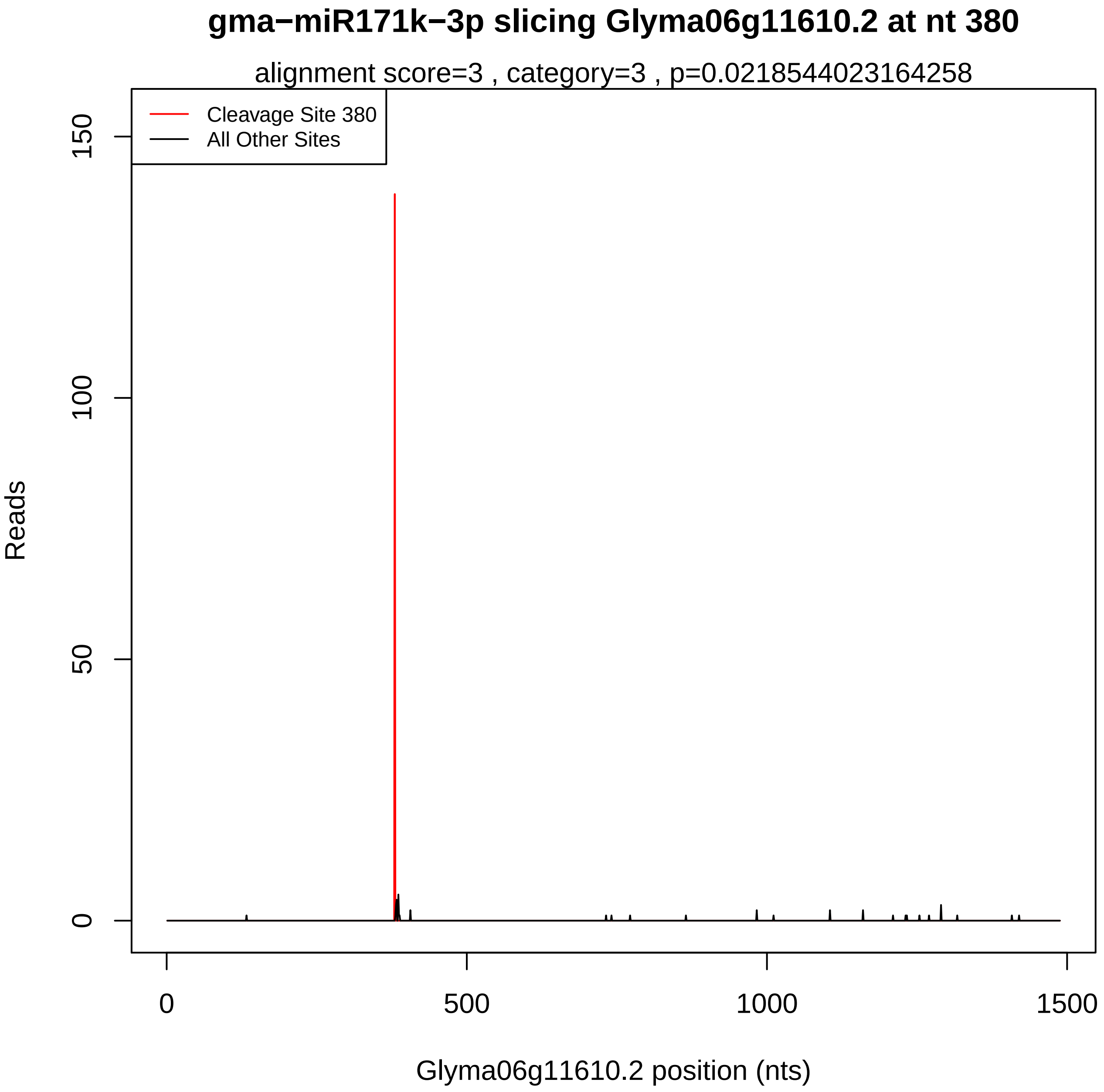
gma−miR171k−3p slicing Glyma06g11610.2 at nt 380
alignment score=3 , category=3 , p=0.0218544023164258
Cleavage Site 380
All Other Sites
Reads
150
100
50
0
0
500
1000
1500
Glyma06g11610.2 position (nts)

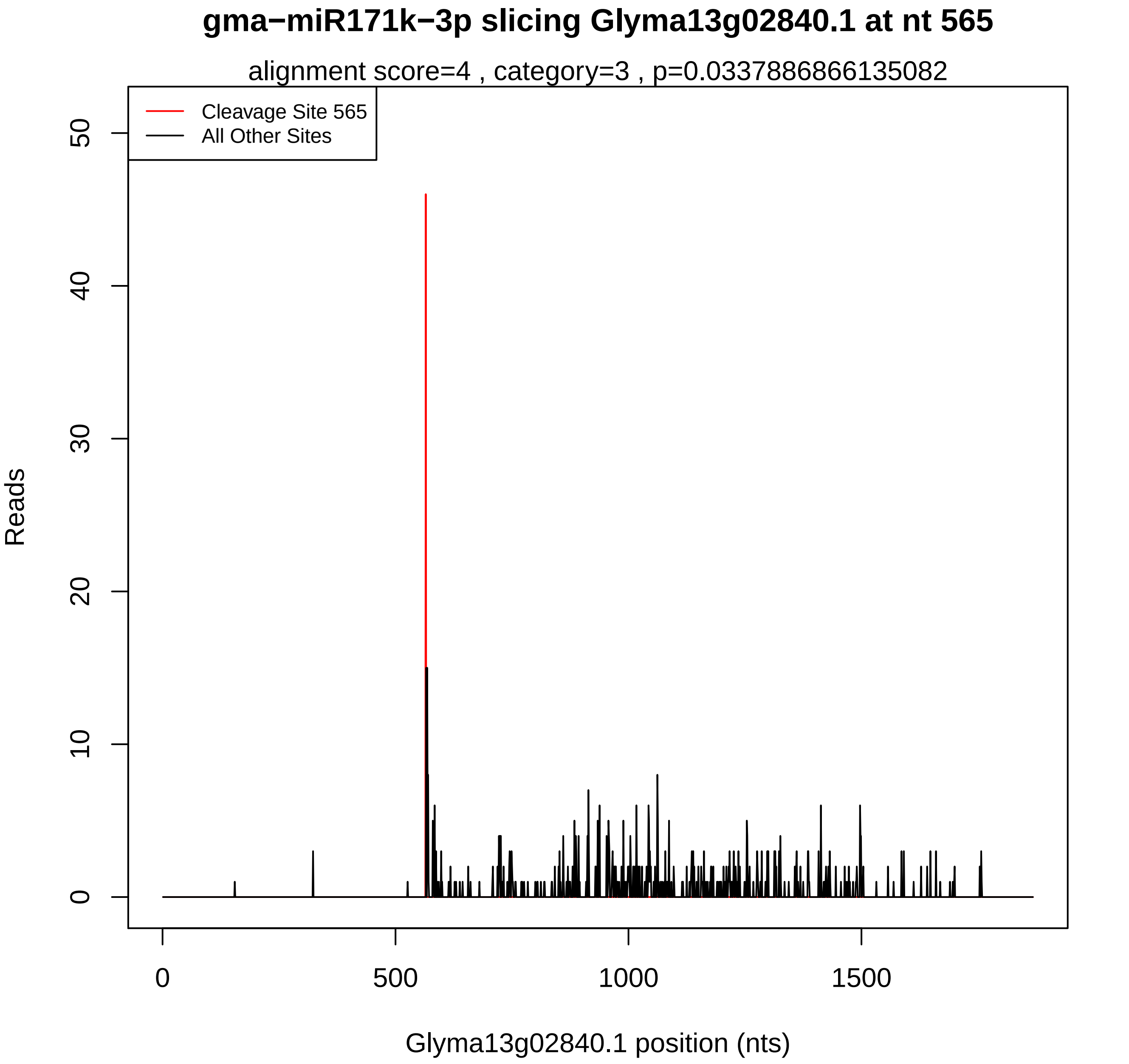
gma−miR171k−3p slicing Glyma13g02840.1 at nt 565
alignment score=4 , category=3 , p=0.0337886866135082
Cleavage Site 565
All Other Sites
Reads
0
10
20
30
40
50
0
500
1000
1500
Glyma13g02840.1 position (nts)

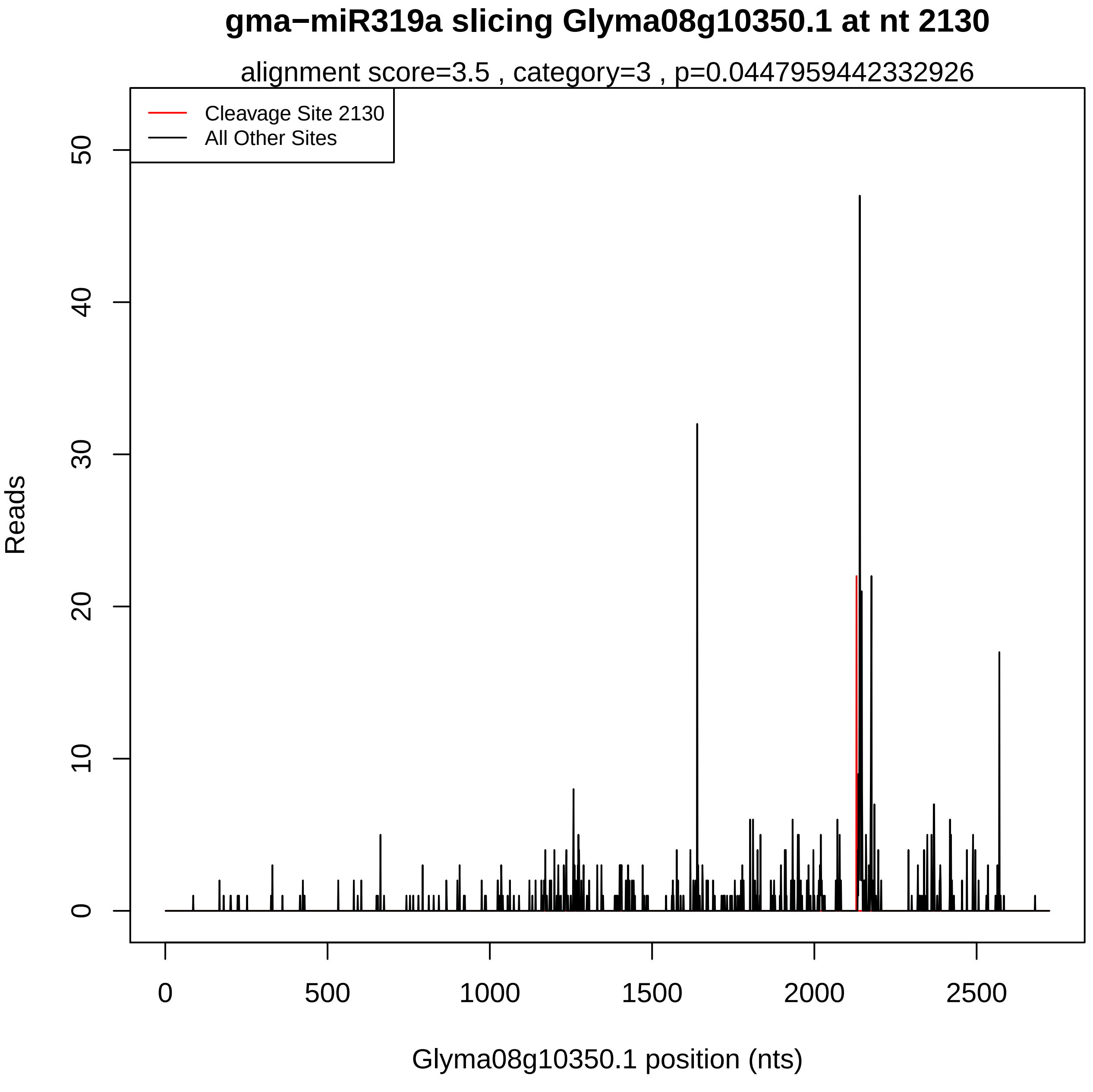
gma−miR319a slicing Glyma08g10350.1 at nt 2130
alignment score=3.5 , category=3 , p=0.0447959442332926
Cleavage Site 2130
All Other Sites
Reads
0
10
20
30
40
50
0
500
1000
1500
2000
2500
Glyma08g10350.1 position (nts)

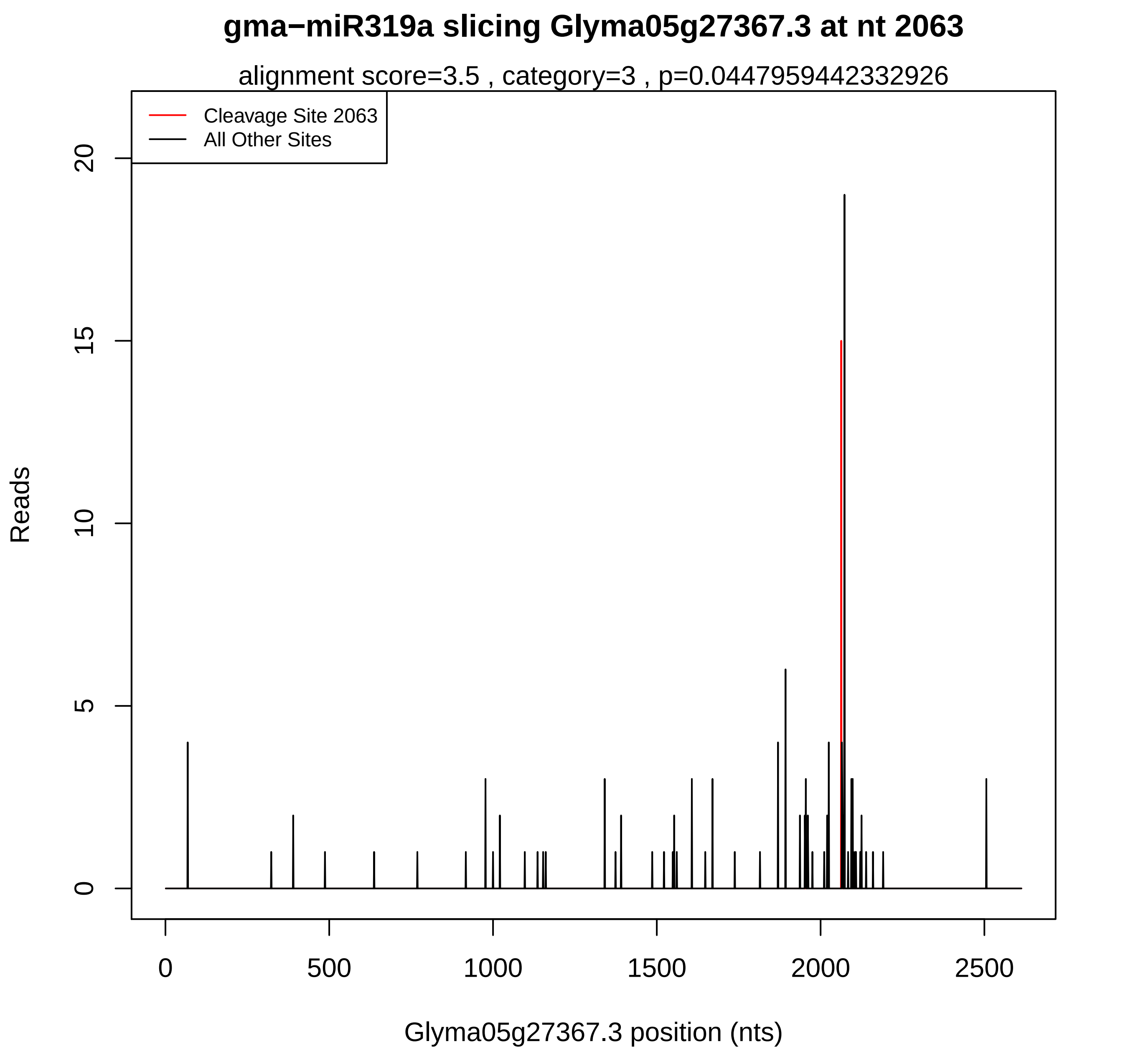
gma−miR319a slicing Glyma05g27367.3 at nt 2063
alignment score=3.5 , category=3 , p=0.0447959442332926
Cleavage Site 2063
All Other Sites
Reads
0
5
10
15
20
0
500
1000
1500
2000
2500
Glyma05g27367.3 position (nts)

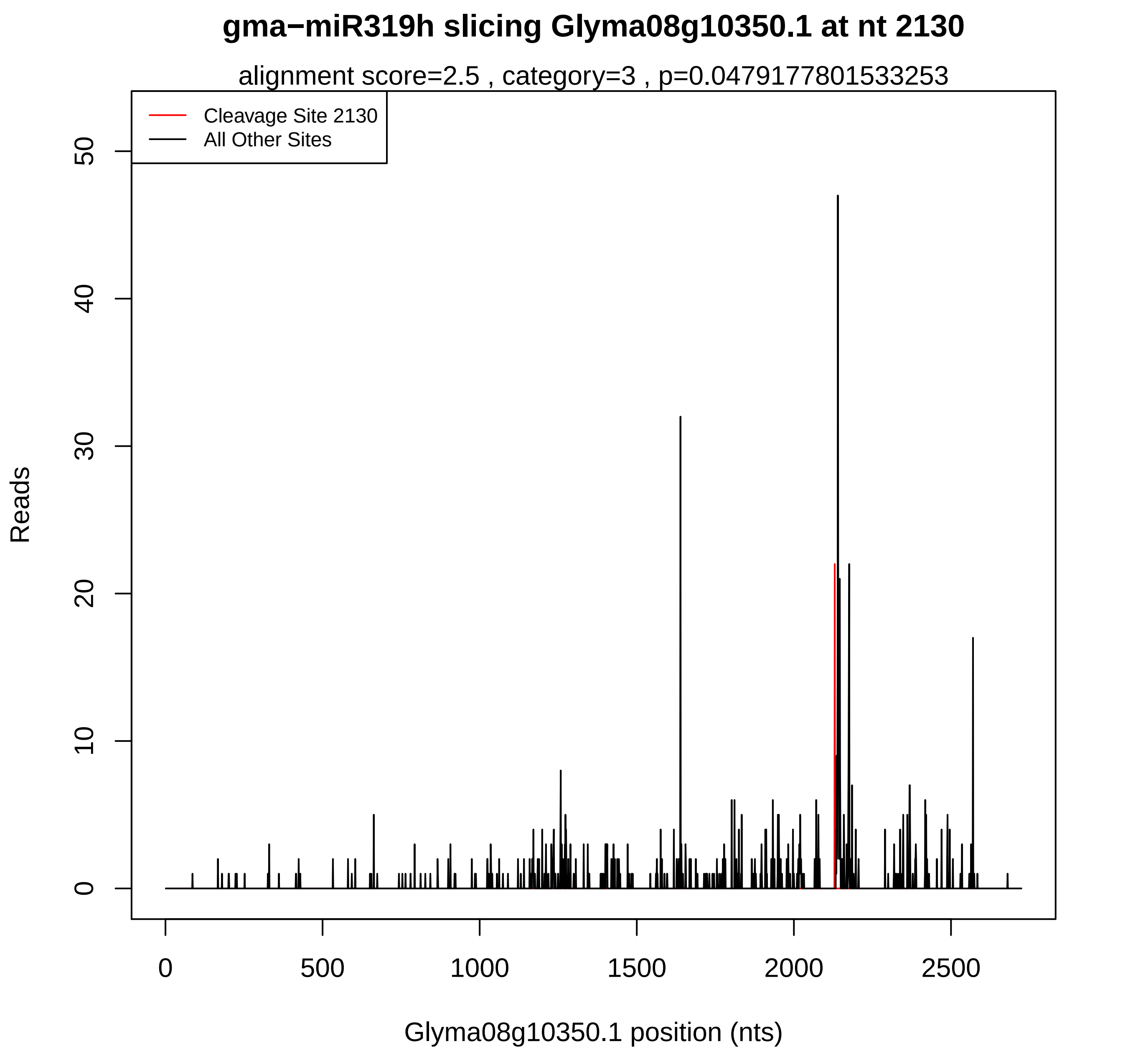

gma−miR319h slicing Glyma08g10350.1 at nt 2130
alignment score=2.5 , category=3 , p=0.0479177801533253
Cleavage Site 2130
All Other Sites
Reads
0
10
20
30
40
50
0
500
1000
1500
2000
2500
Glyma08g10350.1 position (nts)

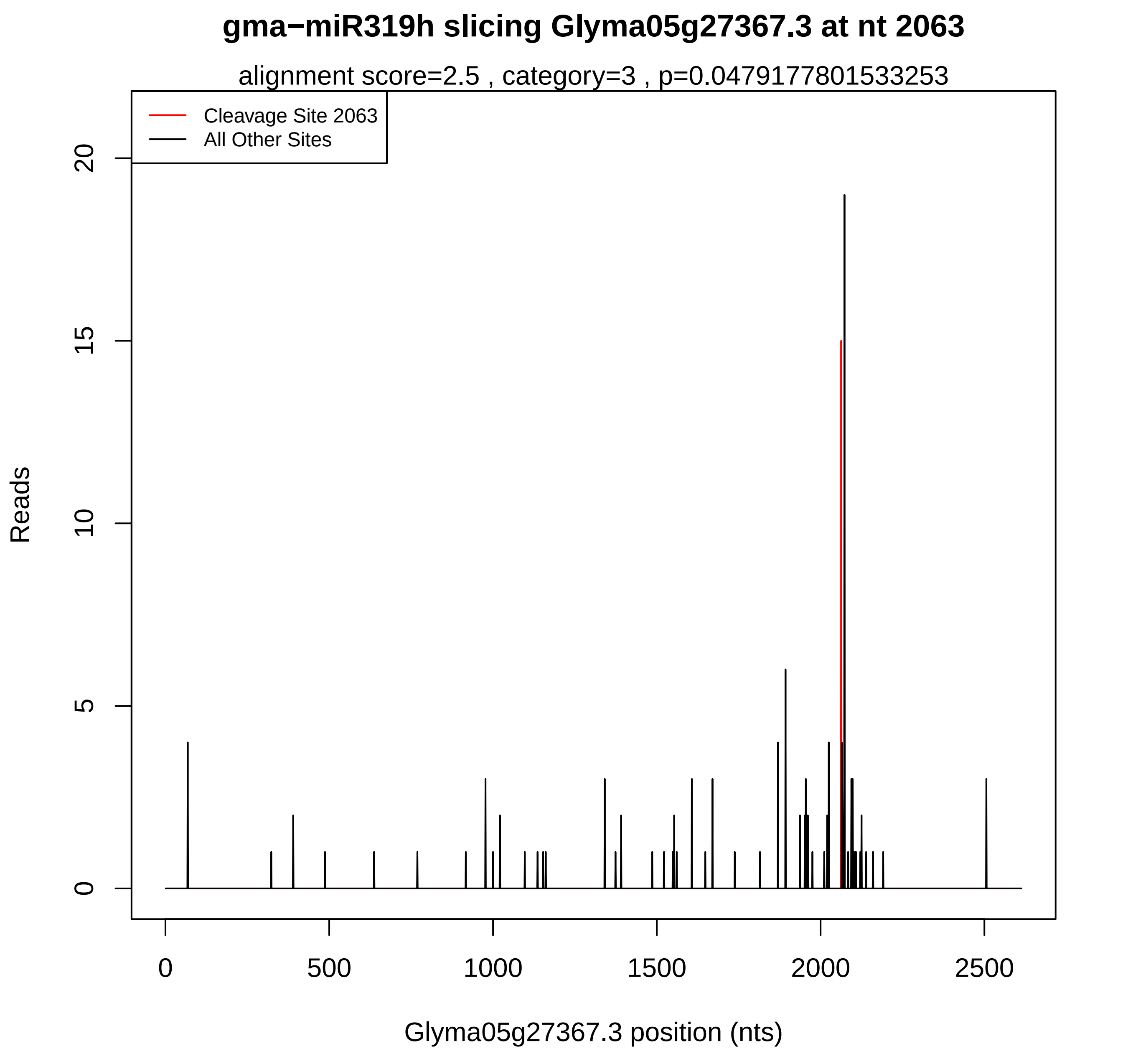

gma−miR319h slicing Glyma05g27367.3 at nt 2063
alignment score=2.5 , category=3 , p=0.0479177801533253
Cleavage Site 2063
All Other Sites
Reads
0
5
10
15
20
0
500
1000
1500
2000
2500
Glyma05g27367.3 position (nts)

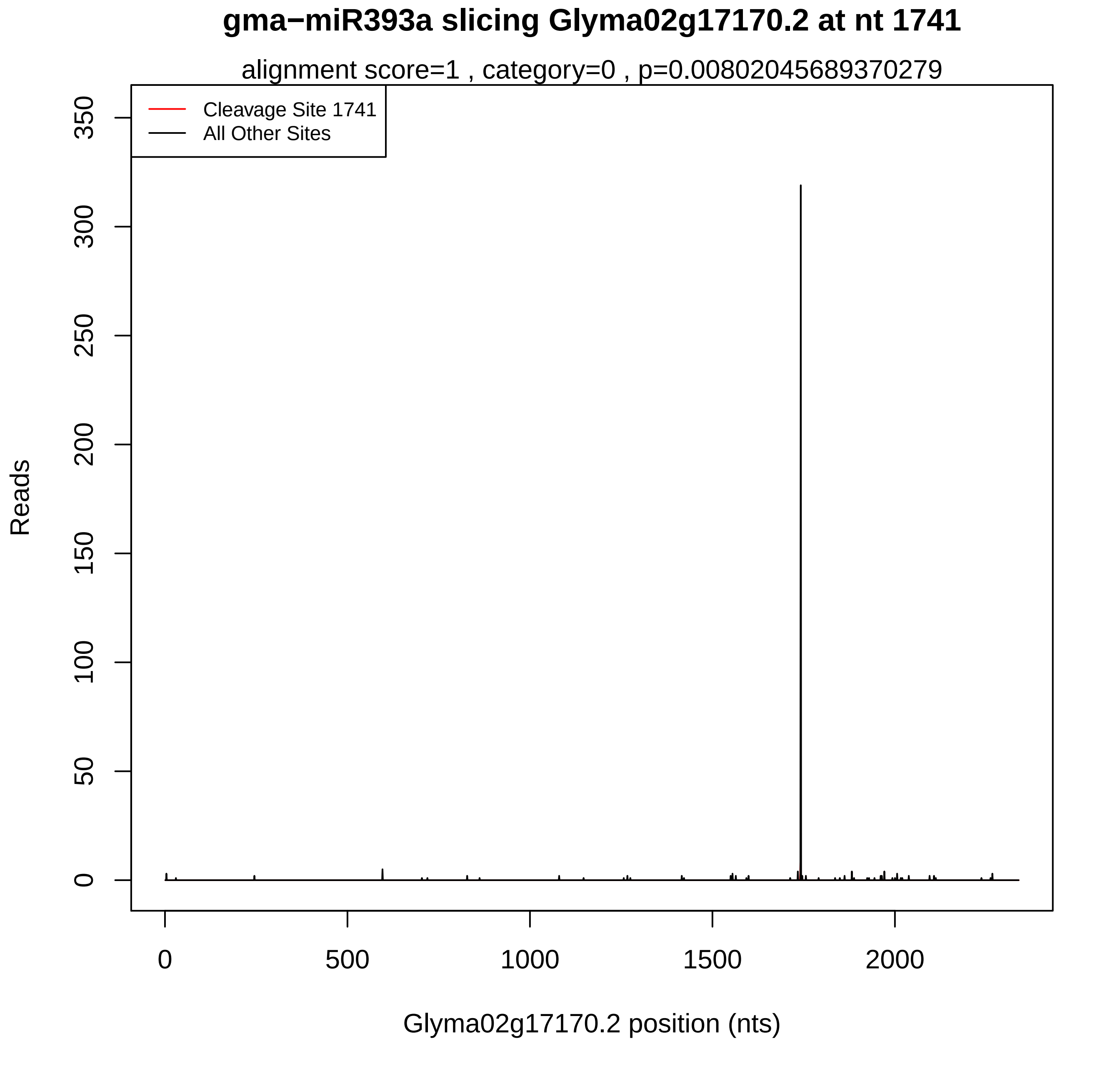
gma−miR393a slicing Glyma02g17170.2 at nt 1741
alignment score=1 , category=0 , p=0.00802045689370279
Cleavage Site 1741
All Other Sites
Reads
0
50
100
150
200
250
300
350
0
500
1000
1500
2000
Glyma02g17170.2 position (nts)

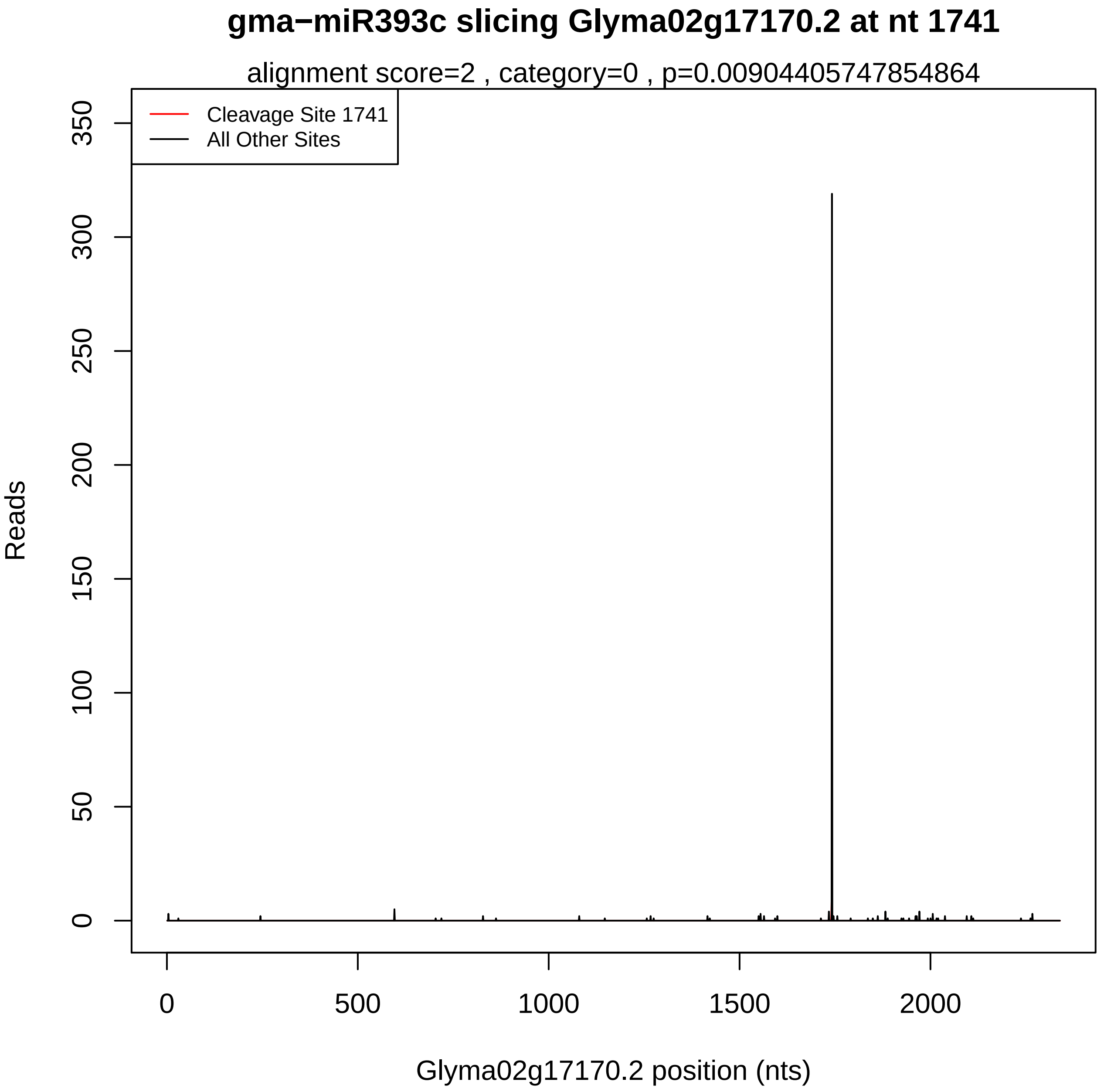
gma−miR393c slicing Glyma02g17170.2 at nt 1741
alignment score=2 , category=0 , p=0.00904405747854864
Cleavage Site 1741
All Other Sites
Reads
0
50
100
150
200
250
300
350
0
500
1000
1500
2000
Glyma02g17170.2 position (nts)

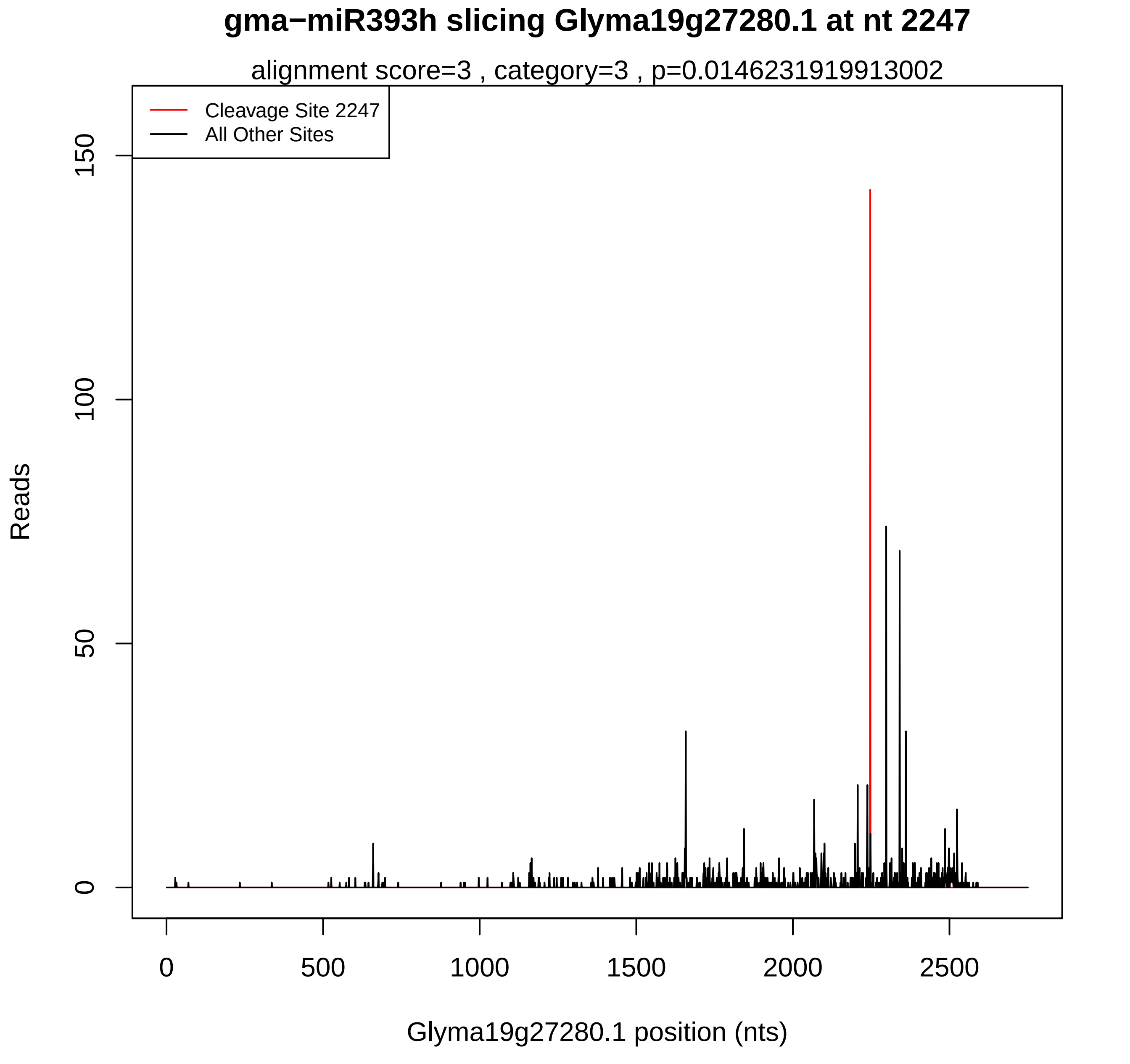
gma−miR393h slicing Glyma19g27280.1 at nt 2247
alignment score=3 , category=3 , p=0.0146231919913002
Cleavage Site 2247
All Other Sites
Reads
0
50
100
150
0
500
1000
1500
2000
2500
Glyma19g27280.1 position (nts)

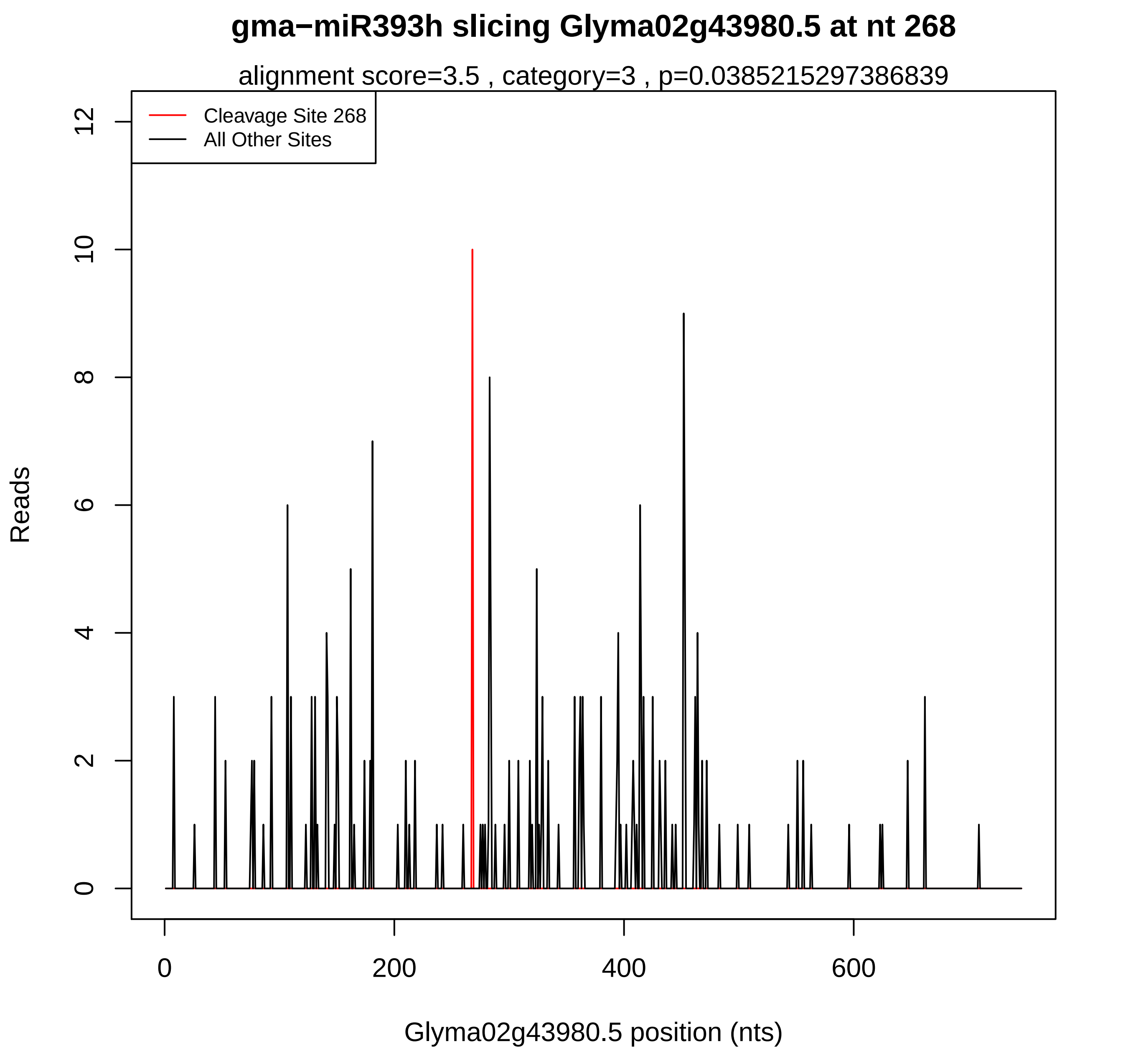

gma−miR393h slicing Glyma02g43980.5 at nt 268
alignment score=3.5 , category=3 , p=0.0385215297386839
Cleavage Site 268
All Other Sites
Reads
0
2
4
6
8
10
12
0
200
400
600
Glyma02g43980.5 position (nts)

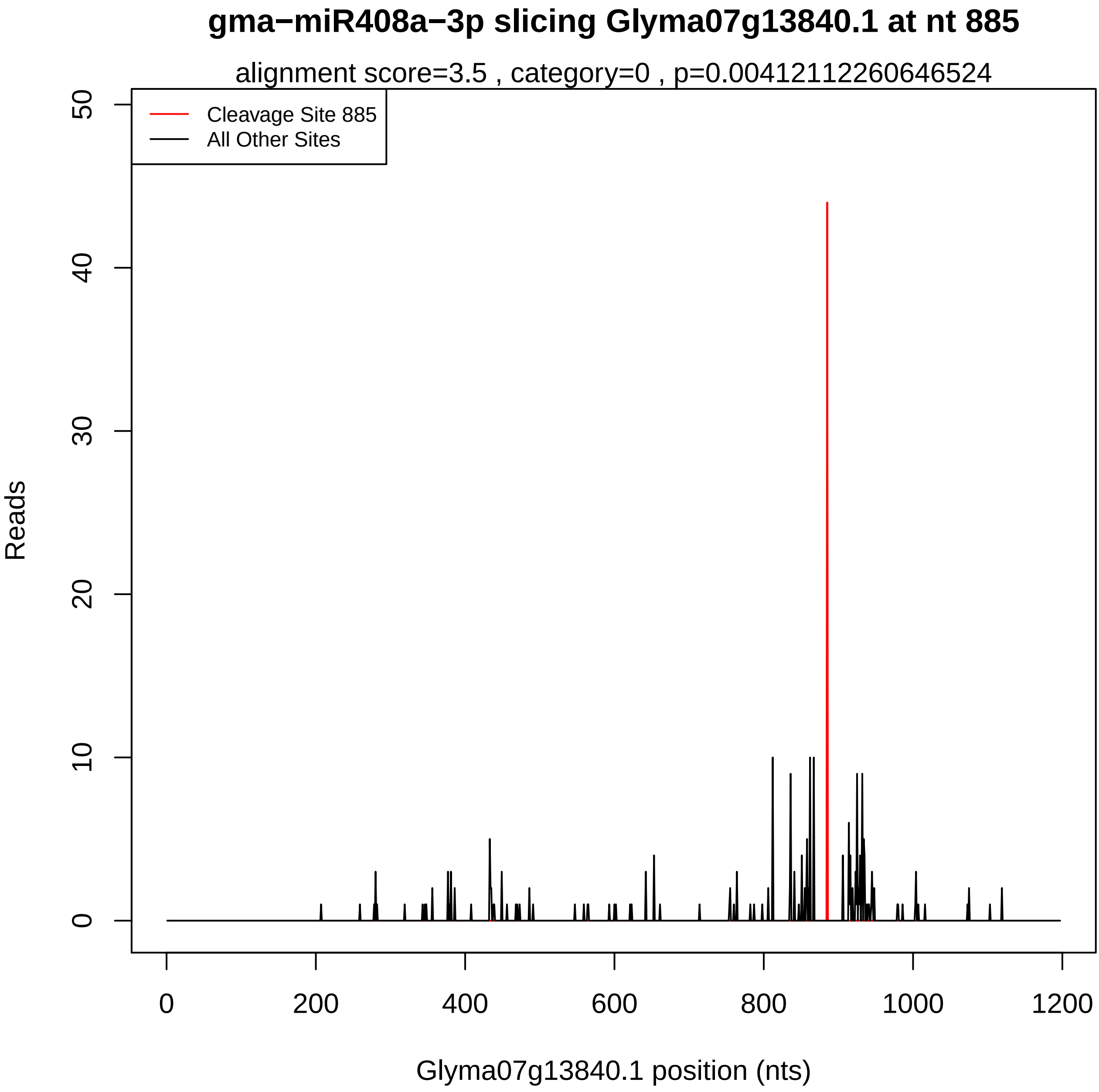

gma−miR408a−3p slicing Glyma07g13840.1 at nt 885
alignment score=3.5 , category=0 , p=0.00412112260646524
Cleavage Site 885
All Other Sites
Reads
0
10
20
30
40
50
0
200
400
600
800
1000
1200
Glyma07g13840.1 position (nts)

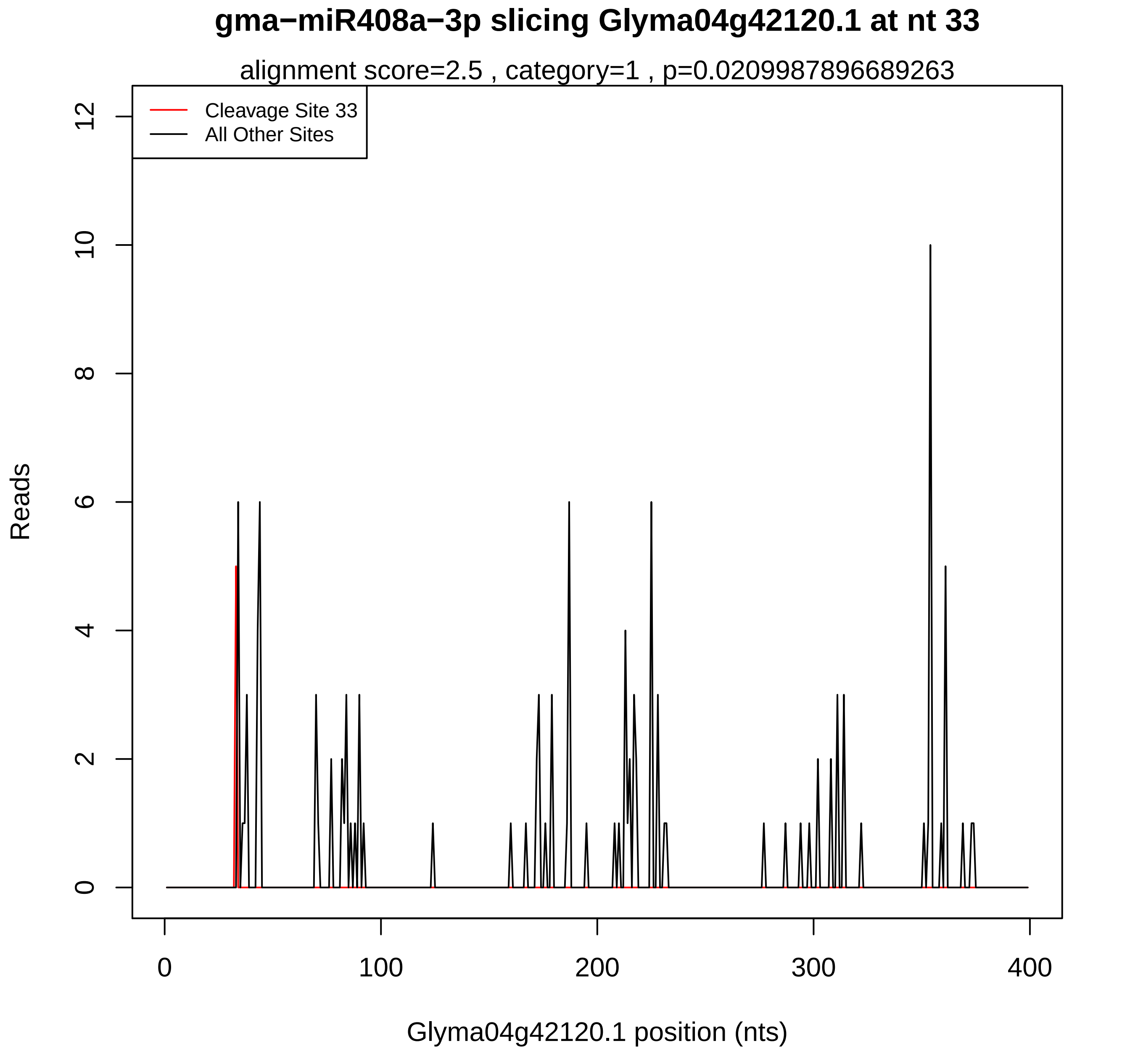

gma−miR408a−3p slicing Glyma04g42120.1 at nt 33
alignment score=2.5 , category=1 , p=0.0209987896689263
Cleavage Site 33
All Other Sites
Reads
0
2
4
6
8
10
12
0
100
200
300
400
Glyma04g42120.1 position (nts)

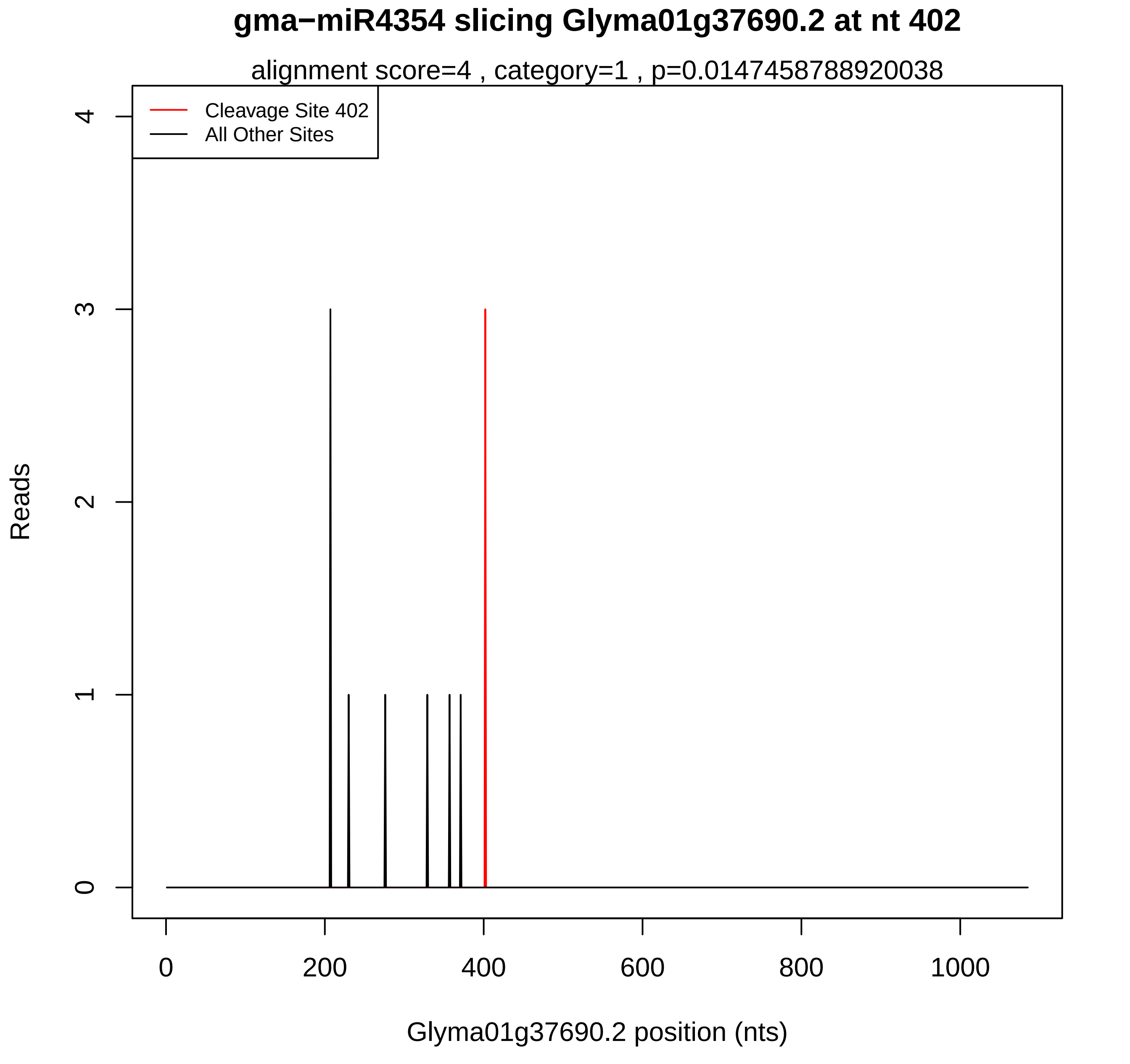

gma−miR4354 slicing Glyma01g37690.2 at nt 402
alignment score=4 , category=1 , p=0.0147458788920038
Cleavage Site 402
All Other Sites
Reads
0
1
2
3
4
0
200
400
600
800
1000
Glyma01g37690.2 position (nts)

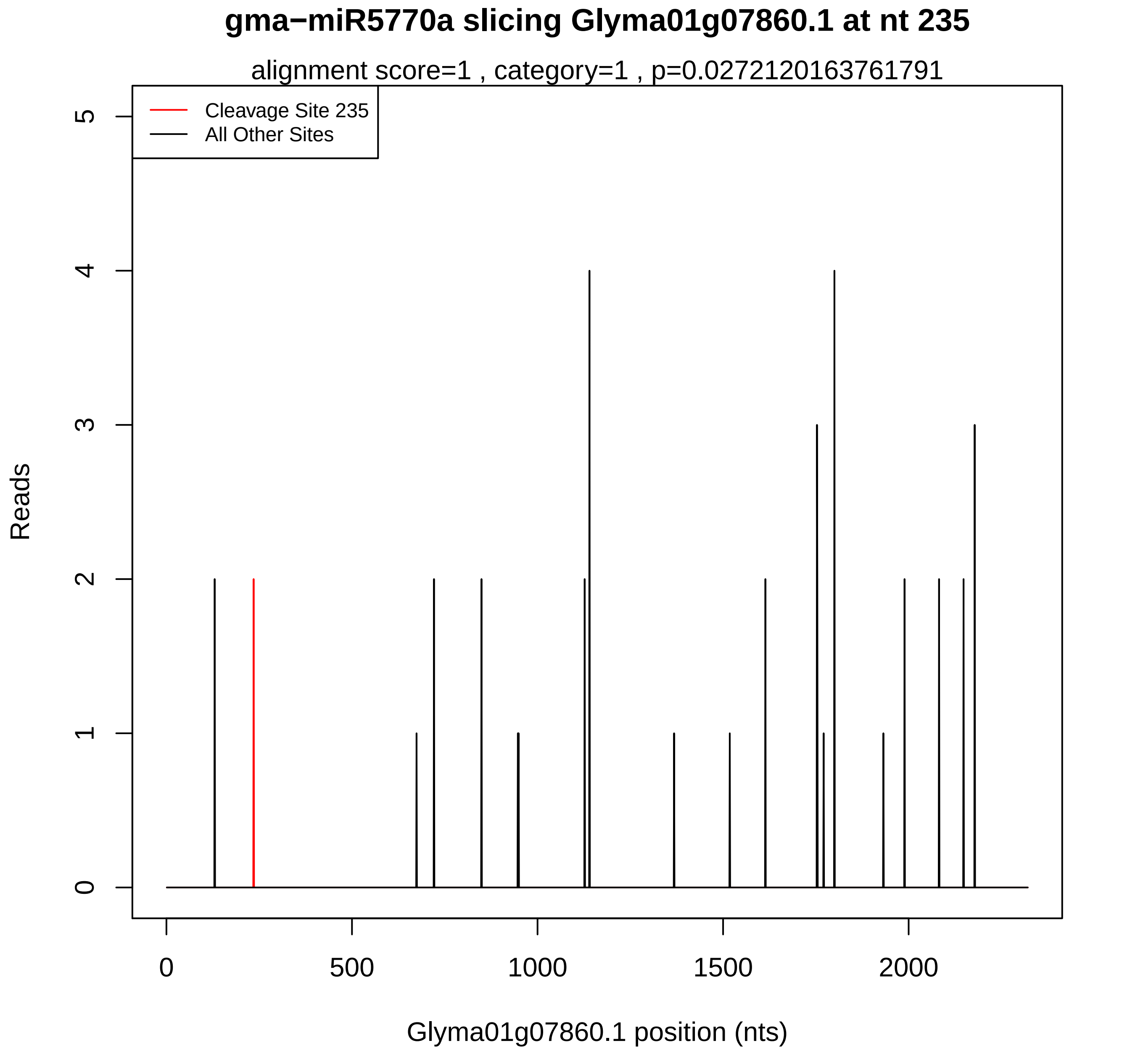

gma−miR5770a slicing Glyma01g07860.1 at nt 235
alignment score=1 , category=1 , p=0.0272120163761791
Cleavage Site 235
All Other Sites
Reads
0
1
2
3
4
5
0
500
1000
1500
2000
Glyma01g07860.1 position (nts)

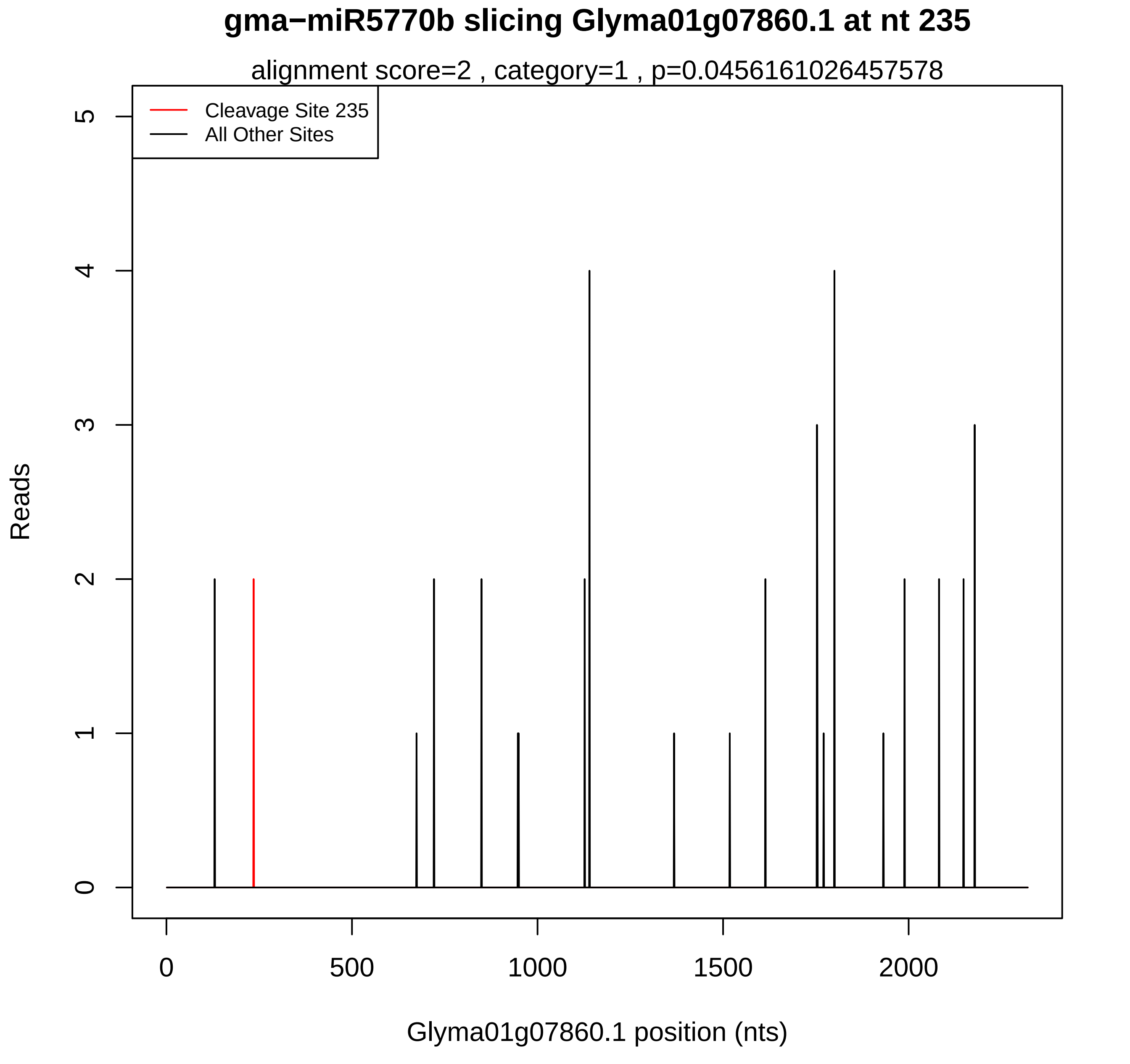

gma−miR5770b slicing Glyma01g07860.1 at nt 235
alignment score=2 , category=1 , p=0.0456161026457578
Cleavage Site 235
All Other Sites
Reads
0
1
2
3
4
5
0
500
1000
1500
2000
Glyma01g07860.1 position (nts)

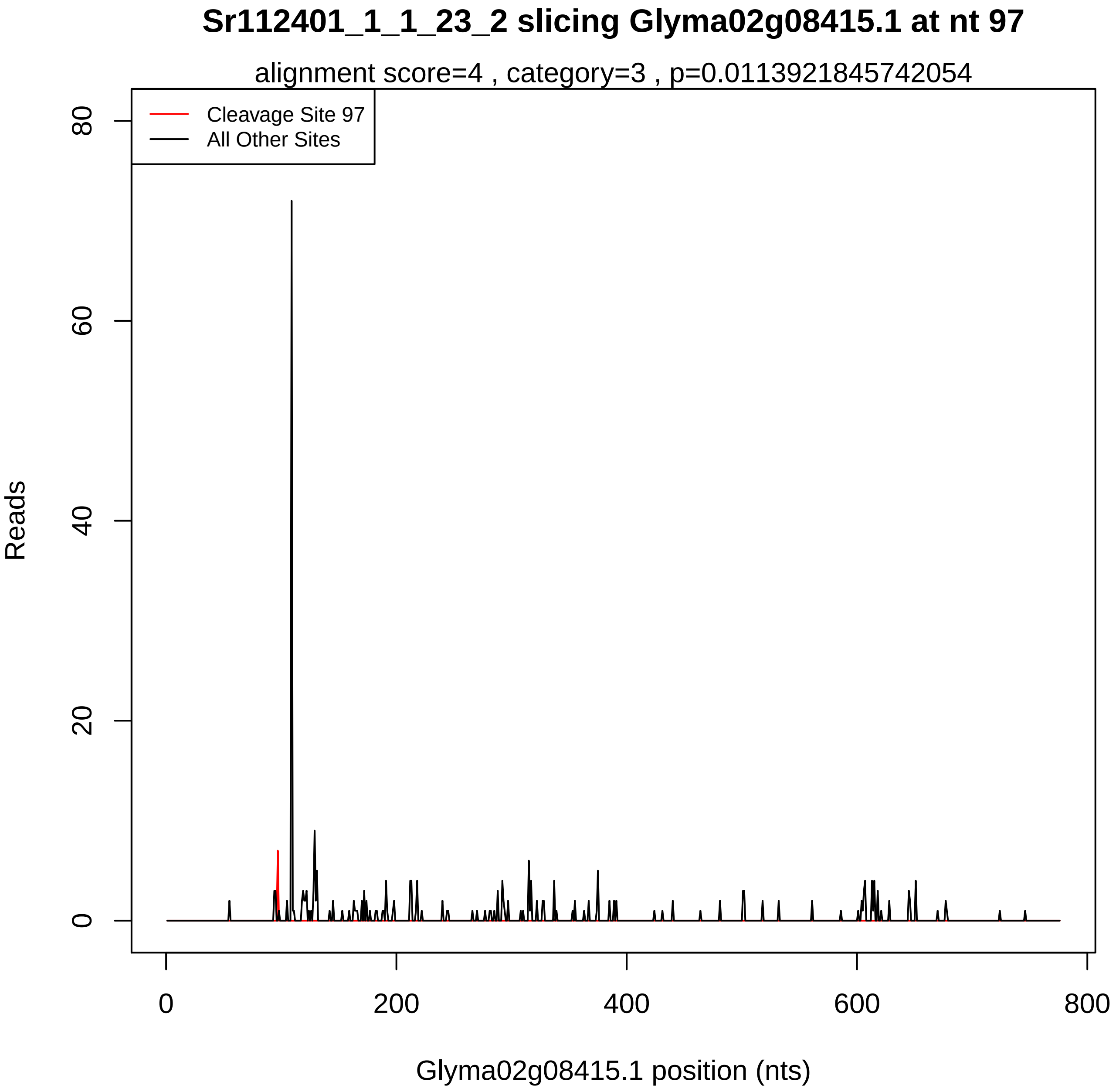
Sr112401_1_1_23_2 slicing Glyma02g08415.1 at nt 97
alignment score=4 , category=3 , p=0.0113921845742054
Cleavage Site 97
All Other Sites
Reads
0
20
40
60
80
0
200
400
600
800
Glyma02g08415.1 position (nts)

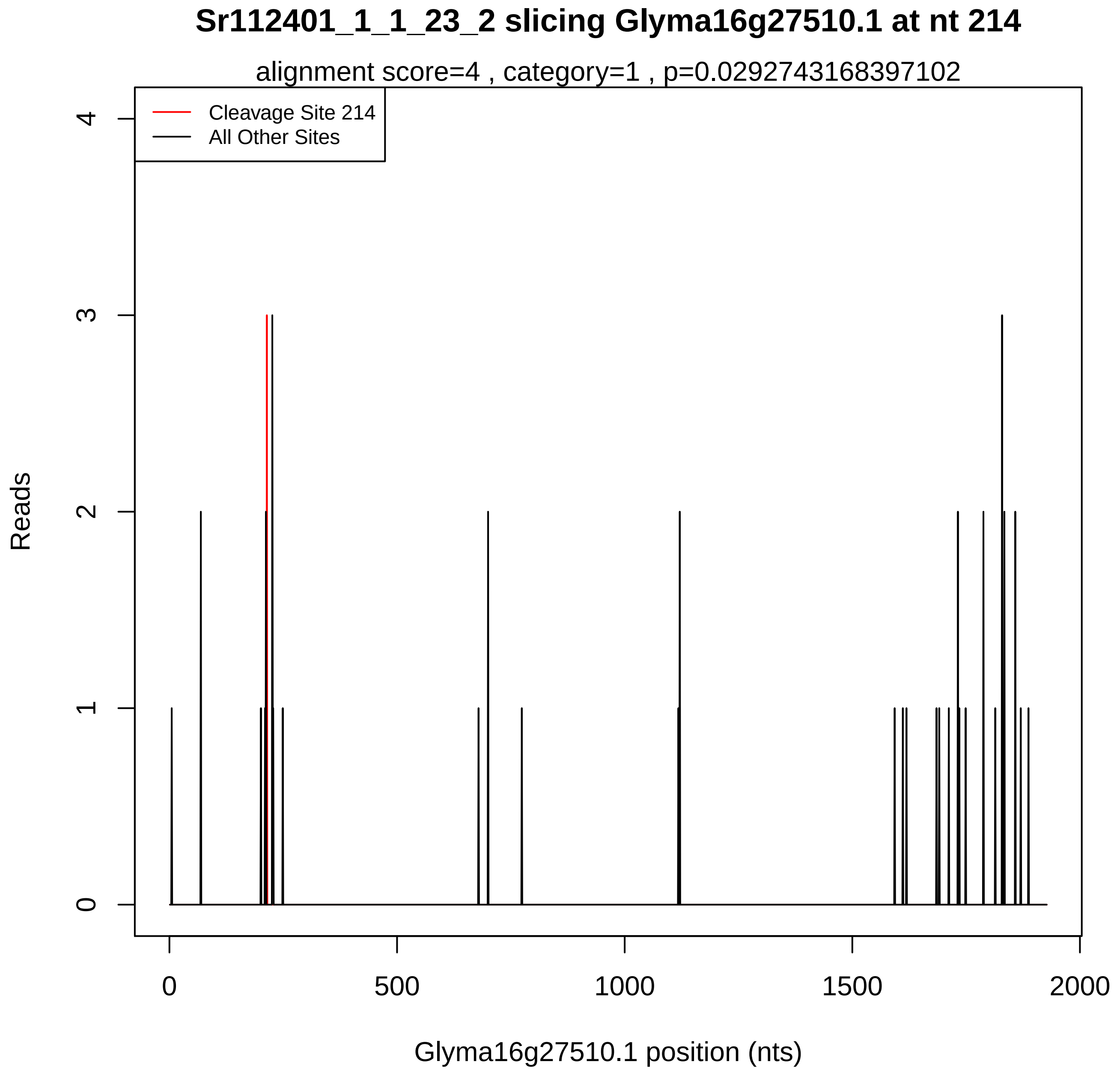
Sr112401_1_1_23_2 slicing Glyma16g27510.1 at nt 214
alignment score=4 , category=1 , p=0.0292743168397102
Cleavage Site 214
All Other Sites
Reads
0
1
2
3
4
0
500
1000
1500
2000
Glyma16g27510.1 position (nts)

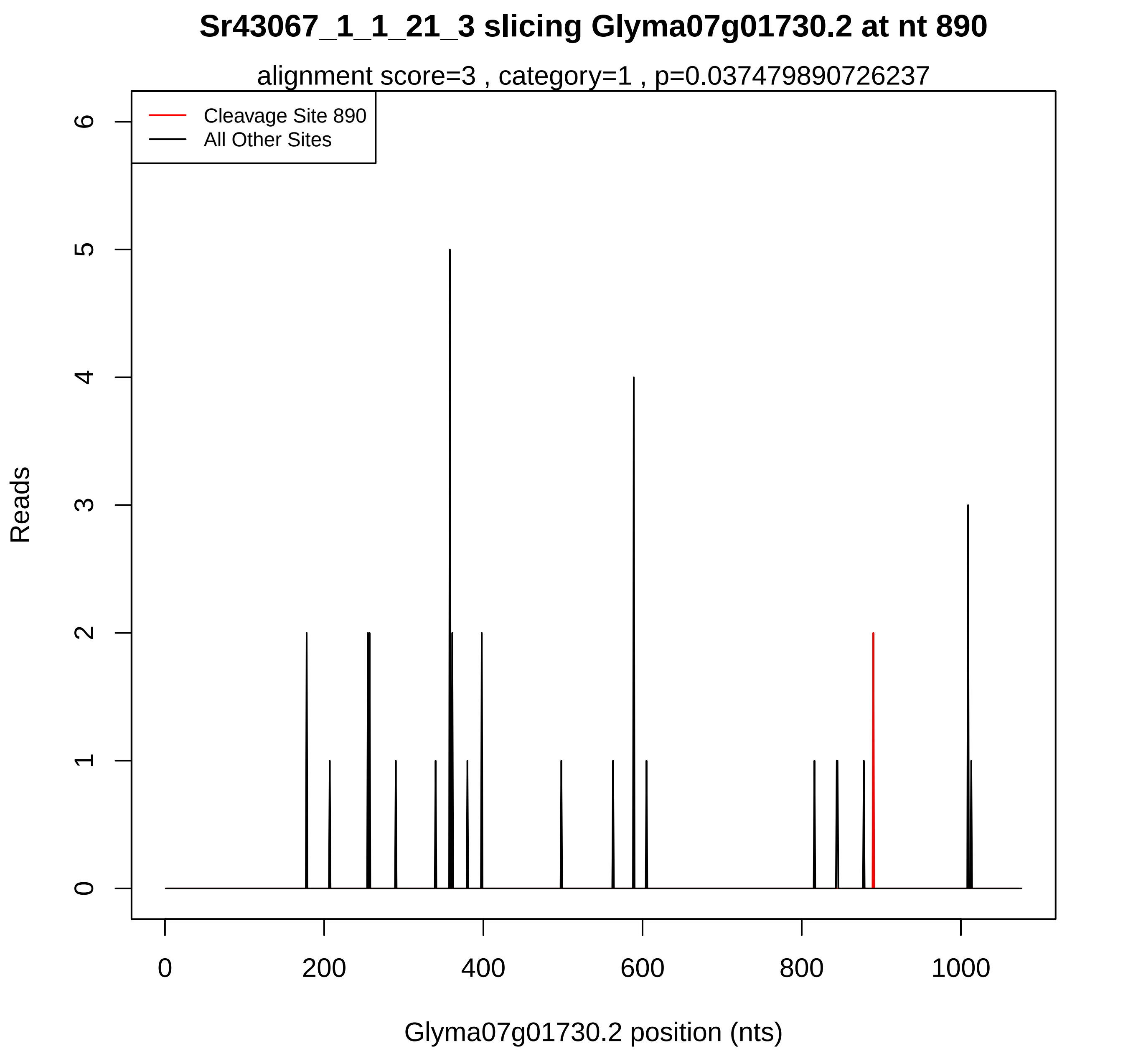

Sr43067_1_1_21_3 slicing Glyma07g01730.2 at nt 890
alignment score=3 , category=1 , p=0.037479890726237
Cleavage Site 890
All Other Sites
Reads
0
1
2
3
4
5
6
0
200
400
600
800
1000
Glyma07g01730.2 position (nts)

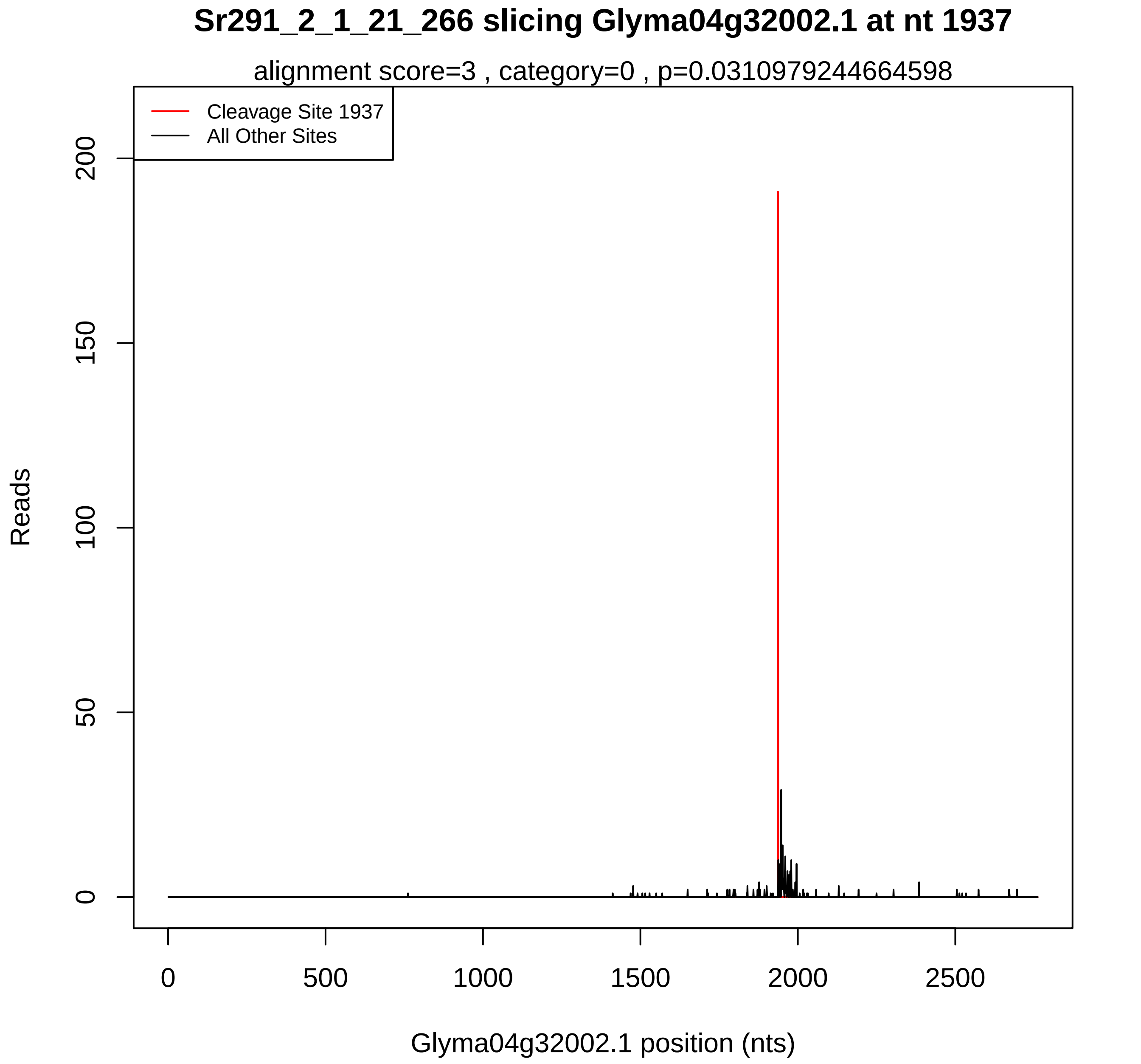

Sr291_2_1_21_266 slicing Glyma04g32002.1 at nt 1937
alignment score=3 , category=0 , p=0.0310979244664598
Cleavage Site 1937
All Other Sites
Reads
0
50
100
150
200
0
500
1000
1500
2000
2500
Glyma04g32002.1 position (nts)

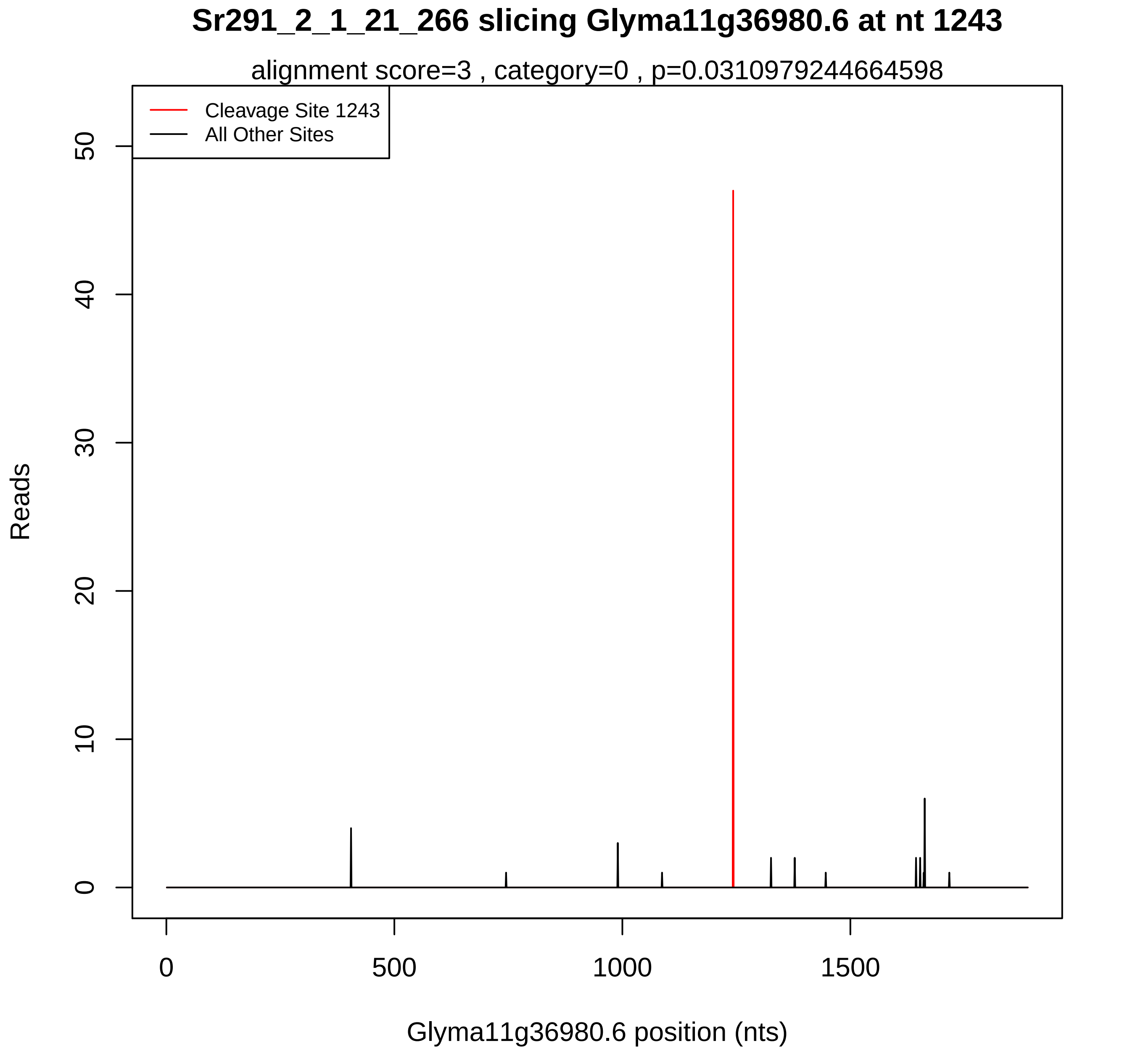
Sr291_2_1_21_266 slicing Glyma11g36980.6 at nt 1243
alignment score=3 , category=0 , p=0.0310979244664598
Cleavage Site 1243
All Other Sites
Reads
0
10
20
30
40
50
0
500
1000
1500
Glyma11g36980.6 position (nts)

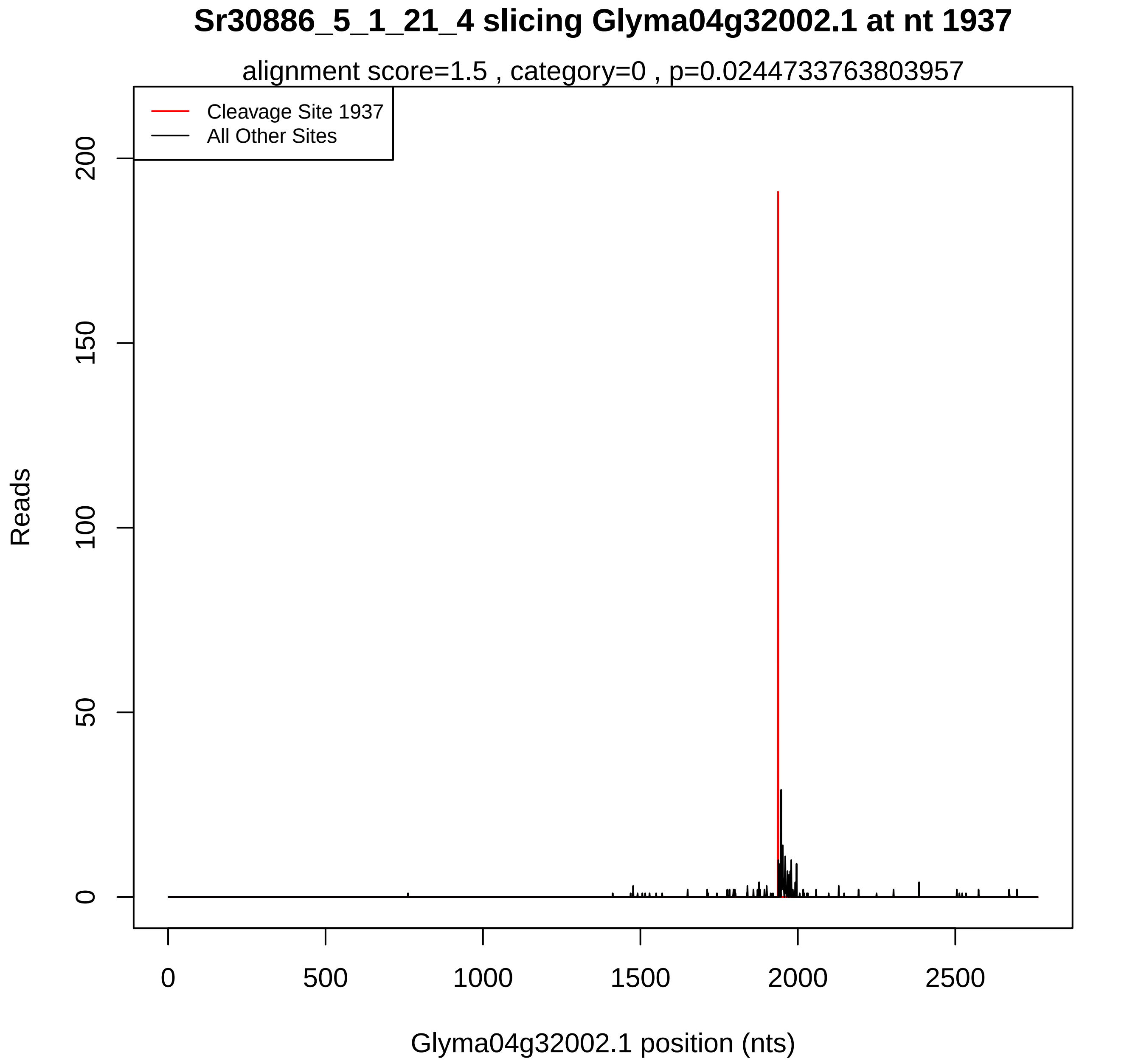
Sr30886_5_1_21_4 slicing Glyma04g32002.1 at nt 1937
alignment score=1.5 , category=0 , p=0.0244733763803957
Cleavage Site 1937
All Other Sites
Reads
0
50
100
150
200
0
500
1000
1500
2000
2500
Glyma04g32002.1 position (nts)

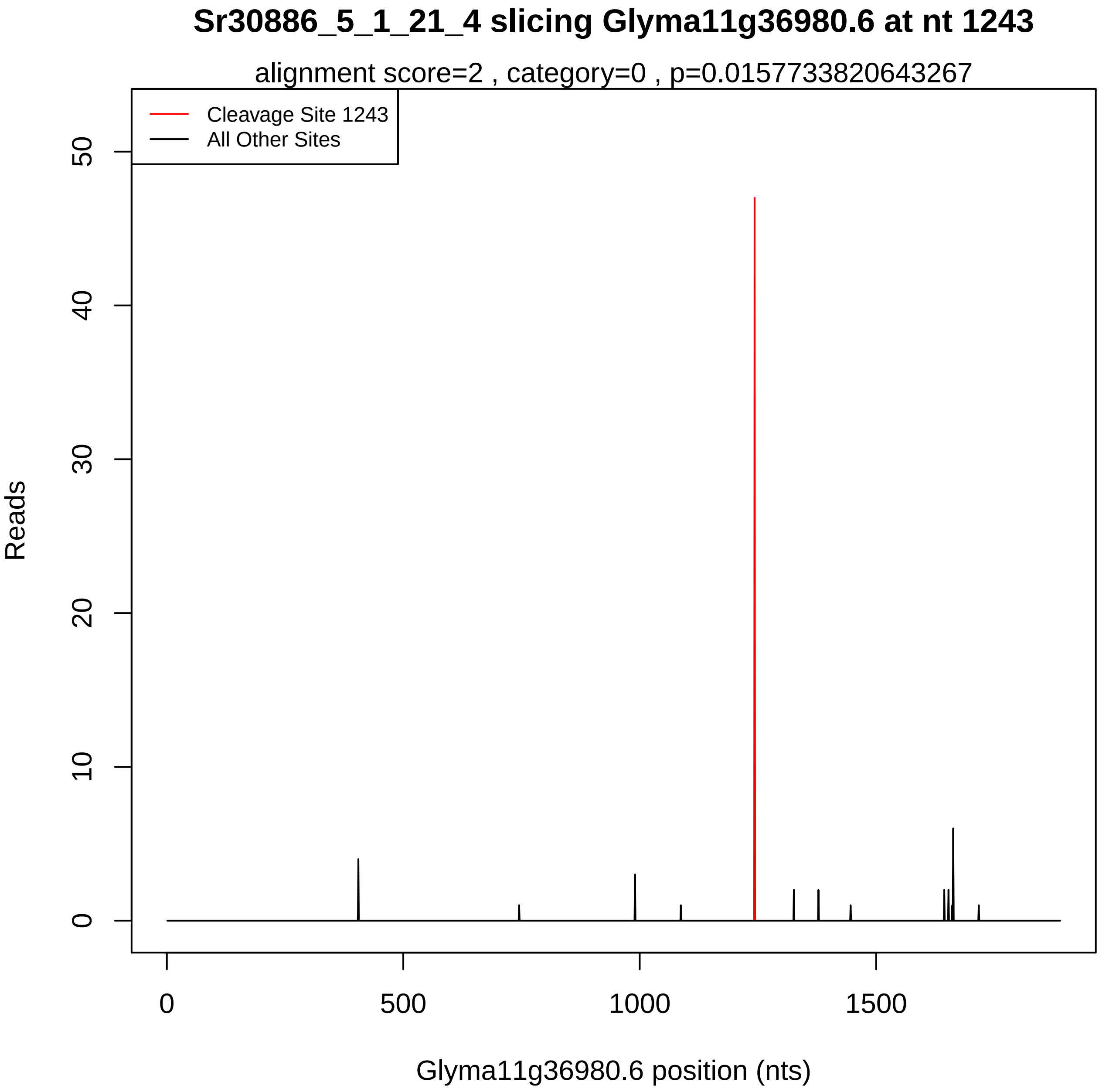

Sr30886_5_1_21_4 slicing Glyma11g36980.6 at nt 1243
alignment score=2 , category=0 , p=0.0157733820643267
Cleavage Site 1243
All Other Sites
Reads
0
10
20
30
40
50
0
500
1000
1500
Glyma11g36980.6 position (nts)

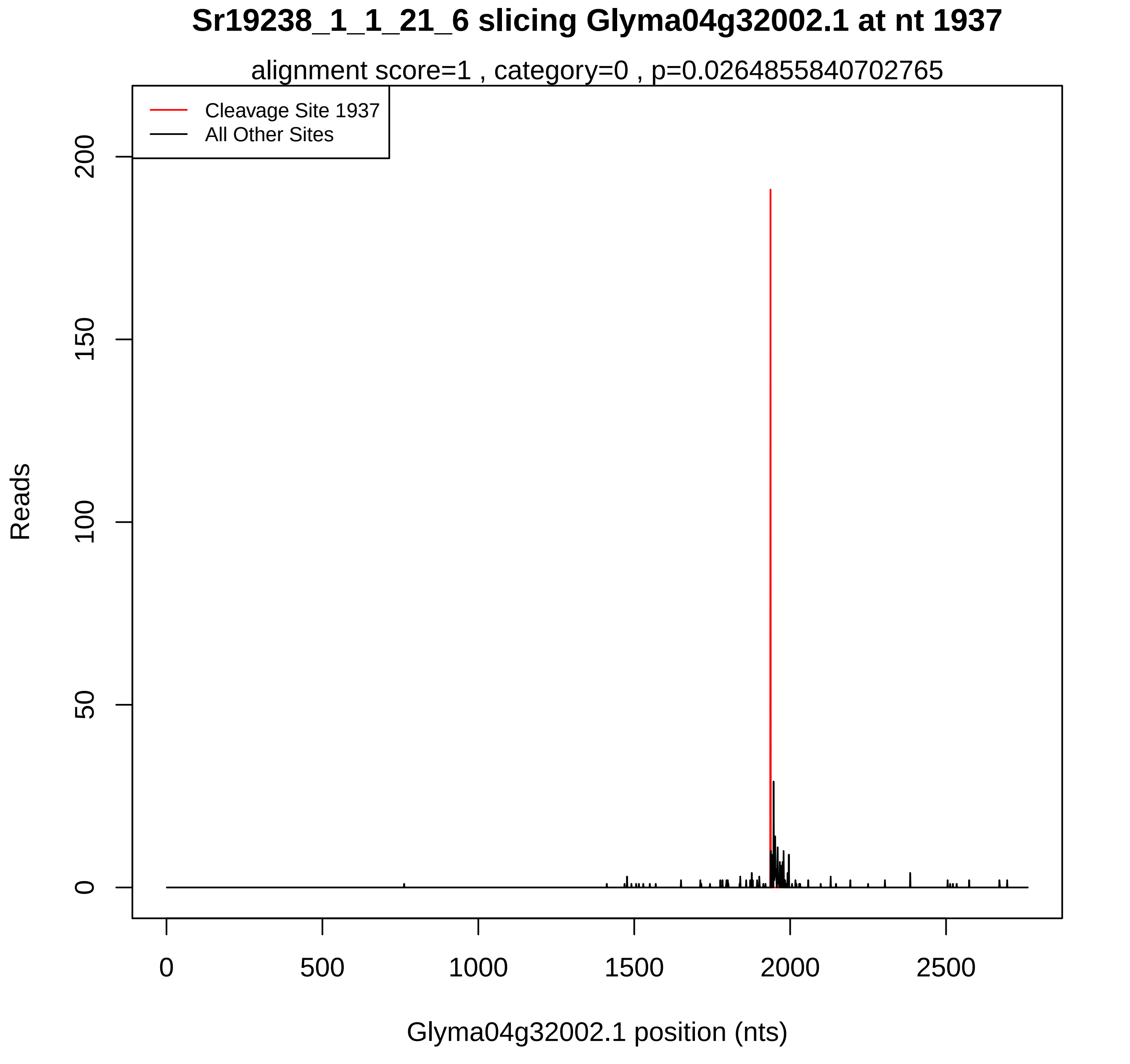

Sr19238_1_1_21_6 slicing Glyma04g32002.1 at nt 1937
alignment score=1 , category=0 , p=0.0264855840702765
Cleavage Site 1937
All Other Sites
Reads
0
50
100
150
200
0
500
1000
1500
2000
2500
Glyma04g32002.1 position (nts)

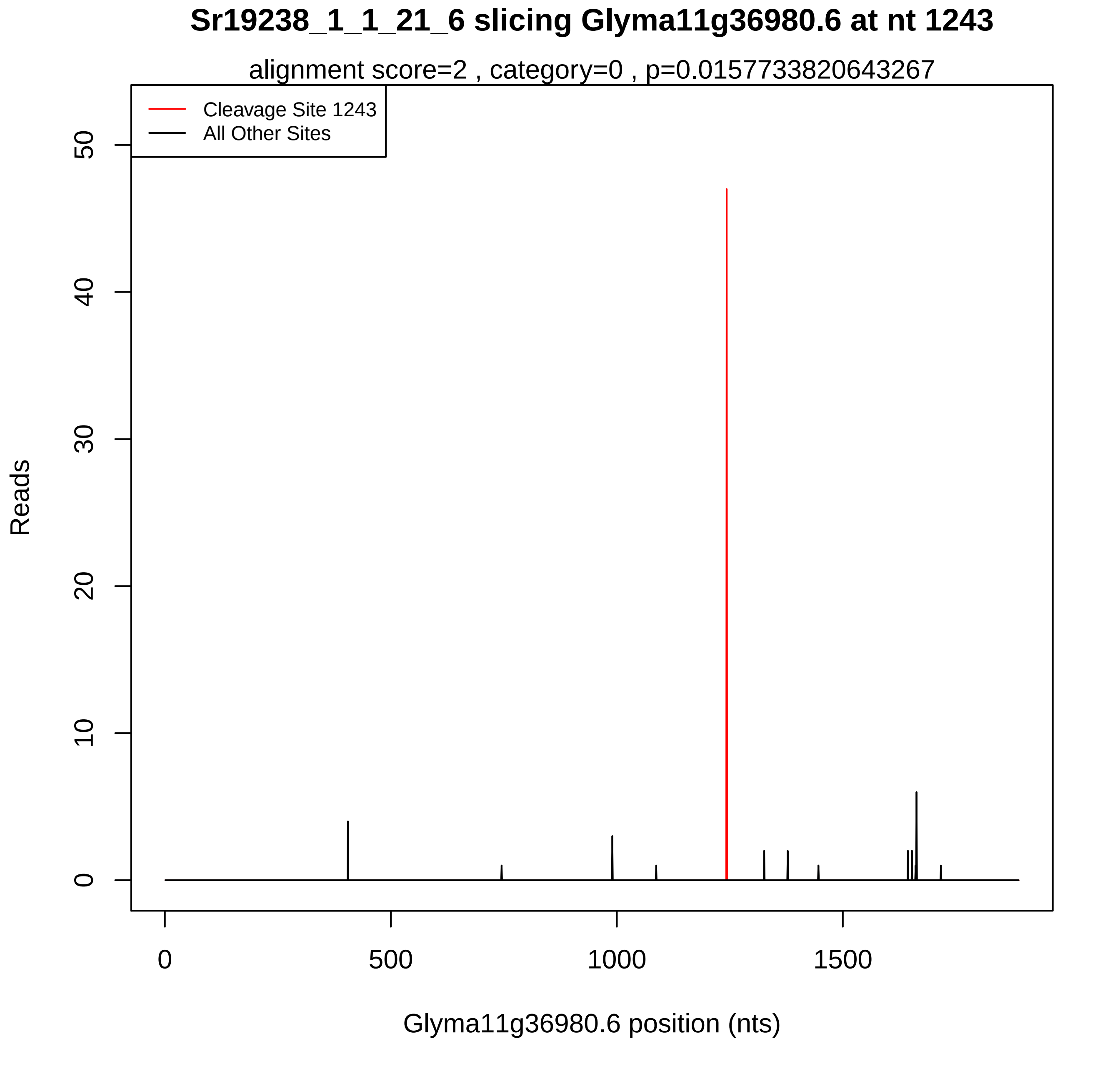
Sr19238_1_1_21_6 slicing Glyma11g36980.6 at nt 1243
alignment score=2 , category=0 , p=0.0157733820643267
Cleavage Site 1243
All Other Sites
Reads
0
10
20
30
40
50
0
500
1000
1500
Glyma11g36980.6 position (nts)

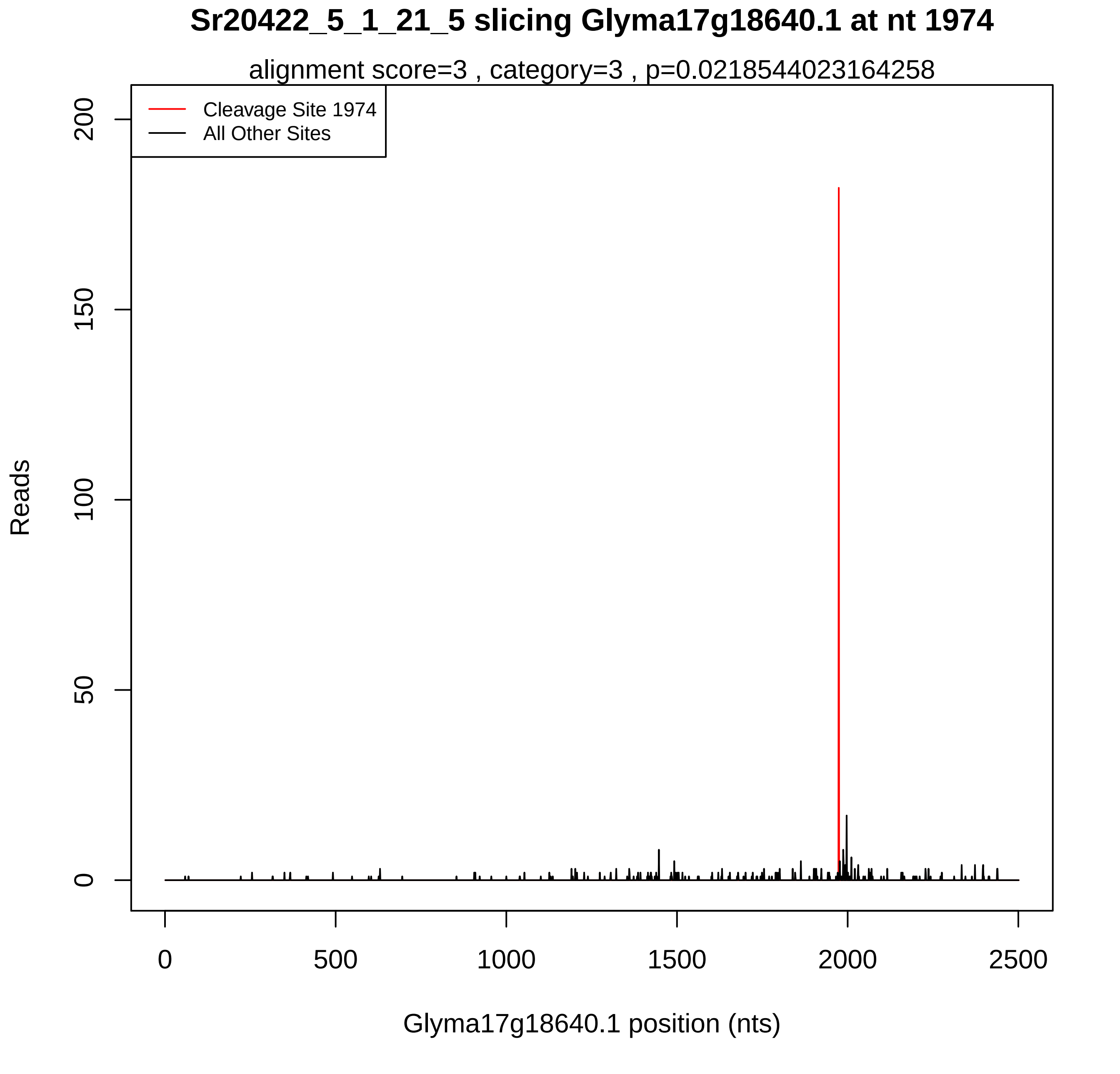

Sr20422_5_1_21_5 slicing Glyma17g18640.1 at nt 1974
alignment score=3 , category=3 , p=0.0218544023164258
Cleavage Site 1974
All Other Sites
Reads
0
50
100
150
200
0
500
1000
1500
2000
2500
Glyma17g18640.1 position (nts)

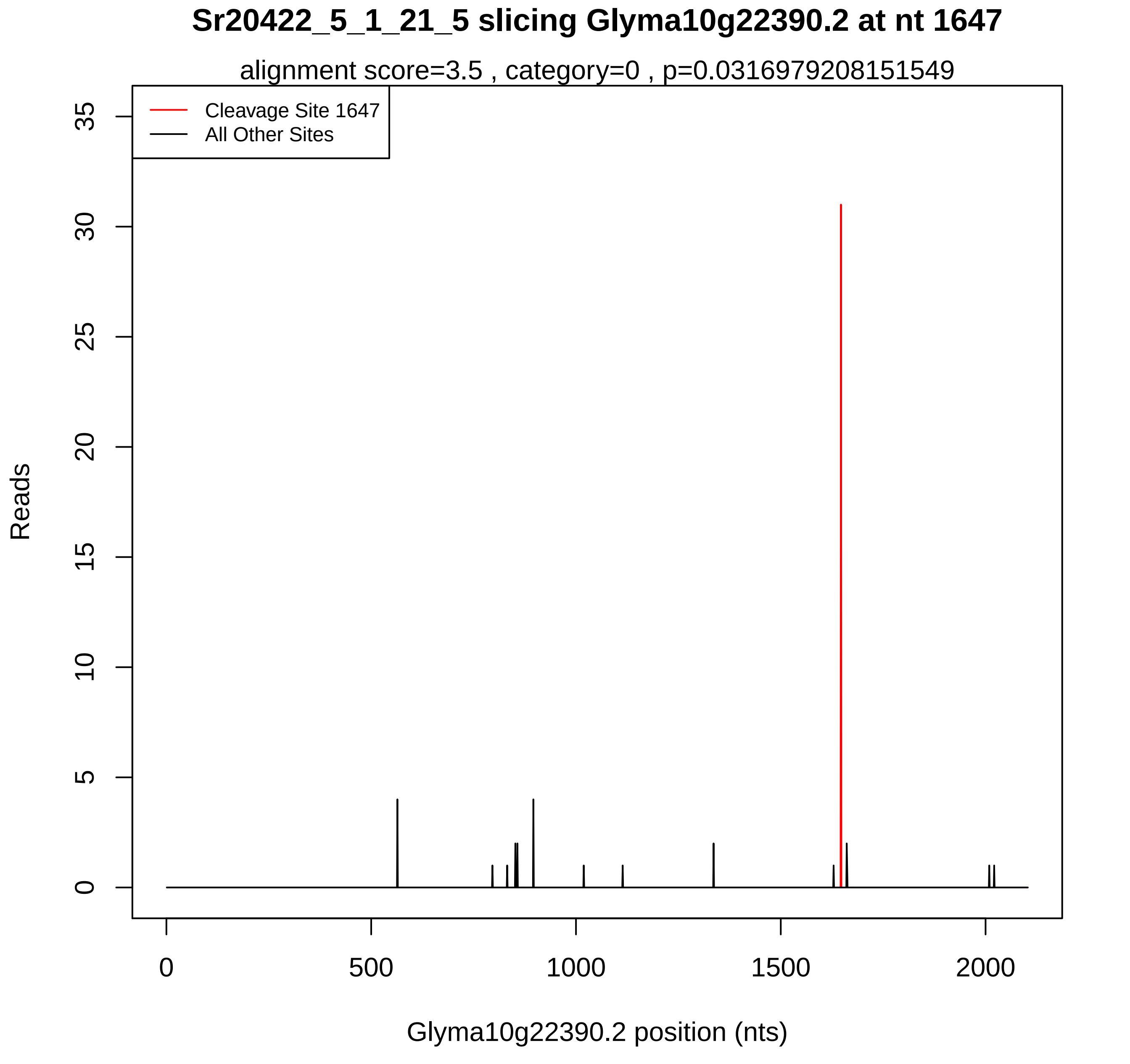

Sr20422_5_1_21_5 slicing Glyma10g22390.2 at nt 1647
alignment score=3.5 , category=0 , p=0.0316979208151549
Cleavage Site 1647
All Other Sites
Reads
0
5
10
15
20
25
30
35
0
500
1000
1500
2000
Glyma10g22390.2 position (nts)

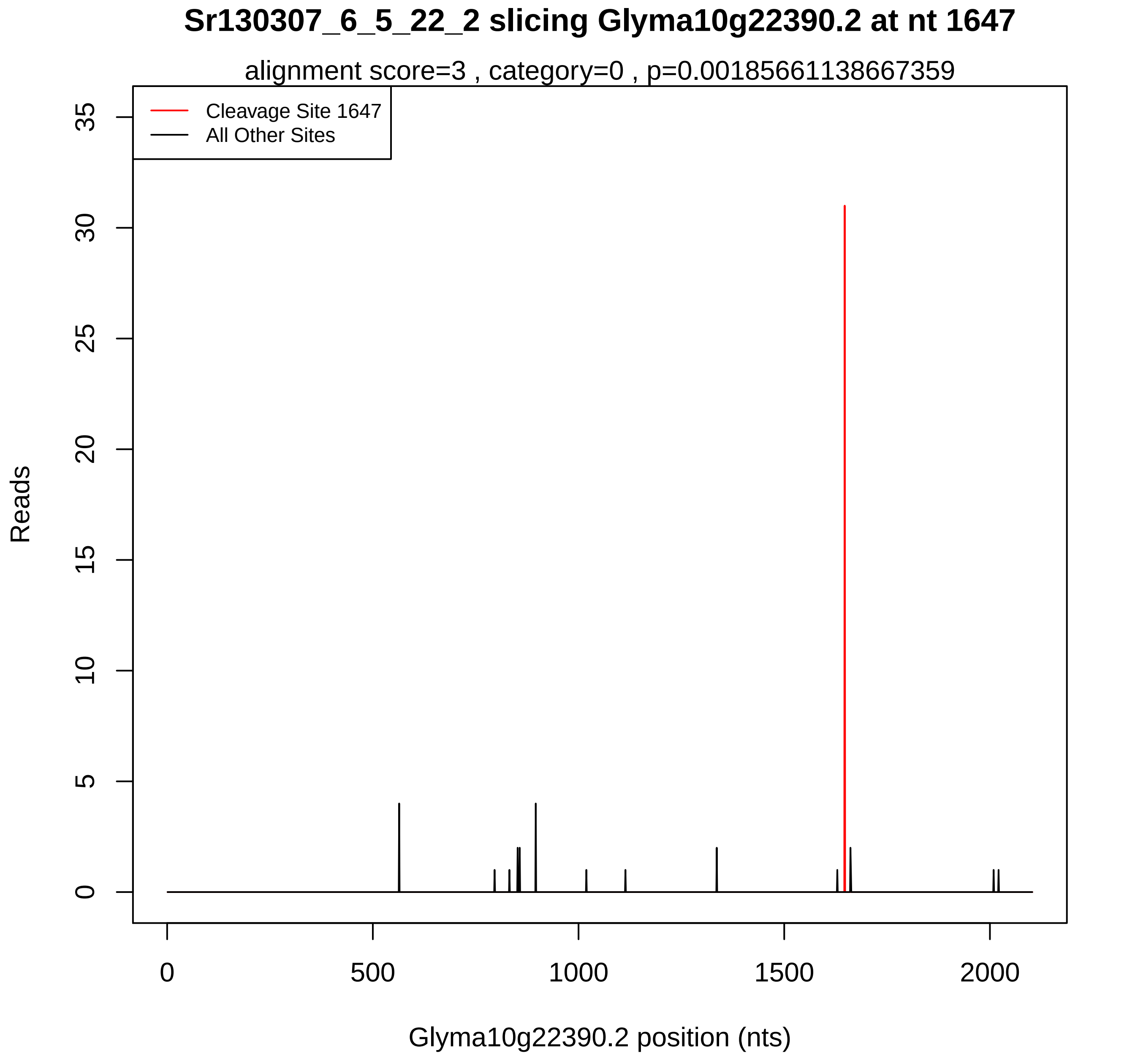

Sr130307_6_5_22_2 slicing Glyma10g22390.2 at nt 1647
alignment score=3 , category=0 , p=0.00185661138667359
Cleavage Site 1647
All Other Sites
Reads
0
5
10
15
20
25
30
35
0
500
1000
1500
2000
Glyma10g22390.2 position (nts)

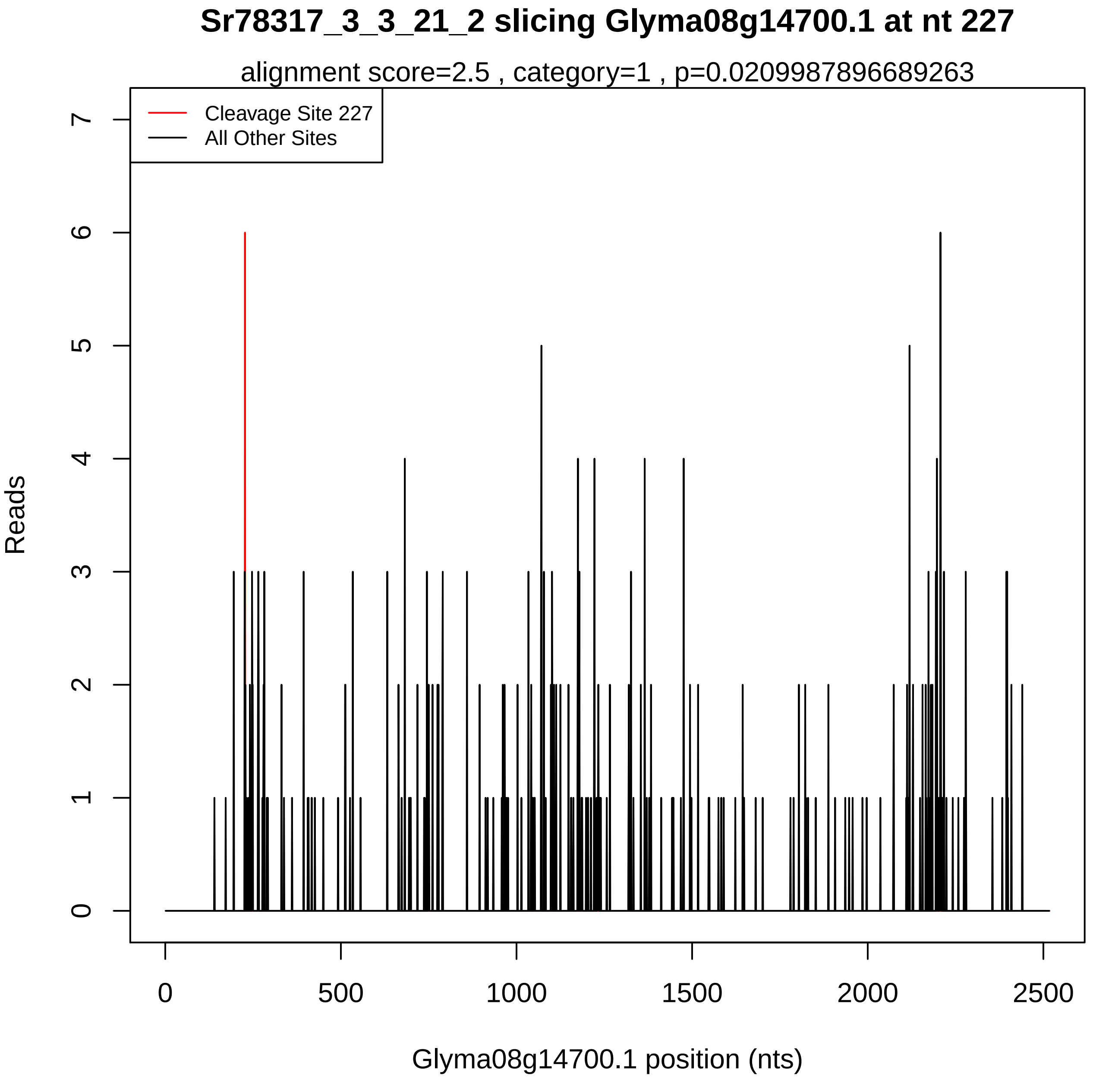
Sr78317_3_3_21_2 slicing Glyma08g14700.1 at nt 227
alignment score=2.5 , category=1 , p=0.0209987896689263
Cleavage Site 227
All Other Sites
Reads
0
1
2
3
4
5
6
7
0
500
1000
1500
2000
2500
Glyma08g14700.1 position (nts)

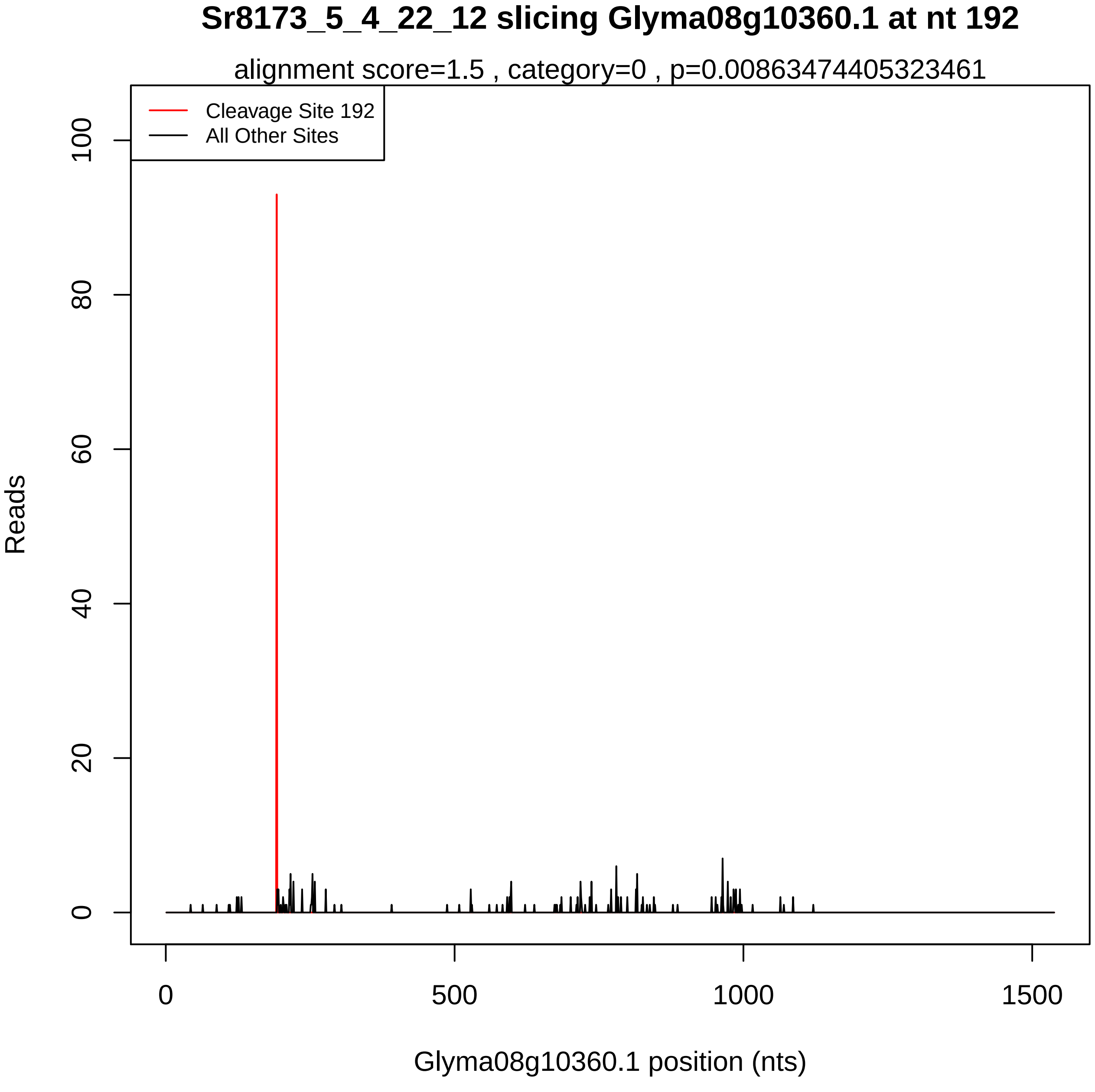

Sr8173_5_4_22_12 slicing Glyma08g10360.1 at nt 192
alignment score=1.5 , category=0 , p=0.00863474405323461
Cleavage Site 192
All Other Sites
Reads
0
20
40
60
80
100
0
500
1000
1500
Glyma08g10360.1 position (nts)

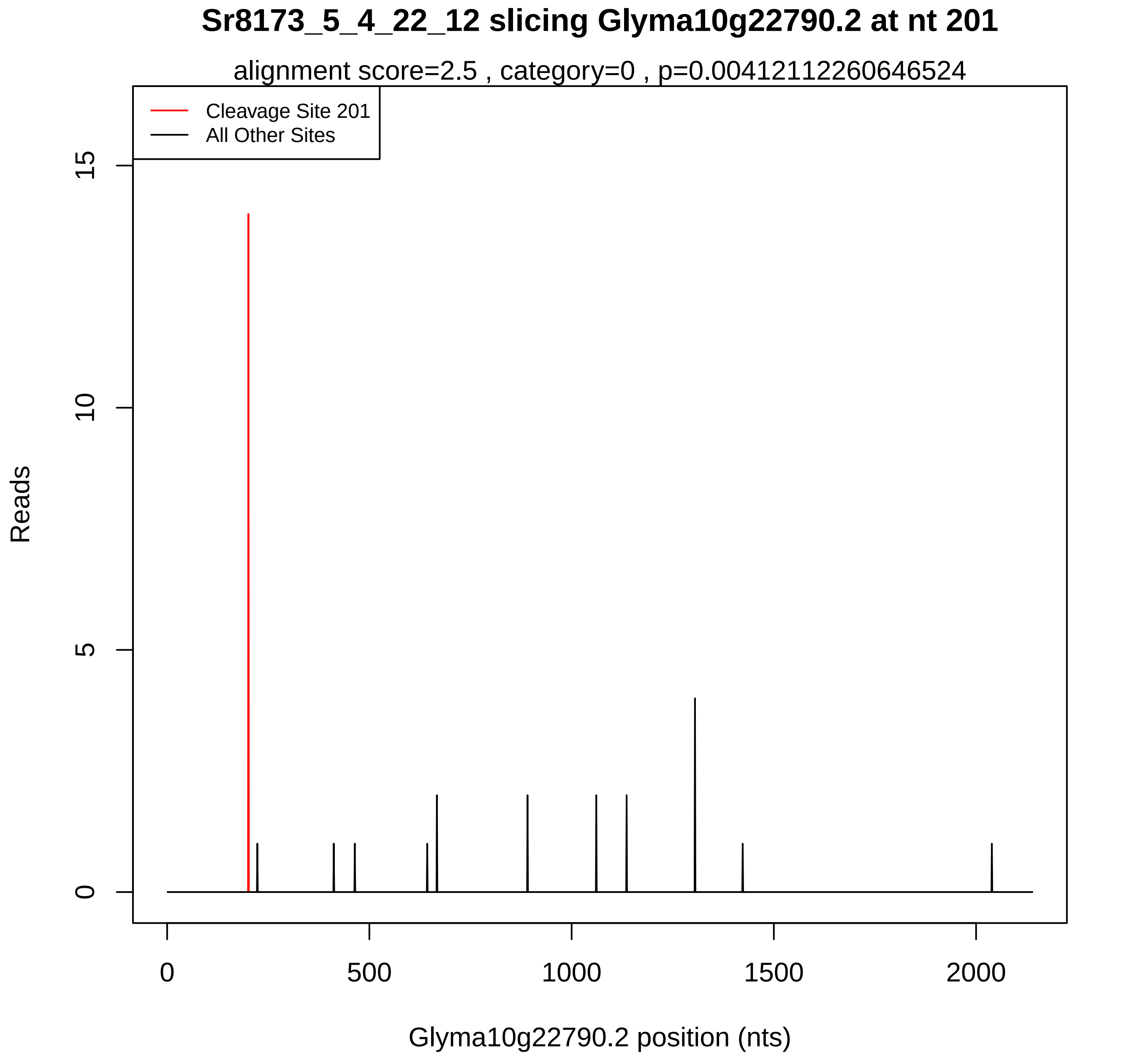

Sr8173_5_4_22_12 slicing Glyma10g22790.2 at nt 201
alignment score=2.5 , category=0 , p=0.00412112260646524
Cleavage Site 201
All Other Sites
Reads
0
5
10
15
0
500
1000
1500
2000
Glyma10g22790.2 position (nts)

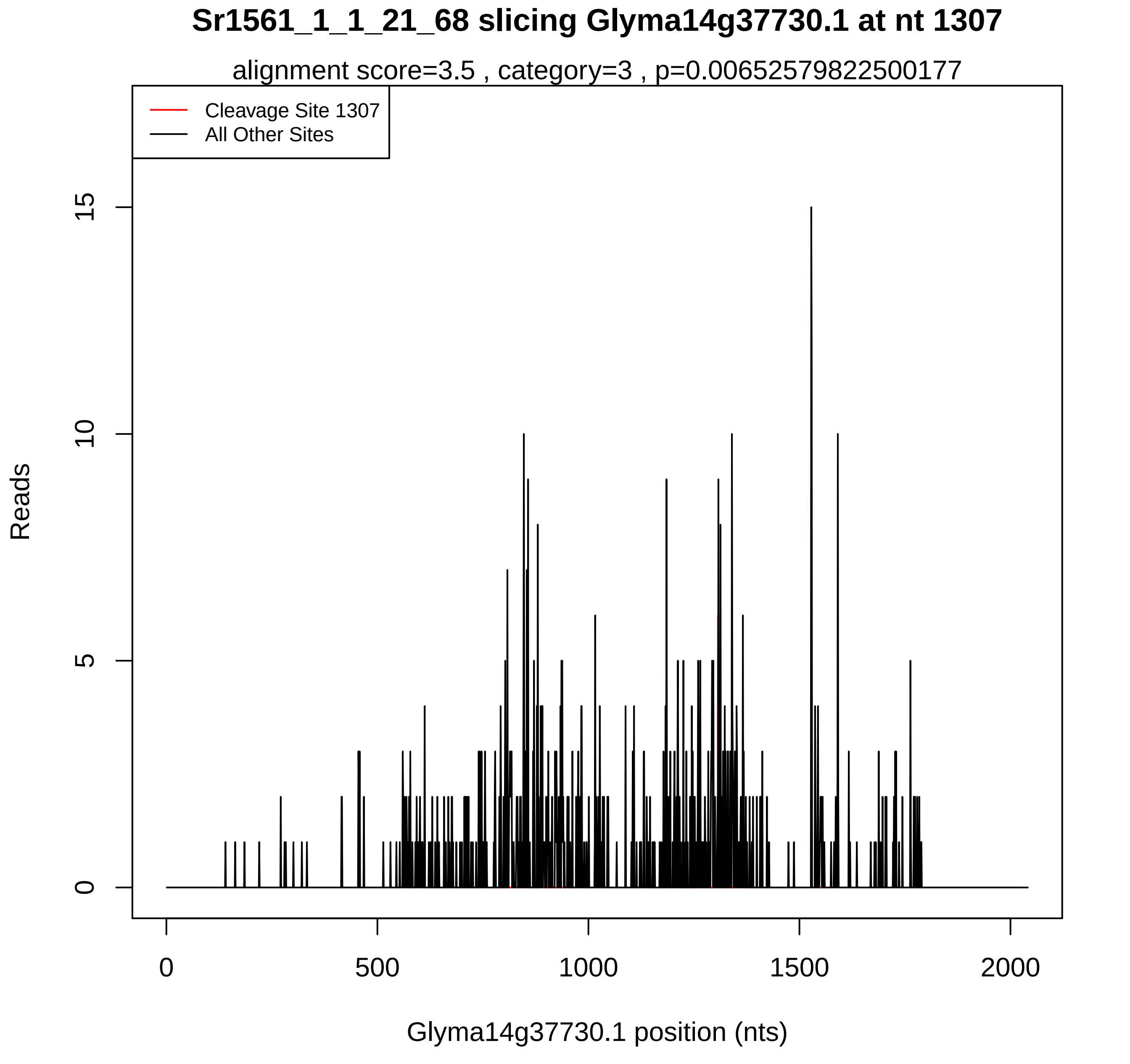

Sr1561_1_1_21_68 slicing Glyma14g37730.1 at nt 1307
alignment score=3.5 , category=3 , p=0.00652579822500177
Cleavage Site 1307
All Other Sites
Reads
0
5
10
15
0
500
1000
1500
2000
Glyma14g37730.1 position (nts)

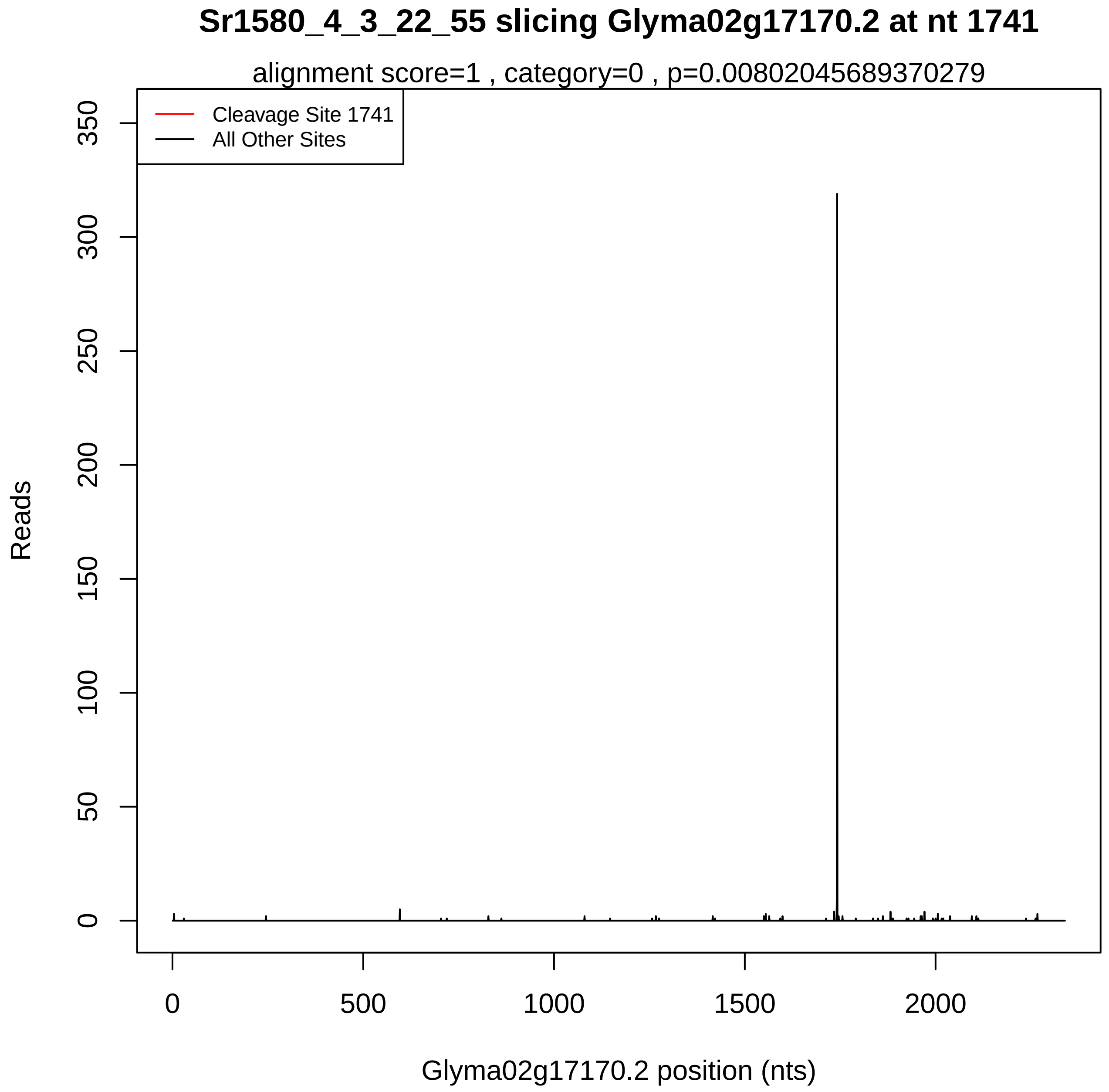

Sr1580_4_3_22_55 slicing Glyma02g17170.2 at nt 1741
alignment score=1 , category=0 , p=0.00802045689370279
Cleavage Site 1741
All Other Sites
Reads
0
50
100
150
200
250
300
350
0
500
1000
1500
2000
Glyma02g17170.2 position (nts)

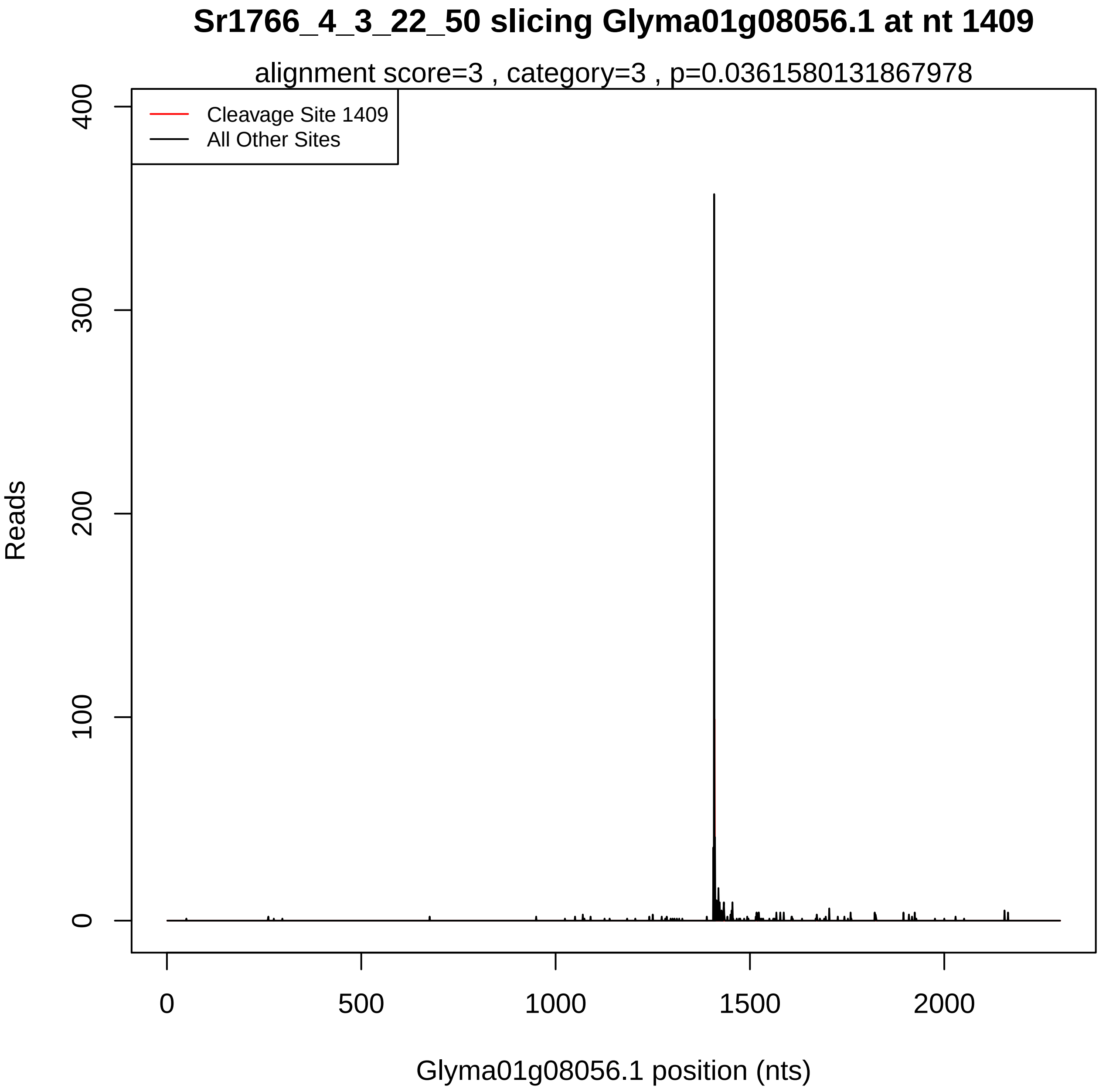

Sr1766_4_3_22_50 slicing Glyma01g08056.1 at nt 1409
alignment score=3 , category=3 , p=0.0361580131867978
Cleavage Site 1409
All Other Sites
Reads
0
100
200
300
400
0
500
1000
1500
2000
Glyma01g08056.1 position (nts)

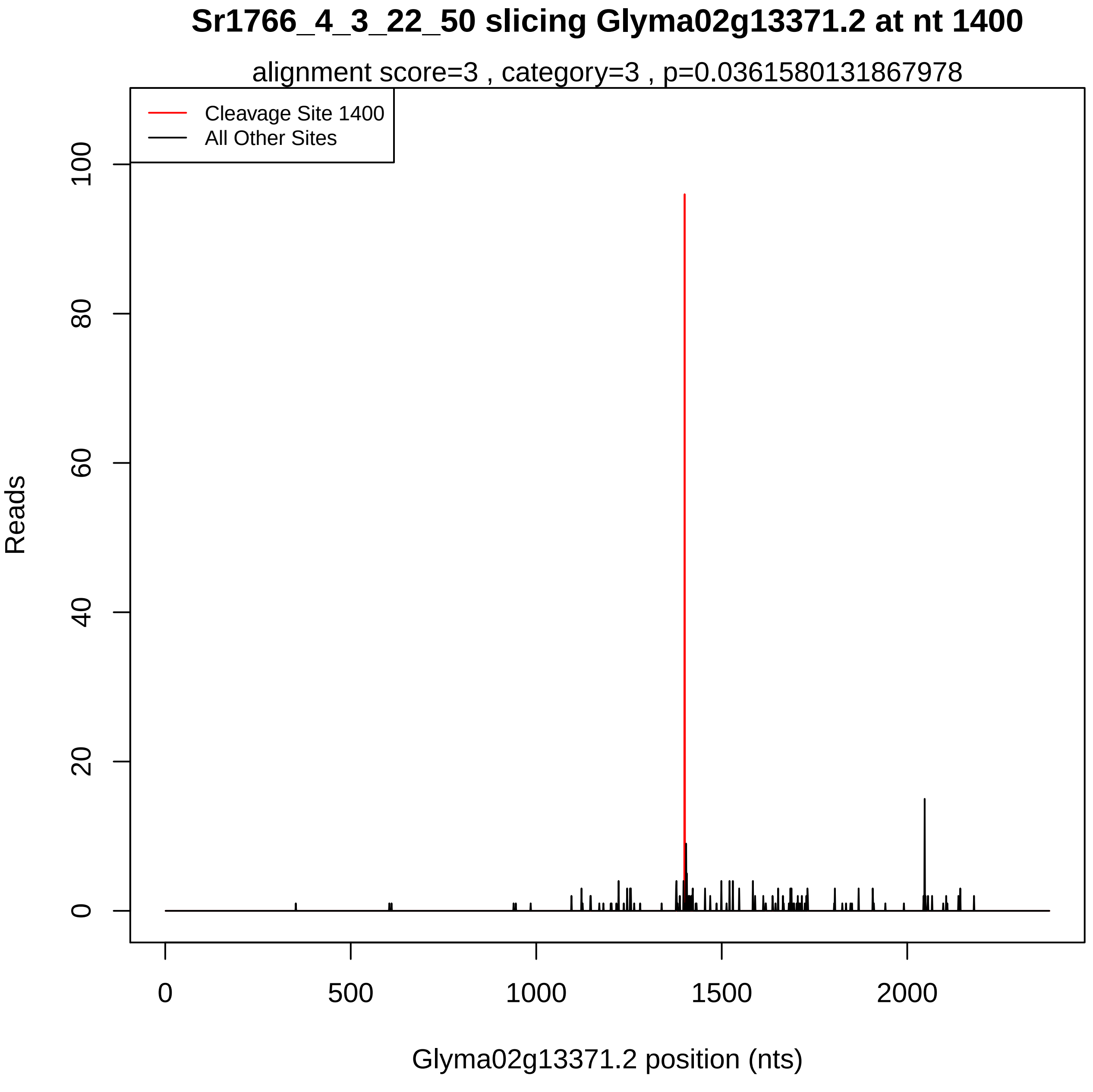

Sr1766_4_3_22_50 slicing Glyma02g13371.2 at nt 1400
alignment score=3 , category=3 , p=0.0361580131867978
Cleavage Site 1400
All Other Sites
Reads
0
20
40
60
80
100
0
500
1000
1500
2000
Glyma02g13371.2 position (nts)

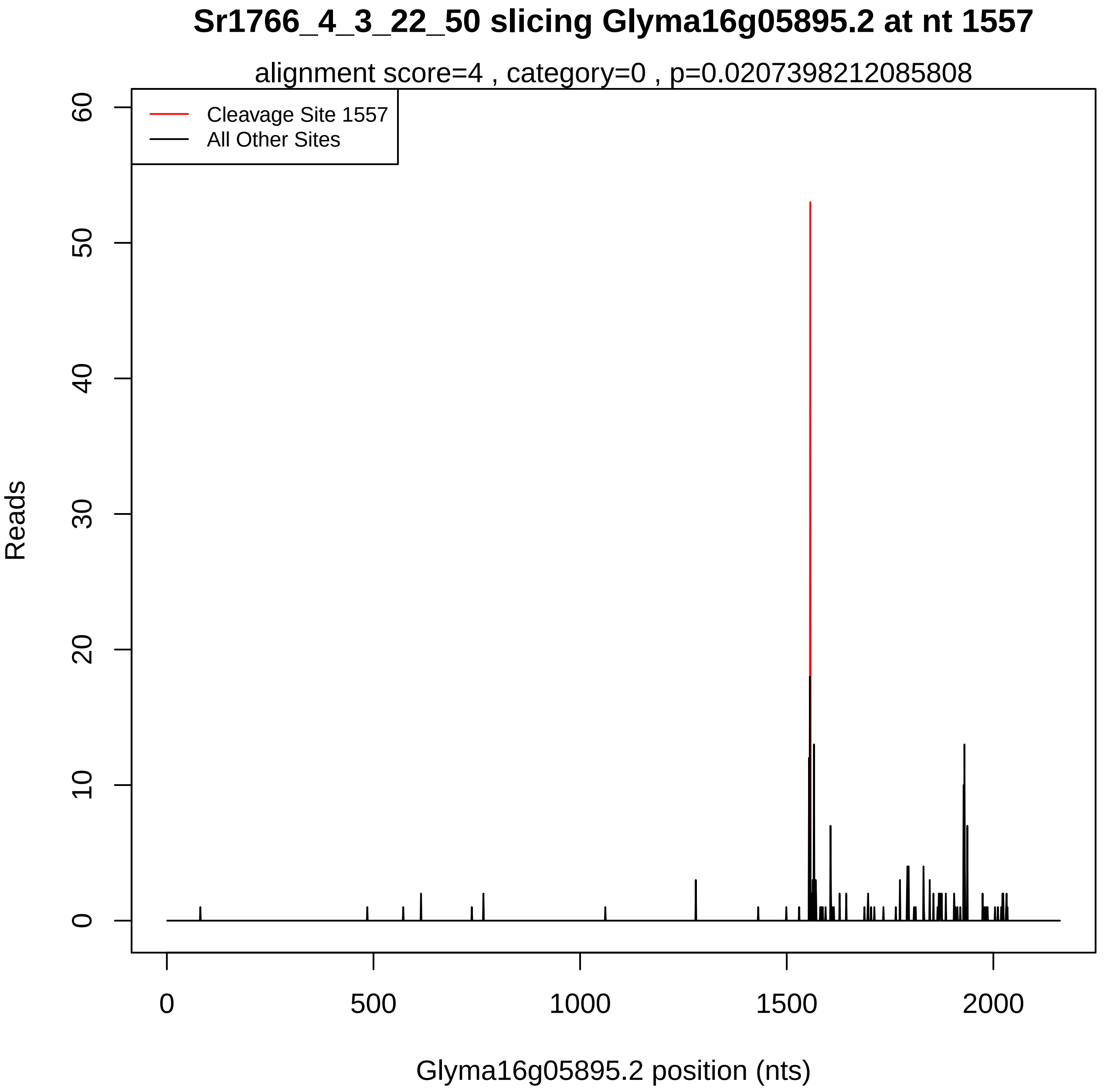

Sr1766_4_3_22_50 slicing Glyma16g05895.2 at nt 1557
alignment score=4 , category=0 , p=0.0207398212085808
Cleavage Site 1557
All Other Sites
Reads
0
10
20
30
40
50
60
0
500
1000
1500
2000
Glyma16g05895.2 position (nts)

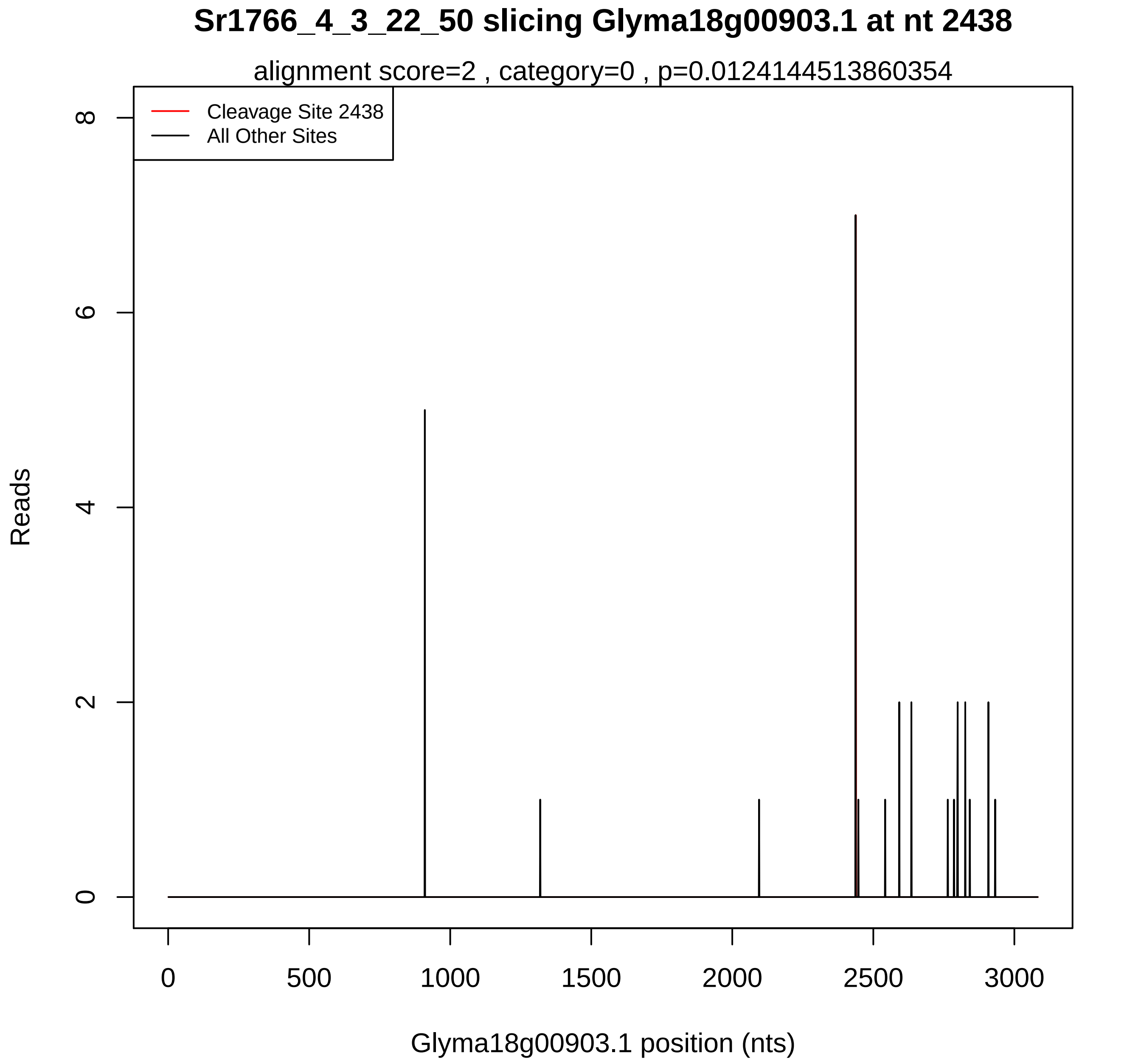

Sr1766_4_3_22_50 slicing Glyma18g00903.1 at nt 2438
alignment score=2 , category=0 , p=0.0124144513860354
Cleavage Site 2438
All Other Sites
Reads
0
2
4
6
8
0
500
1000
1500
2000
2500
3000
Glyma18g00903.1 position (nts)

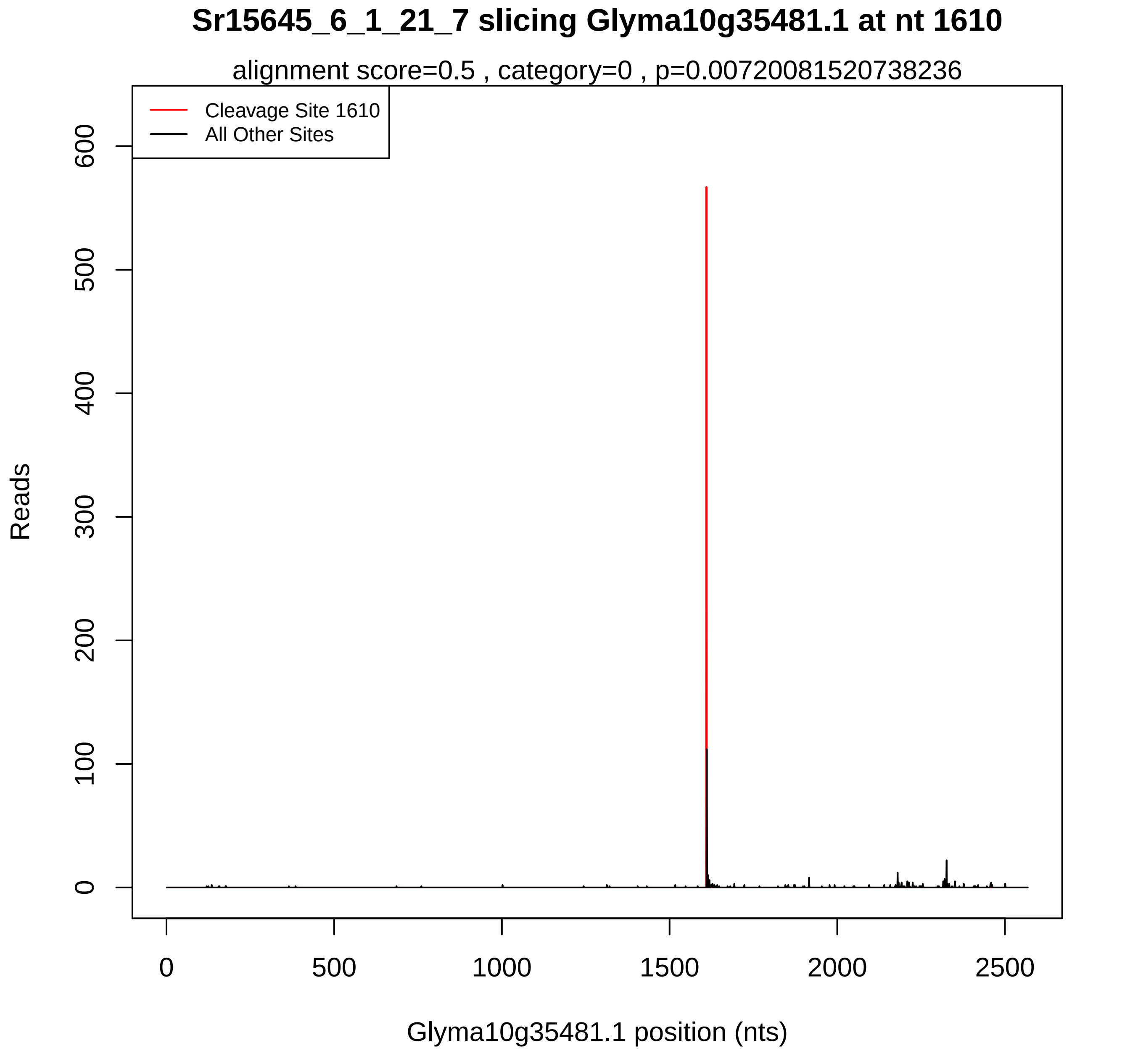

Sr15645_6_1_21_7 slicing Glyma10g35481.1 at nt 1610
alignment score=0.5 , category=0 , p=0.00720081520738236
Cleavage Site 1610
All Other Sites
Reads
0
100
200
300
400
500
600
0
500
1000
1500
2000
2500
Glyma10g35481.1 position (nts)

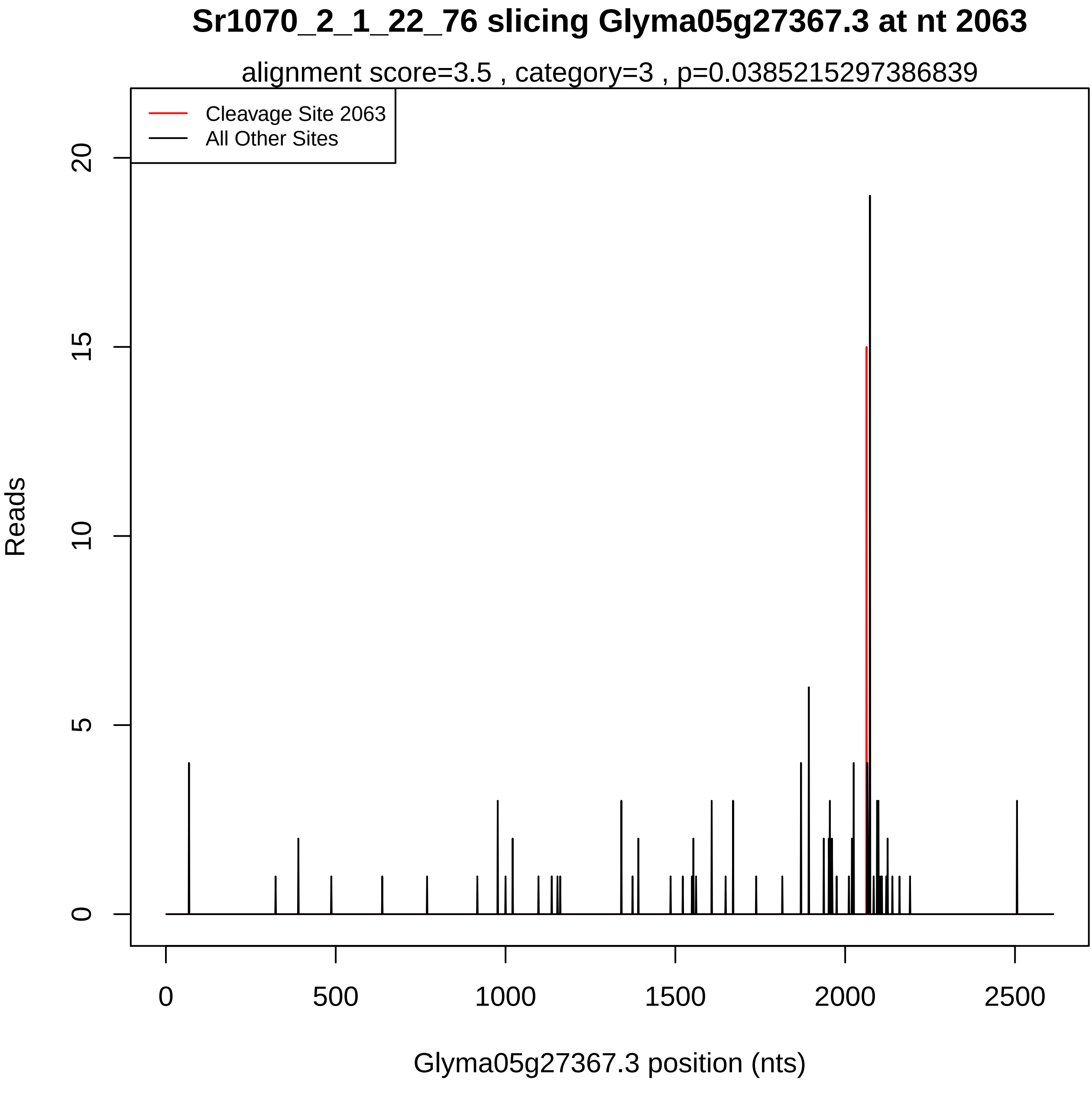

Sr1070_2_1_22_76 slicing Glyma05g27367.3 at nt 2063
alignment score=3.5 , category=3 , p=0.0385215297386839
Cleavage Site 2063
All Other Sites
Reads
0
5
10
15
20
0
500
1000
1500
2000
2500
Glyma05g27367.3 position (nts)

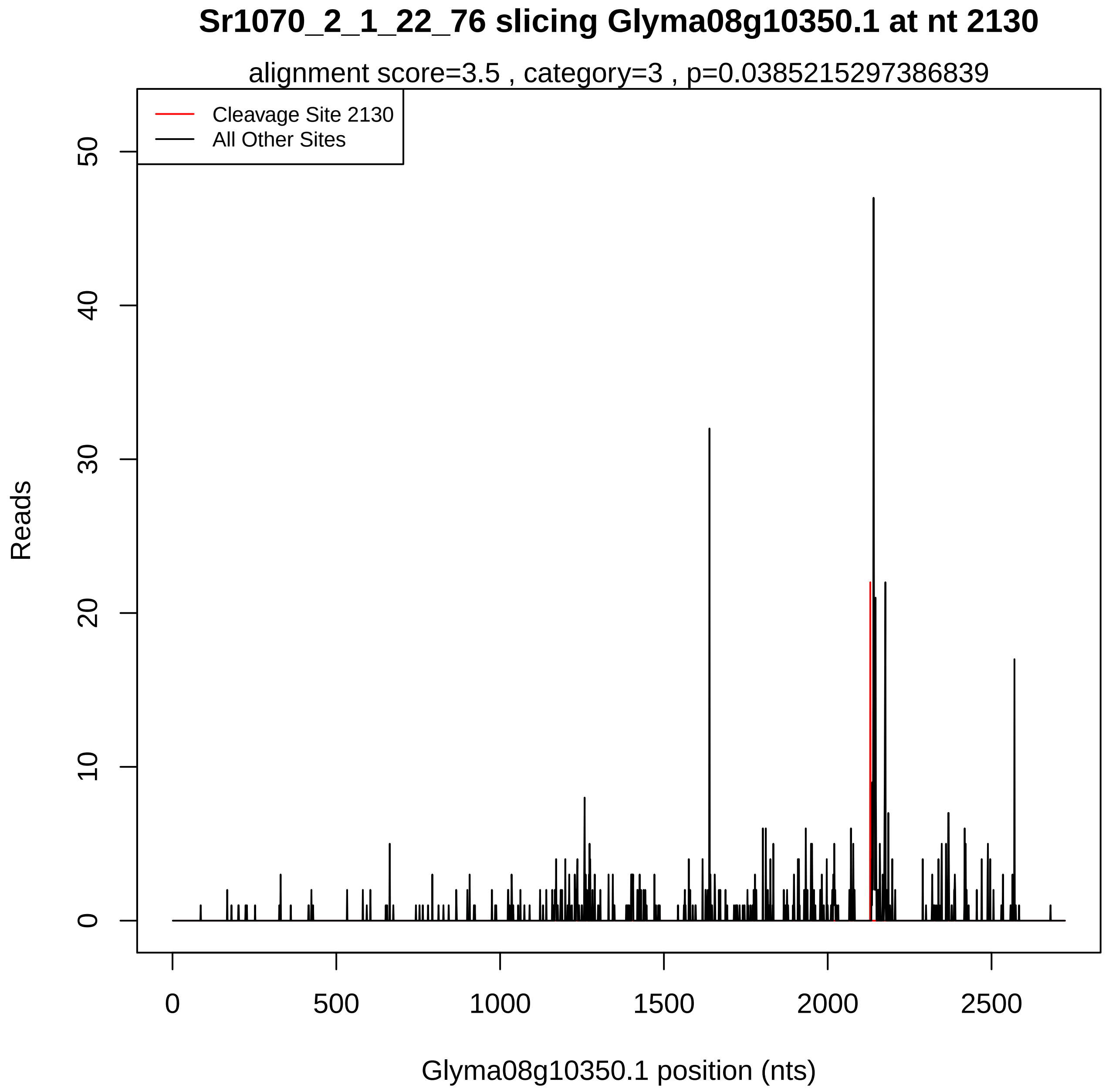
Sr1070_2_1_22_76 slicing Glyma08g10350.1 at nt 2130
alignment score=3.5 , category=3 , p=0.0385215297386839
Cleavage Site 2130
All Other Sites
Reads
0
10
20
30
40
50
0
500
1000
1500
2000
2500
Glyma08g10350.1 position (nts)

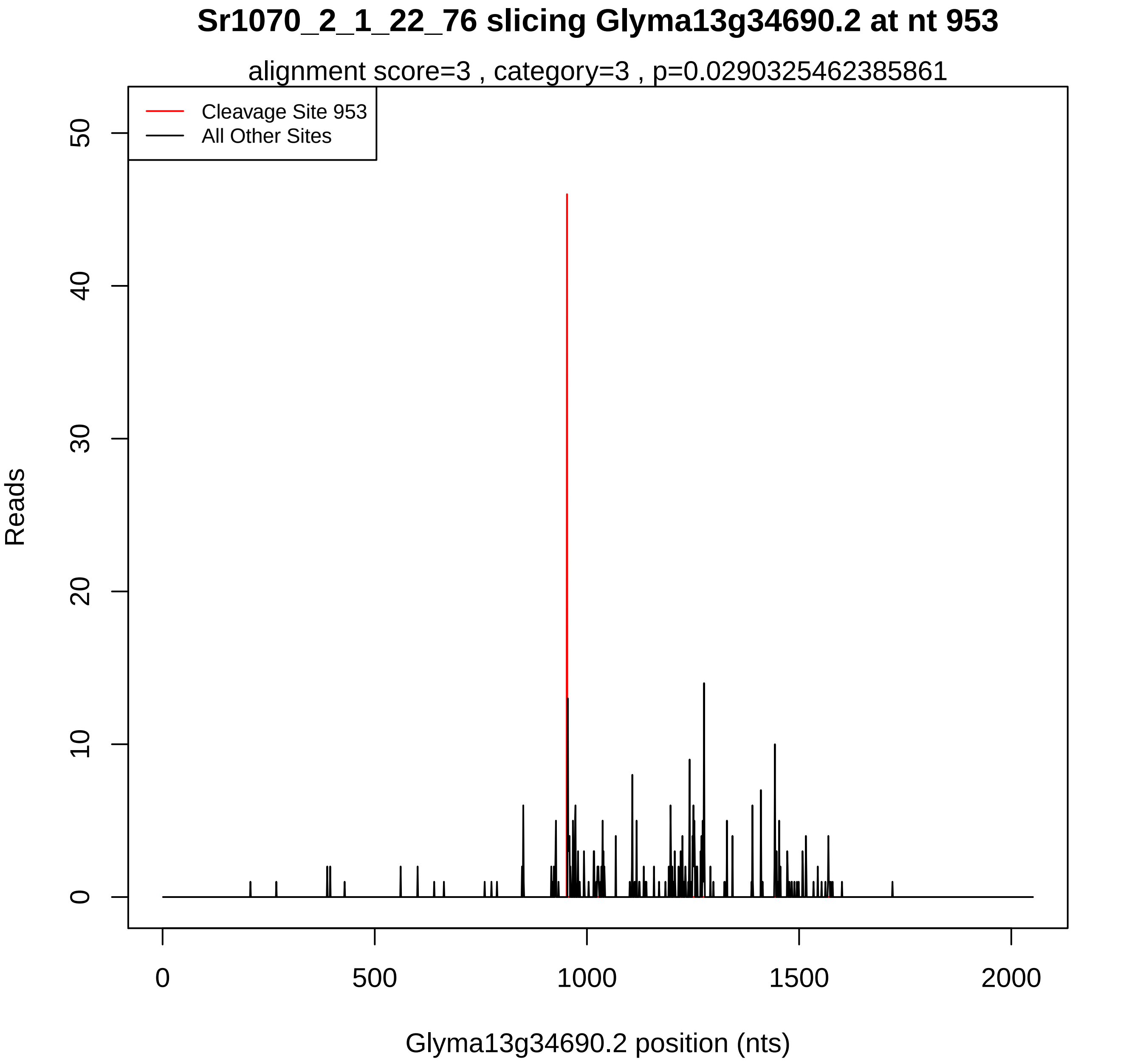

Sr1070_2_1_22_76 slicing Glyma13g34690.2 at nt 953
alignment score=3 , category=3 , p=0.0290325462385861
Cleavage Site 953
All Other Sites
Reads
0
10
20
30
40
50
0
500
1000
1500
2000
Glyma13g34690.2 position (nts)

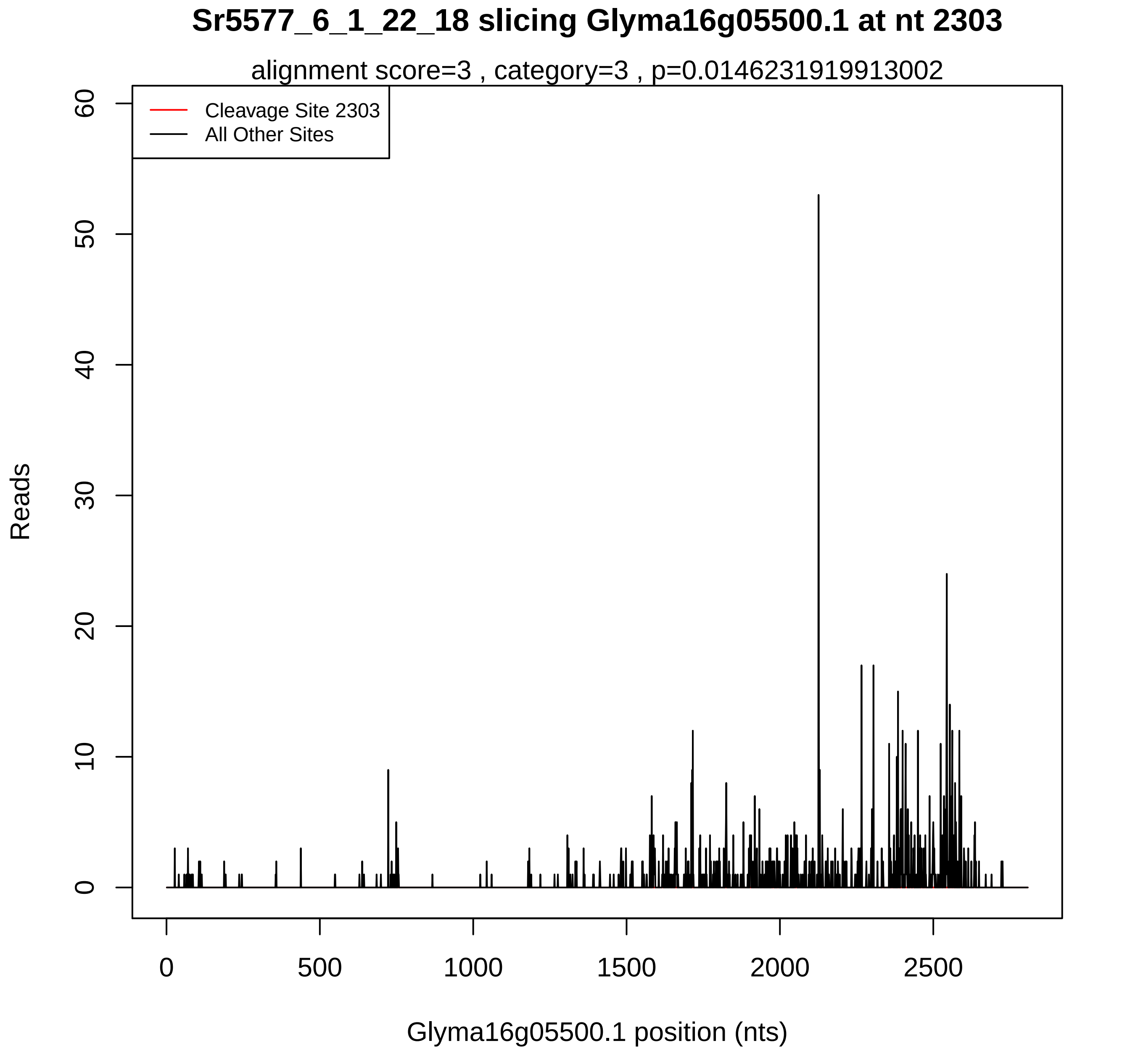
Sr5577_6_1_22_18 slicing Glyma16g05500.1 at nt 2303
alignment score=3 , category=3 , p=0.0146231919913002
Cleavage Site 2303
All Other Sites
Reads
0
10
20
30
40
50
60
0
500
1000
1500
2000
2500
Glyma16g05500.1 position (nts)

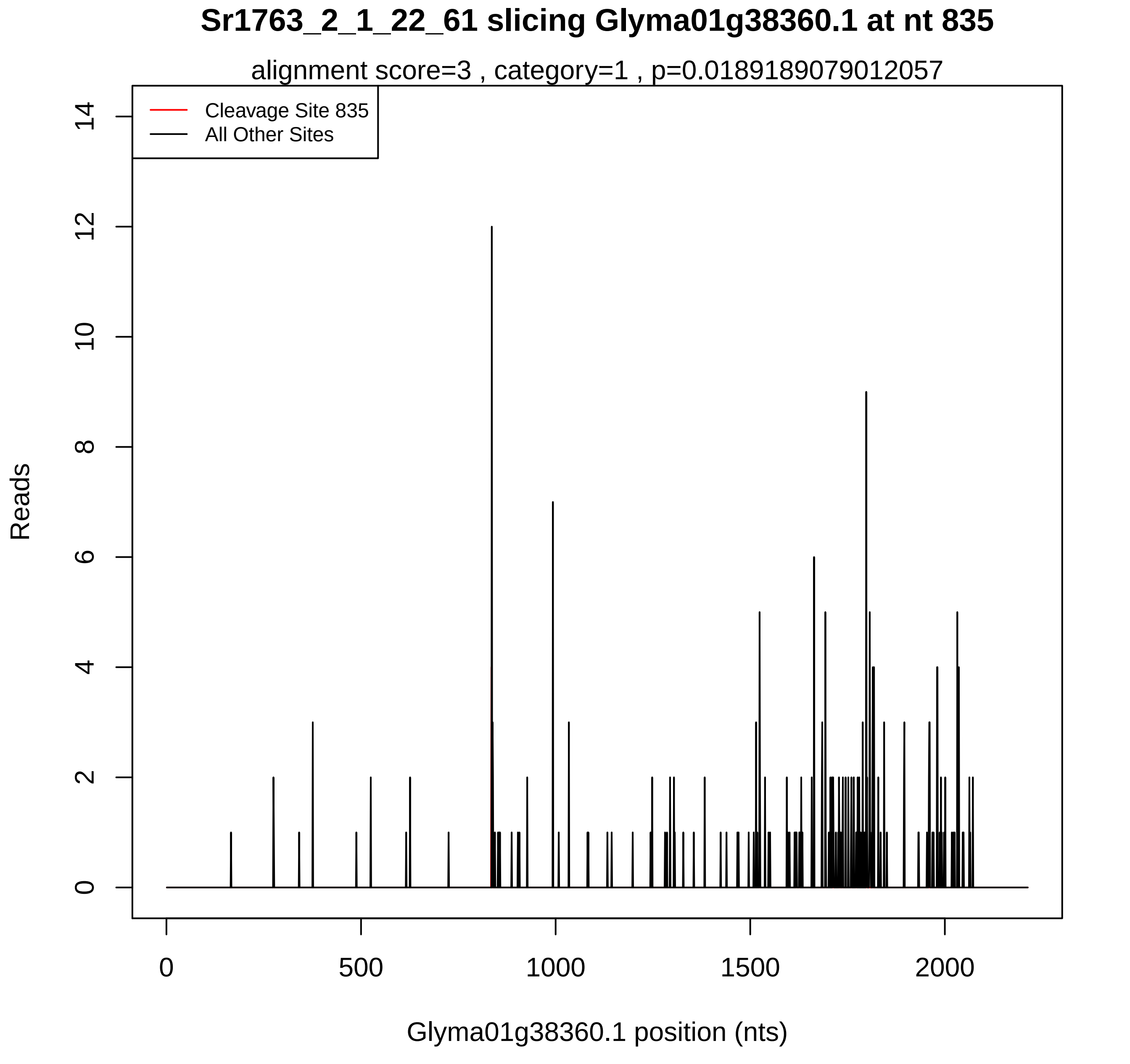

Sr1763_2_1_22_61 slicing Glyma01g38360.1 at nt 835
alignment score=3 , category=1 , p=0.0189189079012057
Cleavage Site 835
All Other Sites
Reads
0
2
4
6
8
10
12
14
0
500
1000
1500
2000
Glyma01g38360.1 position (nts)

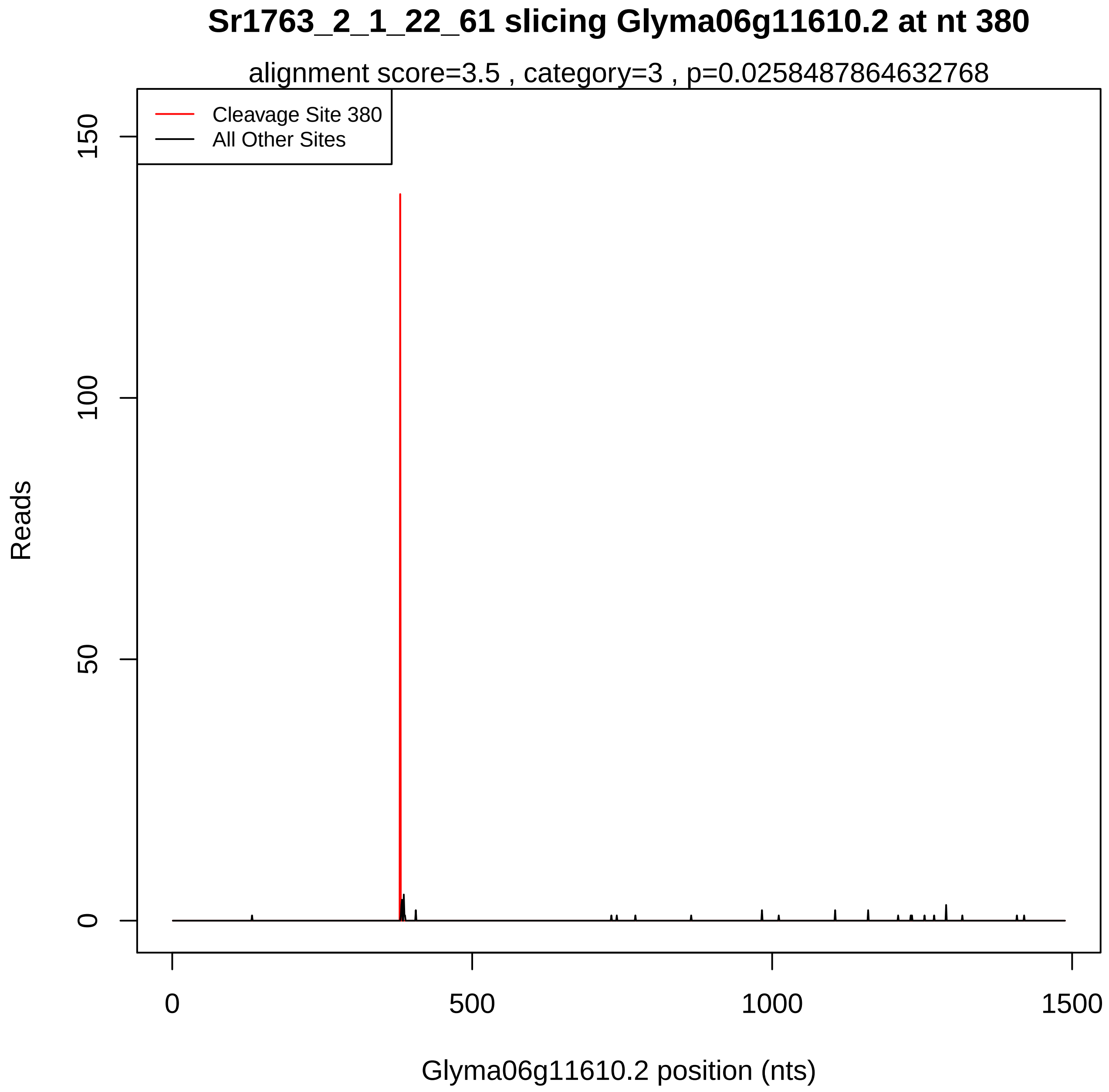
Sr1763_2_1_22_61 slicing Glyma06g11610.2 at nt 380
alignment score=3.5 , category=3 , p=0.0258487864632768
Cleavage Site 380
All Other Sites
Reads
150
100
50
0
500
1000
1500
Glyma06g11610.2 position (nts)

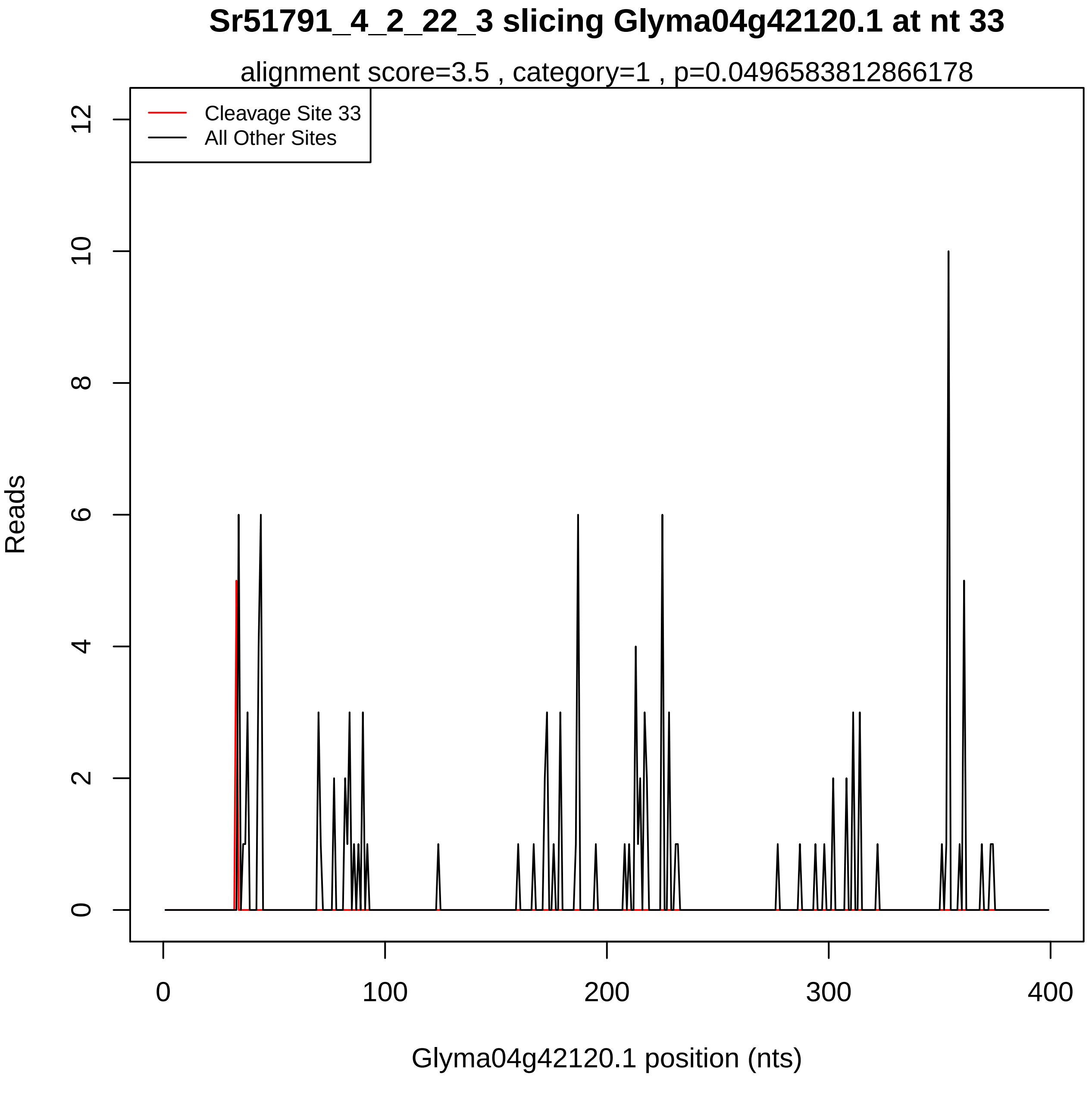
Sr51791_4_2_22_3 slicing Glyma04g42120.1 at nt 33
alignment score=3.5 , category=1 , p=0.0496583812866178
Cleavage Site 33
All Other Sites
Reads
0
2
4
6
8
10
12
0
100
200
300
400
Glyma04g42120.1 position (nts)

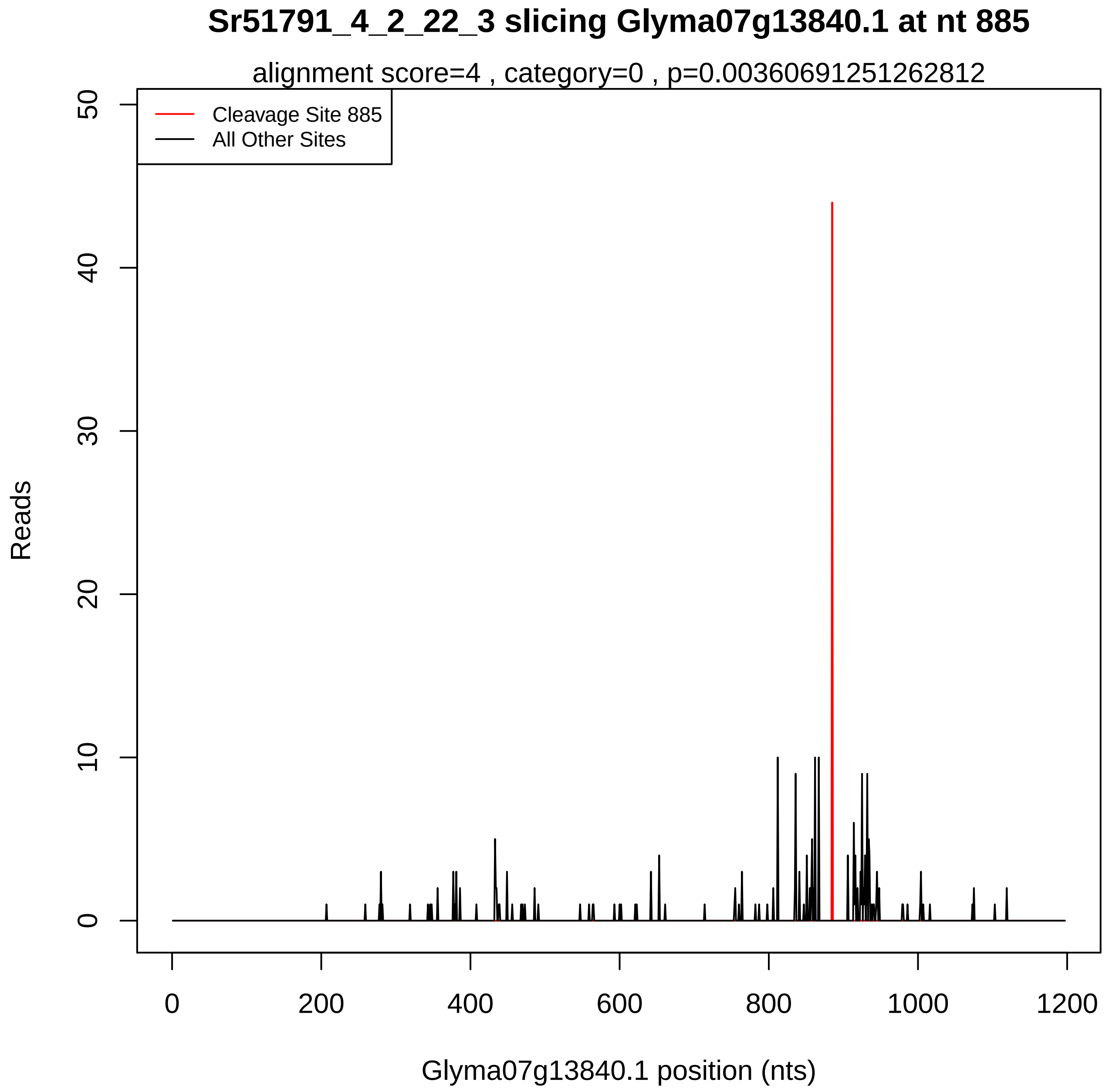
Sr51791_4_2_22_3 slicing Glyma07g13840.1 at nt 885
alignment score=4 , category=0 , p=0.00360691251262812
Cleavage Site 885
All Other Sites
Reads
0
10
20
30
40
50
0
200
400
600
800
1000
1200
Glyma07g13840.1 position (nts)
