# Supplementary material for: Novel MiRNA and PhasiRNA Biogenesis Networks in Soybean Roots from Two Sister Lines That Are Resistant and Susceptible to SCN Race 4
Source: PLoS One. 2014 Oct 30;9(10):e110051. doi: 10.1371/journal.pone.0110051 (PMC4214822; doi:10.1371/journal.pone.0110051)
Supplement: Figure S5 — The small RNAs corresponding to the miRNA targets. The abundance of each secondary siRNAs is plotted (A). The phasing secondary siRNAs corresponding to the miRNA cleavage sites are highlighted in red. The miRNA complementary sites are shown with red arrows. The length distribution is plotted on the right (B). The phasing radial graph is represented next to this (C). Each spoke of the radial graph represents 1 of the 21 phasing registers, with the total number of sRNAs mapping to that register plotted as distance from the center. A, sense transcript; AS, antisense transcript. (PDF) [file pone.0110051.s005.pdf]

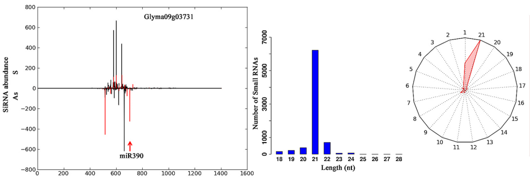
Glyma09g03731
SiRNA abundance
S
As
miR390
800
600
400
200
0
−200
−400
−600
−800
0
200
400
600
800
1000
1200
1400
1600
Number of Small RNAs
Length (nt)
18
19
20
21
22
23
24
25
26
27
28
0
1000
3000
5000
7000
1
2
3
4
5
6
7
8
9
10
11
12
13
14
15
16
17
18
19
20
21

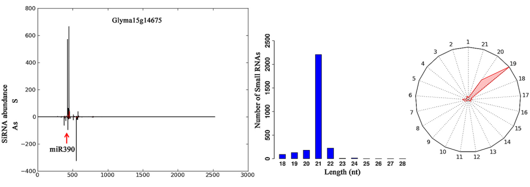
Glyma15g14675
SiRNA abundance
S
As
miR390
Number of Small RNAs
Length (nt)

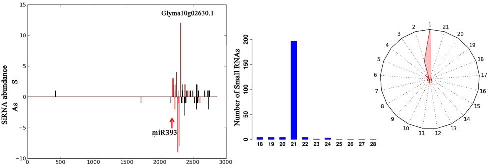

Glyma10g02630.1
SiRNA abundance
S
As
miR393
Number of Small RNAs

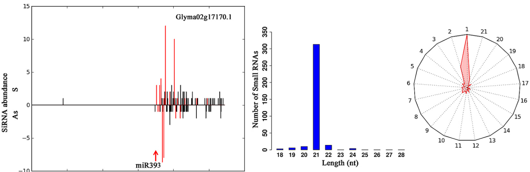
Glyma02g17170.1
SiRNA abundance
S
As
miR393
Number of Small RNAs
Length (nt)
15
10
5
0
−5
−10
0
500
1000
1500
2000
2500
3000
350
300
250
200
150
100
50
18
19
20
21
22
23
24
25
26
27
28
1
2
3
4
5
6
7
8
9
10
11
12
13
14
15
16
17
18
19
20
21

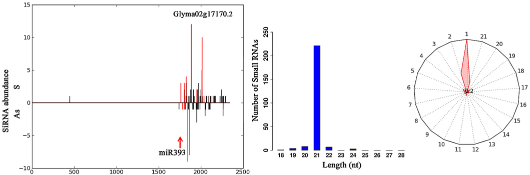

Glyma02g17170.2
SiRNA abundance
S
As
miR393
Number of Small RNAs
Length (nt)

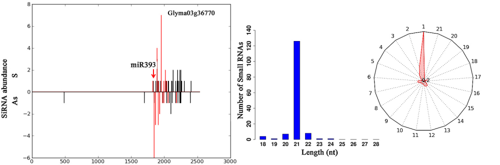

SiRNA abundance
S
As
Glyma03g36770
miR393
Number of Small RNAs
Length (nt)

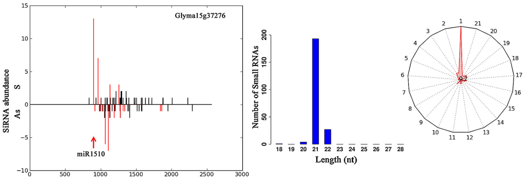

Glyma15g37276
SiRNA abundance
S
As
miR1510
Number of Small RNAs
Length (nt)

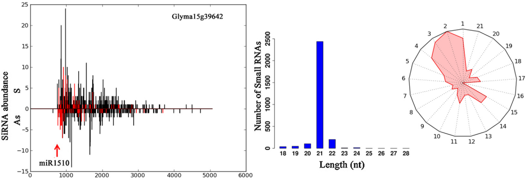
Glyma15g39642
SiRNA abundance
S
As
miR1510
25
20
15
10
5
0
−5
−10
−15
0
1000
2000
3000
4000
5000
6000
Number of Small RNAs
2500
2000
1500
1000
500
0
18
19
20
21
22
23
24
25
26
27
28
Length (nt)
1
2
3
4
5
6
7
8
9
10
11
12
13
14
15
16
17
18
19
20
21

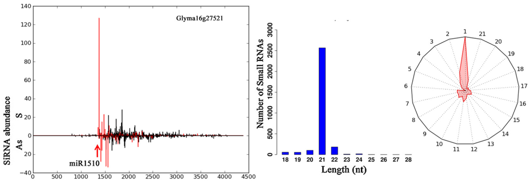

Glyma16g27521
SiRNA abundance
S
As
miR1510
Number of Small RNAs
Length (nt)
